# On the usage of health records for the design of virtual patients: a systematic review

**DOI:** 10.1186/1472-6947-13-103

**Published:** 2013-09-08

**Authors:** Marcus D Bloice, Klaus-Martin Simonic, Andreas Holzinger

**Affiliations:** 1 Institute for Medical Informatics, Statistics and Documentation, Medical University of Graz, Auenbruggerplatz 2, A-8036 Graz, Austria; 2 Institute of Information Systems and Computer Media, Graz University of Technology, Inffeldgasse 16c, A-8010 Graz, Austria

**Keywords:** Virtual patients, Electronic health records, Decision making

## Abstract

**Background:**

The process of creating and designing Virtual Patients for teaching students of medicine is an expensive and time-consuming task. In order to explore potential methods of mitigating these costs, our group began exploring the possibility of creating Virtual Patients based on electronic health records. This review assesses the usage of electronic health records in the creation of interactive Virtual Patients for teaching clinical decision-making.

**Methods:**

The PubMed database was accessed programmatically to find papers relating to Virtual Patients. The returned citations were classified and the relevant full text articles were reviewed to find Virtual Patient systems that used electronic health records to create learning modalities.

**Results:**

A total of n = 362 citations were found on PubMed and subsequently classified, of which n = 28 full-text articles were reviewed. Few articles used unformatted electronic health records other than patient CT or MRI scans. The use of patient data, extracted from electronic health records or otherwise, is widespread. The use of unformatted electronic health records in their raw form is less frequent. Patient data use is broad and spans several areas, such as teaching, training, 3D visualisation, and assessment.

**Conclusions:**

Virtual Patients that are based on real patient data are widespread, yet the use of unformatted electronic health records, abundant in hospital information systems, is reported less often. The majority of teaching systems use reformatted patient data gathered from electronic health records, and do not use these electronic health records directly. Furthermore, many systems were found that used patient data in the form of CT or MRI scans. Much potential research exists regarding the use of unformatted electronic health records for the creation of Virtual Patients.

## Background

Much research has shown that Virtual Patients are a credible and effective form of teaching, and they have been shown to improve knowledge retention, student participation, and other factors [1,2]. However, they are also expensive and time consuming to produce, resulting in lower adoption rates than might be expected [3]. Reducing the costs of producing Virtual Patients, while still maintaining their efficacy as a teaching tool, is paramount to their successful acceptance and adoption, especially for institutions in less developed countries were cost issues are more inhibitive. In an attempt to reduce the costs of producing Virtual Patients, our group began experimenting with the notion of developing a tablet-based application for the Apple iPad that would base Virtual Patients on real, unformatted, annotated electronic health records [4]. In order to gain an understanding of previous work carried out in this field, a literature review was conducted to investigate Virtual Patients and the role of the electronic health record in their construction. More specifically, our group wished to ascertain whether previous work had concentrated on producing learning material using electronic health records gathered from hospital information systems. Further, we wished to learn if there has been any precedent in using annotated patient records for the purposes of teaching. Last, because interoperability and collaboration are paramount to reducing costs, we wished to investigate if any file formats for the exchange of Virtual Patients have been suggested or proposed by any research groups, or if any existing standards, such as SCORM (Sharable Content Object Reference Model) are in widespread use. The use of standards is important for the ability to allow for learning modules to be shared across medical centres worldwide.

Before proceeding, it is important to define the terminology used throughout this paper. This paper discusses unformatted electronic patient records which we describe as patient records which have been retrieved from a hospital information system in their raw form. These could be lab reports, medical examination reports, X-Rays, electrocardiograms (ECGs), and so on. Many Virtual Patients are based on patient data, yet often they do not use electronic health records in their raw form; rather, data are extracted from these records and reformatted for use in a Virtual Patient user interface.

By annotated patient records, we describe any electronic patient records which have been subsequently tagged with meta-data or some descriptive text. For example, a patient record may contain an acronym that is not widely known, and such a patient record may be annotated with meta-information describing the meaning of the acronym in order to aid the learner. Scanned hand-written documents which have been annotated with an electronic version of the document’s text would also be considered an annotated patient record.

Last, it is important to define what is meant by the term “Virtual Patient” itself. According to the European Commission co-funded Electronic Virtual Patients (eViP) project, a Virtual Patient is “an interactive computer simulation of real-life clinical scenarios for the purpose of medical training, education or assessment”. This definition covers all electronic Virtual Patients, however for the purposes of this review this definition has been expanded to include other forms of virtual patient, including hardware simulators, mannequins, and videos. This was done in order to remain as flexible as possible when deciding on which abstracts should be accepted for the review.

## Methods

The PubMed database was queried programmatically using the E-utilities API made available by the National Center for Biotechnology Information (NCBI). A small Java program was developed to access PubMed using the search term *“Virtual Patient” OR “Virtual Patients”*, resulting in n = 362 returned results. A standard free-text query was performed as no MeSH term for *Virtual Patient* exists. The results, returned by the API in XML format, were subsequently stored in an OpenOffice.org database for reviewing. Of the fields returned by the E-utilities API, the Authors, PubMed ID, Title, Affiliations, Year, Month, and Abstract fields were stored in the database while the rest were discarded. The citations were last extracted on the 10th of January 2013.

The Java application was also designed to export all citations as an HTML file which includes hyperlinks to each PubMed abstract (see Additional file 1).

The literature review was performed in four steps. The initial step was to review all abstracts that were retrieved from PubMed, discarding those that were unsuitable. The second phase was to categorise all the suitable abstracts and to tag each abstract with various attributes which are described below. The third phase involved gathering information regarding the categorisation of each of the abstracts by running SQL queries on the tagged citations stored in the OpenOffice.org database. The fourth and final phase was to review the full text articles that described using Virtual Patients based on patient data or electronic health records.

**Figure 1:**
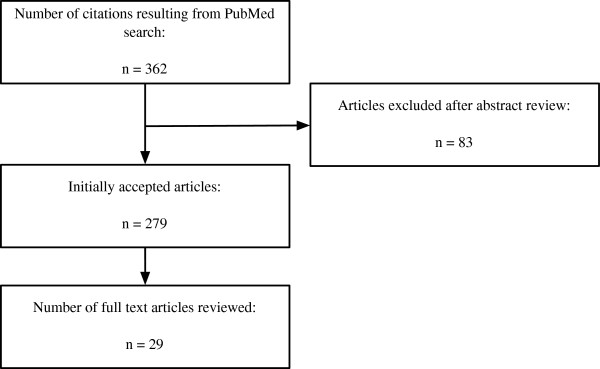
**Abstract dlowchart.** The procedure followed during the abstract and manuscript reviewing process.

A flowchart of the abstract selection procedure can be seen in Figure 1.

### Phase 1

In phase 1, each of the 362 citations returned by the NCBI web service was reviewed for suitability. In this phase, only those citations where no abstract was available, or where the abstracts were simply too short to make a fair judgement, were rejected. In total, n = 279 abstracts were considered to be suitable while n = 83 were rejected. In phase 2, the remaining 279 abstracts were tagged with keywords in order to categorise them suitably for the final phases of the review.

### Phase 2

The second phase of the review consisted of tagging each of the remaining 279 abstracts with keywords in order to categorise them. This was performed in order to get a general understanding of how Virtual Patient development efforts are distributed worldwide. During this process, each citation was tagged with the attributes seen in Table 1.

**Table 1:** Phase 1 categorisation

**Attribute**	**Value**
Suitable	*True* or *false*
Teaching type	Either *undergraduate*, *graduate*, or *NA*
Article type	Either *software*, *hardware*, *model*, or *report*
Patient records	*True* or *false*

Each abstract was tagged with attributes during the second phase of the review for categorisation purposes.

The *Suitable* field was used to denote the suitability of the abstract for further tagging, therefore citations with missing abstracts or abstracts that were deemed as being too short were marked as being unsuitable. The *Teaching Type* attribute could be one of either *Graduate*, *Undergraduate*, or *NA*. Teaching systems that were intended for both advanced level undergraduates and graduates were marked as being undergraduate systems. *NA* was reserved for systems, such as bio-simulations, that were not designed for teaching. The *Article Type* attribute referred to the type of Virtual Patient system that was being reported in the article. Systems that utilised both hardware and software were marked as software systems. *Reports* denoted any papers where a third party system was being tested or evaluated, for example papers that described using the Web-SP system. The *model* attribute was used to denote any Virtual Patient systems that modelled or simulated virtual populations or physiological systems such as bio-simulations of diabetic patients. The *Patient Records* attribute is a Boolean value that denotes whether the abstract explicitly mentioned using real patient data in the design of the Virtual Patient. This attribute was used to preselect the articles that would be included in the full-text review.

### Phase 3

This phase involved executing a number of SQL queries on the tagged citations. This was done to gain an understanding of how the development of Virtual Patients is distributed worldwide.

### Distribution of systems

**Figure 2:**
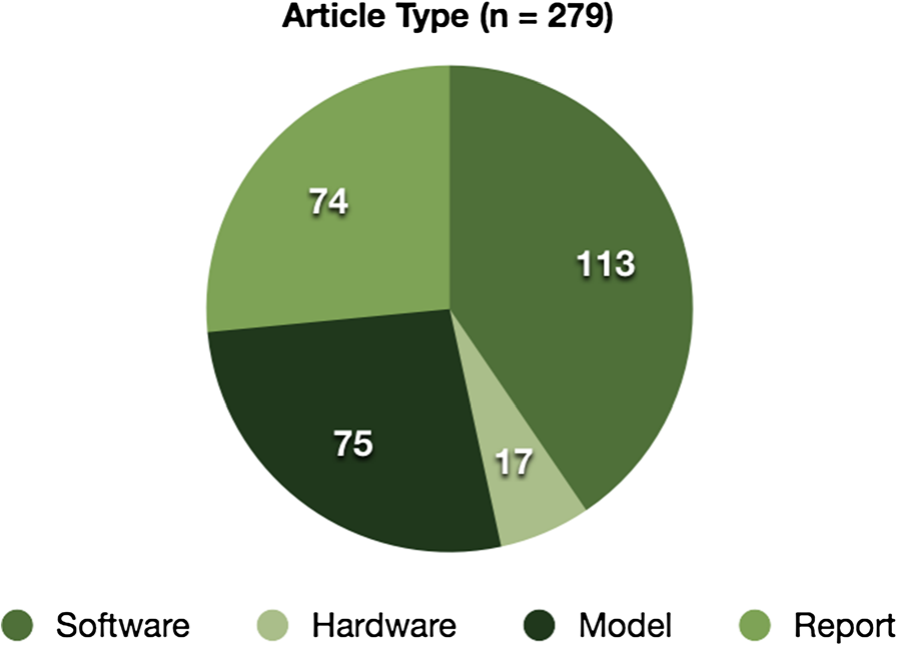
**Distribution of article types.** The suitable abstracts were distributed as follows: 113 (≈40%) citations were tagged as being software systems, 17 (≈6%) were tagged as being hardware only systems, 75 (≈27%) were tagged as being models of some kind, such as bio-simulations, and 74 (≈27%) were reports including reviews and papers on 3rd party system adoption.

Of the 279 citations that were accepted beyond phase 1 of the review, approximately 40% were found to be discussing software-based Virtual Patients, as seen in Figure 2.

### Distribution of teaching types

**Figure 3:**
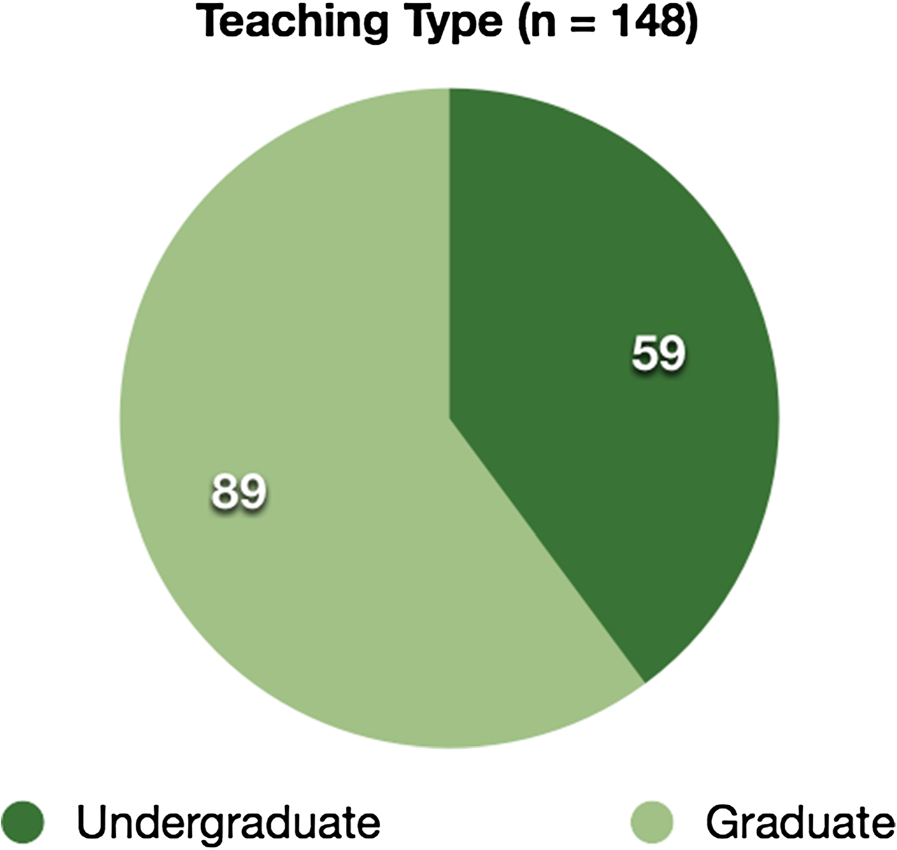
**Distribution of teaching types.** The distribution of the teaching types.

A total of 148 systems were classified as being teaching systems. Of those, approximately 60% were used to teach undergraduate students and 40% were used to teach or train graduates, as shown in Figure 3. Only articles that were used for teaching or training were considered for the final phase of the review.

### Phase 4

In this final phase of the review, the full-text articles for all citations that were categorised as having used patient records were retrieved. Each article was then read to find evidence for the use of electronic health records or real patient data for the development of Virtual Patients. The Results section describes the outcome of this phase of the review in detail.

## Results

A total of 28 full-text articles were retrieved for the final review phase, the results of which are described in this section. For this section, the reviewed papers have been subdivided into those that use patient data in the form of medical images, and those that use other forms of patient data and electronic health records such as lab reports, electrocardiograms, physician notes, and so on. This was done, first to reduce the set of papers further (as we wished to find papers that use all forms of electronic health record and do not solely use medical imaging data), and to aid in readability. We expand on this point in the Discussion section.

A summary of each full-text article can be seen in the table in Additional file 2.

### Virtual patients based on medical image data

A large proportion of the full-text articles reviewed described systems that based Virtual Patients on CT or MRI data only. Many of these articles focussed on creating 3D visualisations of regions of interest using existing medical imagery.

In 2009, Jacobson et al. reported on the creation of Virtual Patients through the use of CT images of cadavers [5]. 3D reconstructions were created using the Osirix DICOM viewer, and subsequently exported as QuickTime movies for students to view. A similar method was employed by Parikh et al. (2009) who describe the use of CT scans to reconstruct 3D areas for use in a virtual surgery environment for preoperative planning. Their system was not intended for teaching purposes; rather it was a training platform [6]. This was similarly discussed in 2009 by King et al., where medical imaging data was used to reconstruct 3D regions. This was done to optimise port placement for in vivo biosensors [7]. Porro et al., in 2005, used recorded clinical data in the form of DICOM images, either from recent patients or from archives, to create 3D reconstructions [8]. In 2005 IM Heer et al. discussed a training device that allows for virtual training of ultrasound cases [9]. Research carried out in 2002 by Michel et al. discusses a virtual reality system for training endourological procedures. Patient data were obtained from CT and MRI scans [10]. Freysinger et al. [11] described a 3D virtual reality system where CT and MR data sets were used to create the 3D renderings, similar to the work performed by Porro, King, Jacobson, and Parikh. Wolfram Lamadé’s group discussed 3D modelling of CT scans from their paper of 2000. A number of Virtual Patients (a total of 7) were created from this data to test if liver surgery planning could be improved using 3- and higher-D representations [12]. Finally, in an article for the European Journal of Ultrasound, H.H. Ehricke addressed sonography education where ultrasound simulation was employed. The article describes an extensible case database, where cases could be continually added to a pool. The cases consisted of 3D data sets, which were acquired either from patients or healthy subjects. The cases also included textual case descriptions in the form of annotations [13]. In the context of this review, Ehricke’s article matches closely the type of work our group were most interested in finding; the platform described in the paper allows for interchangeable cases and the cases are annotatable.

What is apparent is that the vast majority of Virtual Patients that are based on medical image data are 3D representations that are reconstructed from exiting patient CT or MRI data. This should come as no great surprise to those who are active in the area of medical software simulation. However, of special interest to our group were any articles that reported on Virtual Patient development where several types of medical documentation were used. The next section describes any papers that were found that match this criterion.

### Virtual patients based on patient data

While the majority of the systems described in the previous section used quite similar approaches for creating cases, the Virtual Patients produced using other forms of patient data were more varied in their design and implementation. Due to the variety of their approaches, the manuscripts could be further subdivided into three categories: Virtual Patients that were used for teaching and training purposes, virtual consultancy systems, and assessment systems.

### Teaching and training

The vast majority of full-text papers reviewed dealt with Virtual Patients used for teaching undergraduate students and for training, and these are described here.

Shyu et al. reported creating Virtual Patients using electronic health records gathered from a hospital information system. Shyu’s group also reported on using the SCORM (Sharable Content Object Reference Model) standard to aid the sharing and transfer of cases between medical centers [14]. Similary, Trace et al. (2012) describe a system where students authored electronic cases. Students gathered patient data and created Virtual Patients using a custom PowerPoint template. This paper highlights how, by using patient data gathered from hospital information systems, Virtual Patients can be made efficiently while maintaining their efficacy as a valid teaching tool [15]. In 2009, Ullrich et al. described a 3D simulator that used MRI scans. By using an XML-based database, the ability to create cases involving arbitrary scenarios was possible, allowing for a subject database to be created [16]. In an article reported in the Studies in Health Technology and Informatics journal, Oliven et al. describe a web-based Virtual Patient application. The system allows for natural language processing whereby students may ask open questions and receive answers, allowing them to request lab reports or other clinical data [17]. Abendroth et al. describe the use of original documents to create Virtual Patients that were integrated into the CASUS system [18]. In their study, ten patient cases were developed that were based on electronic health records, in an attempt to better decision-making skills. The group used anonymised patient records and laboratory results to create the cases, and they were annotated to provide feedback to students regarding hypothesis refinement and background information. A self-assessment questionnaire was used gather student satisfaction levels, although the authors admitted that too few responded to be able to make any real claims as to their method’s efficacy. In 2012, Pinnock et al. described an eLearning system entitled evPaeds in The Clinical Teacher. Cases were created for their system using real examinations notes, history notes, test results, and X-rays by expert clinicians, who also provided feedback and meta-data for the students who use the system. The group provided solid arguments as to why using real patient data is desirable in the production of Virtual Patients, which reiterate our group’s statements [19]. In 2011, Hörnlein et al. outlined a system known as CaseTrain. This system allows students to examine patients and answer questions relating to the patient as the case develops. Electronic health records themselves are not used directly, as the interface is based on Flash and patient records are adapted to suit the interface. The system allows for interchangeable cases, which are prepared in Word format before being imported into the system [20]. In 2011, Edelbring et al. described a series of Virtual Patients based on authentic rheumatology patients created through patient interviews, text, laboratory results, and so on. A total of four Virtual Patients were created in this way, and they were accessible using a system known as ReumaCase. Therefore, the system created used patient cases that were interchangeable, yet they were not based primarily on electronic health records and could potentially incur long production times [21]. Video enhanced Virtual Patients were the subject of work performed by Adams et al. in 2011. The Virtual Patients were again produced to run on a 3rd party system, in this case the Quandary platform. [22]. In Medical Teacher, Poulton et al. (2009) discuss the replacement of paper cases using Virtual Patients. The new Virtual Patients were designed using the VUE system (Tufts University’s Visual Understanding Environment) and subsequently transferred to the OpenLabyrinth system (an open source version of the Labyrinth software). Therefore, the cases were interchangeable and the cases were based on real patients. However, the electronic health records themselves were not used as all patient data were reformatted for use with the OpenLabyrinth architecture [23]. Hooper et al. discuss using Virtual Patients to study any variation in depression care and decision making among physicians. The group constructed 32 CD-ROM Virtual Patients. The group specifically aimed to answer whether or not the Virtual Patients they created were believable. The vignettes they created required using actors, although some of the scripts they used were based on actual physician-patient encounters. The group spent 12 months ensuring the cases were operational and believable. This shows once again the efforts that are often required to create and produce Virtual Patients. 90% of the physicians using the system either agreed or strongly agreed when asked about whether the Virtual Patients seemed real to them [24]. In 2008, Vukanovic-Criley et al. reported using recordings of patients at the bedside, along with actual heart sounds, to train cardiac examinations. The patients’ real echocardiograms, chest X-rays, and lab reports were also used in the creation of the Virtual Patients. Real patient data, therefore, was used extensively; however the Virtual Patients were nonetheless produced (due to the recordings at the bedside, recordings of the heart murmurs, and so on) and required extensive effort to create [25]. In Medical Teacher, Dewhurst et al. once again describe work in which Virtual Patients were collaboratively developed, where a total of 20 cases were developed in this way. Again, this group created their storyboards using the VUE system and imported them into the Labyrinth software. Due to this, the cases created were interchangeable, allowing for collections, or pools, of cases to be built [26]. Schittek Janda et al. describe a Virtual Patient system used in oral health care. The system created was generic, and cases from other medical fields could be imported into it. The system is novel in that it does not provide the student with a list of options from which to choose when input is required. Instead, the student should make decisions using free-text input when, for example, requesting clinical records. This was similar to the work carried out by Oliven et al. Patient data is used throughout, although it had been reformatted to suit the web-based interface [27].

### Virtual consultancy

Two papers used Virtual Patients to create virtual consultancies. Wood et al. created a Virtual Consulting Room that allowed doctors to view the progress of patients no longer in their care. The system was intended to be used primarily by junior doctors in order to understand the rationale behind clinical decision-making [28]. Smith et al. performed similar work in their paper for Medical Teacher in 2007. The authors describe Patient-Centred Learning, an evolution of Problem-Based Learning, through the use of high-fidelity Virtual Patients. The group described using the electronic health record, modified for educational purposes, to create a virtual practice. These electronic health records can also link to electronic learning resources. Interestingly, the authors describe the real-time arrival of new data for Virtual Patients, yet the test students were not convinced of the usefulness of this feature. Students preferred to be able to “look ahead” at the patient’s eventual outcome. The students in the study were undergraduate students [29].

### Assessment

Three further papers described the use of Virtual Patients for assessment purposes. Gunning et al. describe a system where Virtual Patients were used for the assessment of students who completed a case-based learning course. The group mentioned one patient case in detail, which consisted of 25 physical exams, 25 lab or imaging tests, and so on. Therefore, the group used a broad range of electronic health records for the creation of their Virtual Patients [30]. Courteille et al. report on the use of a single Virtual Patient as an assessment tool [31], while Subramanian et al. used a web-based medical learning modality that allowed students to treat a memorable Virtual Patient in a case-based format to test its effectiveness in comparison to a traditional lecture-based format. Two groups of students were assessed three weeks after participating in either a lecture-based modality or the Virtual Patient-based modality. Significant improvements were recorded for the group that used the Virtual Patient [32].

## Discussion

Creating Virtual Patients requires considerable effort both monetarily and in terms of person hours. A survey of U.S. and Canadian medical schools by Huang et al. found that the development of Virtual Patients costs are high, with 85% of Virtual Patients costing more than $10,000 to produce and taking an average of 16.6 months to complete. The group also found that the vast majority of Virtual Patients are media-rich, which is partly responsible for these very high costs [3].

However, much research has shown that the development of sound clinical reasoning skills and decision-making abilities is inextricably linked to experience. More specifically, students should encounter multiple, similar cases where subtle variations in patient presentations exist [33]. Through contact with many, subtly different patients, students begin to learn how to build “illness scripts”. As stated by Norman et al., it is “the power of the plural” which is key to learning decision-making skills [34]. However, if Virtual Patients and Virtual Patient cases are so costly to develop, it is unlikely that the plurality of cases required can realistically be generated for a student to develop such depth of reasoning. Again, it is argued that that for a student to develop good decision-making skills, they must encounter variations of prototypical patient presentations, with each presentation differing only slightly but with largely varying outcomes. For this to be achieved through the use of Virtual Patients, pools of cases are required. However, as can be seen from the work of Huang et al., such archives would be difficult and costly to create through the use of produced, media-rich Virtual Patients. Therefore, our group began experimenting with the notion of creating Virtual Patients using electronic health records that are abundantly available on hospital information systems. This was done with the ultimate aim in reducing the costs and effort needed to produce Virtual Patients in sufficient numbers. To investigate whether there has been any precedent in this field, this literature review was undertaken. We have seen that although patient data is used extensively to create Virtual Patients, it is rare to see electronic health records used in their raw, unformatted state. With the exception of Virtual Patients based on CT or MRI data, most Virtual Patients, that use real patient data, use data which has been extracted from electronic health records and subsequently reformatted to suit the platform upon which they are run. This reformatting constitutes production costs and it is exactly this that our group wishes to avoid by making use of electronic health records directly, without the need for extensive reformatting or data extraction.

This does, however, beg the question: why is the use of patient data, in its raw form, so rare? We believe there are several reasons for this, namely, 1) patient records are still being handwritten and the use of electronic records is far from widespread 2) there are concerns regarding patient data and anonymisation, and 3) they may contain too many gaps or missing pieces of information. The third point may be the most difficult hurdle to surmount, yet it is the belief of the authors that physician annotations would mitigate this issue to a large degree. We also believe that the potential for using annotated health records has not yet been investigated thoroughly. This point will be the subject of future work.

Last, there was little evidence, from the papers reviewed here, of any widespread use of standards such as SCORM to enable inter-institutional collaboration and sharing of Virtual Patients. Several papers describe the ability of their systems or platforms to allow for pools of cases to be accumulated, or that their systems allowed for cases to be imported easily, yet they often failed to mention adhering to any particular standards. That said, projects such as eViP, or the IVIMEDS inter-school Reusable Learning Objects [29], go some way to help increase collaboration by defining standards for Virtual Patient creation.

## Conclusions

This paper reports on the usage of unformatted electronic patient records for the creation of Virtual Patients. This review has shown that while patient data is often used as material for the creation of Virtual Patients, the use of the electronic health record is less prevalent. Of those Virtual Patients that made use of real patient data, most reformatted the data to suit the platform on which they were to be viewed. This reformatting, in itself, requires a considerable amount of time. Due to fragmentation, these Virtual Patients (or Virtual Patient cases) cannot be easily interchanged or exchanged, further hampering interschool collaboration. With the exception of DICOM, standards are not widely adopted.

Our group is of the opinion that there are several advantages to using electronic health records for the creation of Virtual Patients, including not only the aforementioned time and monetary savings. The Casebook application being developed by our group will read electronic health records, organised temporally into cases, which have been annotated with meta-information by the teaching physician. Questions for the student to answer appear between patient records, with the next patient record revealing the answer to the question. Patient records are extracted from a hospital information system and used directly, without any reformatting or data extraction. Using health records directly means that Virtual Patients can be more easily created allowing for pools of cases to be built; important for enhancing clinical reasoning and clinical decision-making. Our group has previously reported on this in detail [4].

## Competing interests

The authors declare that they have no competing interests.

## Authors’ contributions

MDB, KMS, and AH conceived the idea of performing a systematic search of PubMed in order to find Virtual Patient systems that use electronic health records. MDB performed the extraction and reviewed the works. The Java application was written by MDB. The database was administered by MDB. All authors read and approved the final manuscript.

## Pre-publication history

The pre-publication history for this paper can be accessed here:

http://www.biomedcentral.com/1472-6947/13/103/prepub

## Supplementary Material

Additional file 1 **All citations considered for the review (articles.htm).** All citations returned by the E-utilities API are included in this HTML file. The file, articles.htm, can be viewed with any web browser. The file contains links to PubMed for each of the 362 citations included in the review. For copyright reasons, the abstract texts themselves have been removed. Click here for file

Additional file 2 **Table of reviewed articles.** Each of the full-text articles reviewed in the final phase of the review are shown here (Table 2.docx). Here it can be seen if any standards were followed, the type of patient data used to create the Virtual Patient(s), and whether the platform described allowed for cases to be interchanged. Where no standards such as SCORM have been followed, the case format is provided, such as XML or PowerPoint. Click here for file
