# Supplementary material for: On the usage of health records for the design of virtual patients: a systematic review
Source: BMC Med Inform Decis Mak. 2013 Sep 8;13:103. doi: 10.1186/1472-6947-13-103 (PMC3846661; doi:10.1186/1472-6947-13-103)
Supplement: Additional file 1 — All citations considered for the review (articles.htm). All citations returned by the E-utilities API are included in this HTML file. The file, articles.htm, can be viewed with any web browser. The file contains links to PubMed for each of the 362 citations included in the review. For copyright reasons, the abstract texts themselves have been removed. [file 1472-6947-13-103-S1.htm]

Review Abstracts


----------------------------

```
Abstract 1 of 362

1. Clin Teach. 2013 Feb;10(1):51-5. doi: 10.1111/j.1743-498X.2012.00629.x.

Clinical decision making: a pilot e-learning study.

Abendroth M, Harendza S, Riemer M.

Department of Internal Medicine, University Hospital Hamburg-Eppendorf,
GermanyDepartment of Computational Neuroscience, University Hospital
Hamburg-Eppendorf, Germany.

© Blackwell Publishing Ltd 2013.

PMID: 23294745  [PubMed - in process]

Click here for link to PubMed for article 23294745
```

----------------------------

----------------------------

```
Abstract 2 of 362

1. Antimicrob Agents Chemother. 2012 Dec 17. [Epub ahead of print]

A Population Pharmacokinetic Analysis of Fluconazole to Predict Therapeutic
Outcome in Burn Patients with Candida Infection.

Han S, Kim J, Yim H, Hur J, Song W, Lee J, Jeon S, Hong T, Woo H, Yim DS.

Department of Clinical Pharmacology and Therapeutics, Seoul St.Mary's Hospital,
The Catholic University of Korea, 222 Banpodaero, Seochogu, Seoul, Korea.

PMID: 23254425  [PubMed - as supplied by publisher]

Click here for link to PubMed for article 23254425
```

----------------------------

----------------------------

```
Abstract 3 of 362

1. Chem Biol Drug Des. 2013 Jan;81(1):1-4. doi: 10.1111/cbdd.12088.

Beyond the hundred dollar genome - drug discovery futures.

Selwood DL.

The Wolfson Institute for Biomedical Research, University College London, Gower
Street, London, UK.

© 2012 John Wiley & Sons A/S.

PMID: 23253126  [PubMed - in process]

Click here for link to PubMed for article 23253126
```

----------------------------

----------------------------

```
Abstract 4 of 362

1. Acta Oncol. 2012 Dec 17. [Epub ahead of print]

Robustness of IMPT treatment plans with respect to inter-fractional set-up
uncertainties: Impact of various beam arrangements for cranial targets.

Hopfgartner J, Stock M, Knäusl B, Georg D.

Department of Radiooncology, Division of Medical Radiation Physics, Comprehensive
Cancer Center, Medical University Vienna , Vienna , Austria.

PMID: 23244675  [PubMed - as supplied by publisher]

Click here for link to PubMed for article 23244675
```

----------------------------

----------------------------

```
Abstract 5 of 362

1. Perspect Med Educ. 2012 Nov;1(4):162-171. Epub 2012 Oct 2.

Preferred question types for computer-based assessment of clinical reasoning: a
literature study.

van Bruggen L, Manrique-van Woudenbergh M, Spierenburg E, Vos J.

Center for Research and Development of Education, University Medical Center
Utrecht, P.O. Box 85500, 3508 GA Utrecht, the Netherlands.

PMCID: PMC3508269
PMID: 23205341  [PubMed]

Click here for link to PubMed for article 23205341
```

----------------------------

----------------------------

```
Abstract 6 of 362

1. IEEE Trans Biomed Eng. 2012 Nov 15. [Epub ahead of print]

An Online Failure Detection Method of the Glucose Sensor-Insulin Pump System:
Improved Overnight Safety of Type-1 Diabetic Subjects.

Facchinetti A, Del Favero S, Sparacino G, Cobelli C.

PMID: 23193300  [PubMed - as supplied by publisher]

Click here for link to PubMed for article 23193300
```

----------------------------

----------------------------

```
Abstract 7 of 362

1. J Vet Med Educ. 2012 Winter;39(4):368-74. doi: 10.3138/jvme.0212-017R.

Development and preliminary evaluation of student-authored electronic cases.

Trace C, Baillie S, Short N.

PMID: 23187029  [PubMed - in process]

Click here for link to PubMed for article 23187029
```

----------------------------

----------------------------

```
Abstract 8 of 362

1. J Am Board Fam Med. 2012 Nov-Dec;25(6):805-9. doi: 10.3122/jabfm.2012.06.110343.

Diuretic of choice in ABFM hypertension self-assessment module simulations.

Hagen MD, Sumner W, Fu H.

American Board of Family Medicine, Lexington, KY 40511, USA.

PMID: 23136319  [PubMed - in process]

Click here for link to PubMed for article 23136319
```

----------------------------

----------------------------

```
Abstract 9 of 362

1. Med Educ. 2012 Nov;46(11):1111-2. doi: 10.1111/medu.12020.

Effectiveness of a virtual patient program in a psychiatry clerkship.

Lin CC, Wu WC, Liaw HT, Liu CC.

Department of Psychiatry, National Taiwan University Hospital and National Taiwan
University College of Medicine, No.1, Changde St., Taipei 100, Taiwan.

PMID: 23078700  [PubMed - in process]

Click here for link to PubMed for article 23078700
```

----------------------------

----------------------------

```
Abstract 10 of 362

1. J Dent Educ. 2012 Oct;76(10):1365-70.

Autonomous virtual patients in dentistry: system accuracy and expert versus
novice comparison.

Clark GT, Suri A, Enciso R.

Herman Ostrow School of Dentistry, University of Southern California, 925 West 34
St., Los Angeles, CA 90089, USA.

PMID: 23066136  [PubMed - indexed for MEDLINE]

Click here for link to PubMed for article 23066136
```

----------------------------

----------------------------

```
Abstract 11 of 362

1. J Dent Educ. 2012 Oct;76(10):1358-64.

Use of virtual patients in dental education: a survey of U.S. and Canadian dental
schools.

Cederberg RA, Bentley DA, Halpin R, Valenza JA.

University of Texas School of Dentistry at Houston, 7500 Cambridge St., Houston, 
TX 77054, USA.

PMID: 23066135  [PubMed - indexed for MEDLINE]

Click here for link to PubMed for article 23066135
```

----------------------------

----------------------------

```
Abstract 12 of 362

1. Am J Pharm Educ. 2012 Sep 10;76(7):131. doi: 10.5688/ajpe767131.

An online virtual-patient program to teach pharmacists and pharmacy students how 
to provide diabetes-specific medication therapy management.

Battaglia JN, Kieser MA, Bruskiewitz RH, Pitterle ME, Thorpe JM.

PMCID: PMC3448469
PMID: 23049103  [PubMed - in process]

Click here for link to PubMed for article 23049103
```

----------------------------

----------------------------

```
Abstract 13 of 362

1. Acad Psychiatry. 2012 Sep 1;36(5):398-400. doi: 10.1176/appi.ap.10080118.

Virtual patients as novel teaching tools in psychiatry.

Pataki C, Pato MT, Sugar J, Rizzo AS, Parsons TD, St George C, Kenny P.

Dept. of Psychiatry, Keck School of Medicine of the University of Southern
California, Los Angeles, CA, USA.

PMID: 22983473  [PubMed - in process]

Click here for link to PubMed for article 22983473
```

----------------------------

----------------------------

```
Abstract 14 of 362

1. Artif Organs. 2012 Dec;36(12):1026-35. doi: 10.1111/j.1525-1594.2012.01512.x.
Epub 2012 Sep 11.

An educational training simulator for advanced perfusion techniques using a
high-fidelity virtual patient model.

Tokaji M, Ninomiya S, Kurosaki T, Orihashi K, Sueda T.

Department of Surgery, Graduate School of Biomedical Science, Hiroshima
University Department of Cardiovascular Surgery, Faculty of Medicine, Hiroshima
University Department of Clinical Engineering, Hiroshima International
University, Hiroshima Department of Cardiovascular Surgery, Faculty of Medicine
Kochi University, Kochi, Japan.

© 2012, Copyright the Authors. Artificial Organs © 2012, International Center for
Artificial Organs and Transplantation and Wiley Periodicals, Inc.

PMID: 22963152  [PubMed - in process]

Click here for link to PubMed for article 22963152
```

----------------------------

----------------------------

```
Abstract 15 of 362

1. Med Teach. 2013;35(1):e876-84. doi: 10.3109/0142159X.2012.714884. Epub 2012 Sep
3.

Optimal learning in a virtual patient simulation of cranial nerve palsies: The
interaction between social learning context and student aptitude.

Johnson TR, Lyons R, Chuah JH, Kopper R, Lok BC, Cendan JC.

University of Central Florida , USA.

PMID: 22938679  [PubMed - in process]

Click here for link to PubMed for article 22938679
```

----------------------------

----------------------------

```
Abstract 16 of 362

1. Nurs Clin North Am. 2012 Sep;47(3):333-46. doi: 10.1016/j.cnur.2012.05.003. Epub 
2012 Jul 11.

NYU3T: teaching, technology, teamwork: a model for interprofessional education
scalability and sustainability.

Djukic M, Fulmer T, Adams JG, Lee S, Triola MM.

College of Nursing, New York University, 726 Broadway, 10th Floor, New York, NY
10003, USA.

Copyright © 2012 Elsevier Inc. All rights reserved.

PMID: 22920424  [PubMed - indexed for MEDLINE]

Click here for link to PubMed for article 22920424
```

----------------------------

----------------------------

```
Abstract 17 of 362

1. Am J Pharm Educ. 2012 Aug 10;76(6):106. doi: 10.5688/ajpe766106.

European pharmacy students' experience with virtual patient technology.

Cavaco AM, Madeira F.

Faculty of Pharmacy, University of Lisbon, Portugal.

PMCID: PMC3425921
PMID: 22919082  [PubMed - in process]

Click here for link to PubMed for article 22919082
```

----------------------------

----------------------------

```
Abstract 18 of 362

1. Langenbecks Arch Surg. 2012 Aug 10. [Epub ahead of print]

Blended learning in surgery using the Inmedea Simulator.

Funke K, Bonrath E, Mardin WA, Becker JC, Haier J, Senninger N, Vowinkel T,
Hoelzen JP, Mees ST.

Department of General and Visceral Surgery, University Hospital of Muenster,
Waldeyerstr.1, 48149, Muenster, Germany.

PMID: 22878596  [PubMed - as supplied by publisher]

Click here for link to PubMed for article 22878596
```

----------------------------

----------------------------

```
Abstract 19 of 362

1. Stud Health Technol Inform. 2012;180:978-82.

Virtual patient simulation: a comparison of two approaches for capacity building 
in Sub-Saharan Africa.

Bediang G, Raetzo MA, Geissbuhler A.

Department of Radiology and Medical Informatics, Geneva University, Switzerland. 

PMID: 22874339  [PubMed - in process]

Click here for link to PubMed for article 22874339
```

----------------------------

----------------------------

```
Abstract 20 of 362

1. Stud Health Technol Inform. 2012;180:958-62.

New approaches to linking clinical guidelines to virtual patients.

Kononowicz AA, Hege I, Krawczyk P, Zary N.

Jagiellonian University Medical College, Kraków, Poland.

PMID: 22874335  [PubMed - in process]

Click here for link to PubMed for article 22874335
```

----------------------------

----------------------------

```
Abstract 21 of 362

1. Stud Health Technol Inform. 2012;180:954-7.

Criteria to assess the quality of virtual patients.

Hege I, Zary N, Kononowicz AA.

Department for Medical Education, LMU Munich, Germany.

PMID: 22874334  [PubMed - in process]

Click here for link to PubMed for article 22874334
```

----------------------------

----------------------------

```
Abstract 22 of 362

1. BMC Med Educ. 2012 Aug 1;12:62. doi: 10.1186/1472-6920-12-62.

Virtual patients design and its effect on clinical reasoning and student
experience: a protocol for a randomised factorial multi-centre study.

Bateman J, Allen ME, Kidd J, Parsons N, Davies D.

Education and Development Research Team, Warwick Medical School, Coventry CV4
7AL, UK.

PMCID: PMC3441814
PMID: 22853706  [PubMed - indexed for MEDLINE]

Click here for link to PubMed for article 22853706
```

----------------------------

----------------------------

```
Abstract 23 of 362

1. Bull Acad Natl Med. 2011 Nov;195(8):1913-20; discussion 1920-1.

[Simulation in pediatric surgery].

[Article in French]

Becmeur F, Lacreuse I, Soler L.

Chirurgie pédiatrique, Hôpital de Hautepierre, 67098 Strasbourg cédex.

PMID: 22844751  [PubMed - indexed for MEDLINE]

Click here for link to PubMed for article 22844751
```

----------------------------

----------------------------

```
Abstract 24 of 362

1. Med Teach. 2012;34(8):674-5. doi: 10.3109/0142159X.2012.689450.

Using virtual patients to teach medical ethics, medical law and medical
professionalism.

Hooper CR, Jivram T, Law S, Michell A, Somasunderam A.

PMID: 22830327  [PubMed - indexed for MEDLINE]

Click here for link to PubMed for article 22830327
```

----------------------------

----------------------------

```
Abstract 25 of 362

1. Clin Teach. 2012 Aug;9(4):216-21. doi: 10.1111/j.1743-498X.2012.00551.x.

The virtual continuity in learning programme: results.

Wood E, Tso S.

Academic Department of Medical and Surgical Gastroenterology, Homerton University
Hospital NHS Foundation Trust, London, UK.

© Blackwell Publishing Ltd 2012.

PMID: 22783852  [PubMed - indexed for MEDLINE]

Click here for link to PubMed for article 22783852
```

----------------------------

----------------------------

```
Abstract 26 of 362

1. PLoS Comput Biol. 2012;8(6):e1002571. doi: 10.1371/journal.pcbi.1002571. Epub
2012 Jun 28.

Virtual patients and sensitivity analysis of the Guyton model of blood pressure
regulation: towards individualized models of whole-body physiology.

Moss R, Grosse T, Marchant I, Lassau N, Gueyffier F, Thomas SR.

IR4M UMR8081 CNRS, Université Paris-Sud, Orsay, France.

PMCID: PMC3386164
PMID: 22761561  [PubMed - indexed for MEDLINE]

Click here for link to PubMed for article 22761561
```

----------------------------

----------------------------

```
Abstract 27 of 362

1. Am J Pharm Educ. 2012 Jun 18;76(5):92. doi: 10.5688/ajpe76592.

Virtual patients in pharmacy education.

Jabbur-Lopes MO, Mesquita AR, Silva LM, De Almeida Neto A, Lyra DP Jr.

College of Pharmacy, Federal University of Sergipe, Brazil

PMCID: PMC3386043
PMID: 22761533  [PubMed - indexed for MEDLINE]

Click here for link to PubMed for article 22761533
```

----------------------------

----------------------------

```
Abstract 28 of 362

1. BMC Med Educ. 2012 Jun 18;12:41. doi: 10.1186/1472-6920-12-41.

Effects of introducing a voluntary virtual patient module to a basic life support
with an automated external defibrillator course: a randomised trial.

Kononowicz AA, Krawczyk P, Cebula G, Dembkowska M, Drab E, Frączek B, Stachoń AJ,
Andres J.

Department of Bioinformatics and Telemedicine, Jagiellonian University Medical
College, Lazarza 16, Krakow 31-530, Poland.

PMCID: PMC3408380
PMID: 22709278  [PubMed - indexed for MEDLINE]

Click here for link to PubMed for article 22709278
```

----------------------------

----------------------------

```
Abstract 29 of 362

1. J Surg Educ. 2012 Jul;69(4):449-52. doi: 10.1016/j.jsurg.2012.05.013.

Novel educational approach for medical students: improved retention rates using
interactive medical software compared with traditional lecture-based format.

Subramanian A, Timberlake M, Mittakanti H, Lara M, Brandt ML.

Michael E. DeBakey Department of Surgery, Baylor College of Medicine, Houston, TX
77030, USA.

Copyright © 2012 Association of Program Directors in Surgery. Published by
Elsevier Inc. All rights reserved.

PMID: 22677580  [PubMed - indexed for MEDLINE]

Click here for link to PubMed for article 22677580
```

----------------------------

----------------------------

```
Abstract 30 of 362

1. Clin Teach. 2012 Jun;9(3):152-7. doi: 10.1111/j.1743-498X.2011.00523.x.

evPaeds: undergraduate clinical reasoning.

Pinnock R, Spence F, Chung A, Booth R.

Department of Paediatrics: Child and Youth Health, University of Auckland,
Auckland, New Zealand.

© Blackwell Publishing Ltd 2012.

PMID: 22587313  [PubMed - indexed for MEDLINE]

Click here for link to PubMed for article 22587313
```

----------------------------

----------------------------

```
Abstract 31 of 362

1. Vasc Health Risk Manag. 2012;8:255-64. doi: 10.2147/VHRM.S28744. Epub 2012 Apr
23.

Health and economic outcomes for exenatide once weekly, insulin, and pioglitazone
therapies in the treatment of type 2 diabetes: a simulation analysis.

Gaebler JA, Soto-Campos G, Alperin P, Cohen M, Blickensderfer A, Wintle M, Maggs 
D, Hoogwerf B, Han J, Pencek R, Peskin B.

Amylin Pharmaceuticals, Inc, San Diego, CA, USA.

PMCID: PMC3346268
PMID: 22566747  [PubMed - indexed for MEDLINE]

Click here for link to PubMed for article 22566747
```

----------------------------

----------------------------

```
Abstract 32 of 362

1. J Diabetes Sci Technol. 2012 Mar 1;6(2):371-9.

The identifiable virtual patient model: comparison of simulation and clinical
closed-loop study results.

Kanderian SS, Weinzimer SA, Steil GM.

Yale University, New Haven, Connecticut, USA.

© 2012 Diabetes Technology Society.

PMCID: PMC3380781 [Available on 2013/3/1]
PMID: 22538149  [PubMed - indexed for MEDLINE]

Click here for link to PubMed for article 22538149
```

----------------------------

----------------------------

```
Abstract 33 of 362

1. Acad Psychiatry. 2012 Mar 1;36(2):146-50. doi: 10.1176/appi.ap.10030049.

Interactive virtual-patient scenarios: an evolving tool in psychiatric education.

Shah H, Rossen B, Lok B, Londino D, Lind SD, Foster A.

Georgia Health Sciences University, USA.

PMID: 22532209  [PubMed - indexed for MEDLINE]

Click here for link to PubMed for article 22532209
```

----------------------------

----------------------------

```
Abstract 34 of 362

1. Med Teach. 2012;34(4):e222-8. doi: 10.3109/0142159X.2012.642830.

Virtual patients for assessment of medical student ability to integrate clinical 
and laboratory data to develop differential diagnoses: comparison of results of
exams with/without time constraints.

Gunning WT, Fors UG.

University of Toledo, USA.

PMID: 22455713  [PubMed - indexed for MEDLINE]

Click here for link to PubMed for article 22455713
```

----------------------------

----------------------------

```
Abstract 35 of 362

1. Med Educ. 2012 Apr;46(4):417-25. doi: 10.1111/j.1365-2923.2012.04219.x.

Integrating virtual patients into courses: follow-up seminars and perceived
benefit.

Edelbring S, Broström O, Henriksson P, Vassiliou D, Spaak J, Dahlgren LO, Fors U,
Zary N.

Department of Learning, Informatics, Management and Ethics, Karolinska
Institutet, Berzelius Väg 3, Stockholm, Sweden.

© Blackwell Publishing Ltd 2012.

PMID: 22429178  [PubMed - indexed for MEDLINE]

Click here for link to PubMed for article 22429178
```

----------------------------

----------------------------

```
Abstract 36 of 362

1. Comput Methods Programs Biomed. 2012 Mar 17. [Epub ahead of print]

Adaptive blood glucose control for intensive care applications.

Ottavian M, Barolo M, Zisser H, Dassau E, Seborg DE.

Dipartimento di Principi e Impianti di Ingegneria Chimica, Universit�  di Padova, 
via Marzolo 9, 35131 Padova PD, Italy.

Copyright Â© 2012 Elsevier Ireland Ltd. All rights reserved.

PMID: 22424730  [PubMed - as supplied by publisher]

Click here for link to PubMed for article 22424730
```

----------------------------

----------------------------

```
Abstract 37 of 362

1. Clin Teach. 2012 Apr;9(2):133-4. doi: 10.1111/j.1743-498X.2012.00546.x.

Virtual patients can be used to teach clinical reasoning.

Bateman J, Hariman C, Nassrally M.

Comment on
    Clin Teach. 2011 Sep;8(3):176-80.

PMID: 22405376  [PubMed - indexed for MEDLINE]

Click here for link to PubMed for article 22405376
```

----------------------------

----------------------------

```
Abstract 38 of 362

1. Biochem Med (Zagreb). 2012;22(1):86-91.

Validation of a laboratory and hospital information system in a medical
laboratory accredited according to ISO 15189.

Biljak VR, Ozvald I, Radeljak A, Majdenic K, Lasic B, Siftar Z, Lovrencic MV,
Flegar-Mestric Z.

Merkur University Hospital, Institute of Clinical Chemistry and Laboratory
Medicine, Zagreb, Croatia.

PMID: 22384522  [PubMed - indexed for MEDLINE]

Click here for link to PubMed for article 22384522
```

----------------------------

----------------------------

```
Abstract 39 of 362

1. Adv Physiol Educ. 2012 Mar;36(1):48-53. doi: 10.1152/advan.00054.2011.

The use of virtual patients in medical school curricula.

Cendan J, Lok B.

Department of Medical Education, College of Medicine, University of Central
Florida, Orlando, USA.

PMID: 22383412  [PubMed - indexed for MEDLINE]

Click here for link to PubMed for article 22383412
```

----------------------------

----------------------------

```
Abstract 40 of 362

1. J Surg Educ. 2012 Mar-Apr;69(2):253-6. doi: 10.1016/j.jsurg.2011.12.007.

Novel educational approach for medical students: improved retention rates using
interactive medical software compared with traditional lecture-based format.

Subramanian A, Timberlake M, Mittakanti H, Lara M, Brandt ML.

Michael E. DeBakey Department of Surgery, Baylor College of Medicine, Houston, TX
77030, USA.

Erratum in
    J Surg Educ. 2012 May-Jun;69(3):443.

Copyright Â© 2012 Association of Program Directors in Surgery. Published by
Elsevier Inc. All rights reserved.

PMID: 22365876  [PubMed - indexed for MEDLINE]

Click here for link to PubMed for article 22365876
```

----------------------------

----------------------------

```
Abstract 41 of 362

1. Stud Health Technol Inform. 2012;173:372-8.

Shader Lamps Virtual Patients: the physical manifestation of virtual patients.

Rivera-Gutierrez D, Welch G, Lincoln P, Whitton M, Cendan J, Chesnutt DA, Fuchs
H, Lok B.

Department of Computer & Information Science & Engineering, University of
Florida, USA.

PMID: 22357021  [PubMed - indexed for MEDLINE]

Click here for link to PubMed for article 22357021
```

----------------------------

----------------------------

```
Abstract 42 of 362

1. PLoS Comput Biol. 2012 Feb;8(2):e1002355. doi: 10.1371/journal.pcbi.1002355. Epub
2012 Feb 2.

In silico experimentation of glioma microenvironment development and anti-tumor
therapy.

Wu Y, Lu Y, Chen W, Fu J, Fan R.

Department of Biomedical Engineering, Yale University, New Haven, Connecticut,
USA.

PMCID: PMC3271023
PMID: 22319429  [PubMed - indexed for MEDLINE]

Click here for link to PubMed for article 22319429
```

----------------------------

----------------------------

```
Abstract 43 of 362

1. Front Health Serv Manage. 2011 Winter;28(2):3-14.

Social media: ubiquitous community and patient engagement.

Thielst CB.

Comment in
    Front Health Serv Manage. 2011 Winter;28(2):23-7.
    Front Health Serv Manage. 2011 Winter;28(2):29-33.
    Front Health Serv Manage. 2011 Winter;28(2):35-40.

PMID: 22256506  [PubMed - indexed for MEDLINE]

Click here for link to PubMed for article 22256506
```

----------------------------

----------------------------

```
Abstract 44 of 362

1. Conf Proc IEEE Eng Med Biol Soc. 2011;2011:4538-41. doi:
10.1109/IEMBS.2011.6091124.

Pilot study on effectiveness of simulation for surgical robot design using
manipulability.

Kawamura K, Seno H, Kobayashi Y, Fujie MG.

Faculty of Science and Engineering, Waseda University, Tokyo, Japan.

PMID: 22255347  [PubMed - indexed for MEDLINE]

Click here for link to PubMed for article 22255347
```

----------------------------

----------------------------

```
Abstract 45 of 362

1. Med Teach. 2012;34(1):e15-20. doi: 10.3109/0142159X.2012.638010.

New directions in e-learning research in health professions education: Report of 
two symposia.

Triola MM, Huwendiek S, Levinson AJ, Cook DA.

New York University School of Medicine, New York, NY, USA.

PMID: 22250691  [PubMed - indexed for MEDLINE]

Click here for link to PubMed for article 22250691
```

----------------------------

----------------------------

```
Abstract 46 of 362

1. Am J Med Genet A. 2012 Feb;158A(2):384-90. doi: 10.1002/ajmg.a.34422. Epub 2012
Jan 13.

Effectively training pediatric residents to deliver diagnoses of Down syndrome.

Lunney CA, Kleinert HL, Ferguson JE 2nd, Campbell L.

The Human Development Institute, University of Kentucky, Lexington, Kentucky,
USA.

Copyright © 2012 Wiley Periodicals, Inc.

PMID: 22246816  [PubMed - indexed for MEDLINE]

Click here for link to PubMed for article 22246816
```

----------------------------

----------------------------

```
Abstract 47 of 362

1. Simul Healthc. 2012 Feb;7(1):10-7. doi: 10.1097/SIH.0b013e31823652de.

Can a virtual patient trainer teach student nurses how to save lives--teaching
nursing students about pediatric respiratory diseases.

LeFlore JL, Anderson M, Zielke MA, Nelson KA, Thomas PE, Hardee G, John LD.

Pediatric, Acute Care Pediatric & Neonatal Nurse Practitioner Program, The
University of Texas at Arlington, College of Nursing, Arlington, Texas, USA.

PMID: 22228285  [PubMed - indexed for MEDLINE]

Click here for link to PubMed for article 22228285
```

----------------------------

----------------------------

```
Abstract 48 of 362

1. Clin Teach. 2012 Feb;9(1):32-6. doi: 10.1111/j.1743-498X.2011.00487.x.

Teaching professionalism through virtual means.

McEvoy M, Butler B, MacCarrick G.

The Royal College of Surgeons, Dublin, Ireland.

© Blackwell Publishing Ltd 2012.

PMID: 22225890  [PubMed - indexed for MEDLINE]

Click here for link to PubMed for article 22225890
```

----------------------------

----------------------------

```
Abstract 49 of 362

1. J Behav Health Serv Res. 2012 Apr;39(2):103-15. doi: 10.1007/s11414-011-9268-5.

Predictors of primary care physicians' self-reported intention to conduct suicide
risk assessments.

Hooper LM, Epstein SA, Weinfurt KP, DeCoster J, Qu L, Hannah NJ.

Department of Educational Studies in Psychology, Research Methodology, and
Counseling, The University of Alabama, Box 870231; 315-B Graves Hall, Tuscaloosa,
AL 35487, USA.

PMID: 22218814  [PubMed - indexed for MEDLINE]

Click here for link to PubMed for article 22218814
```

----------------------------

----------------------------

```
Abstract 50 of 362

1. AMIA Annu Symp Proc. 2011;2011:1355-60. Epub 2011 Oct 22.

Family physicians' completion of scoring criteria in Virtual Patient encounters.

Sumner W 2nd, O'Neill TR, Roussel G, Xu JZ, Fu H, Ivins D, Hagen MD.

Washington University, St. Louis, MO, USA.

PMCID: PMC3243160
PMID: 22195197  [PubMed - in process]

Click here for link to PubMed for article 22195197
```

----------------------------

----------------------------

```
Abstract 51 of 362

1. AMIA Annu Symp Proc. 2011;2011:905-14. Epub 2011 Oct 22.

A cognitive architecture for simulating bodies and minds.

Nirenburg S, McShane M, Beale S, Catizone R.

University of Maryland Baltimore County, MD, USA.

PMCID: PMC3243225
PMID: 22195149  [PubMed - in process]

Click here for link to PubMed for article 22195149
```

----------------------------

----------------------------

```
Abstract 52 of 362

1. J Cogn Neurosci. 2012 Apr;24(4):809-18. doi: 10.1162/jocn_a_00179. Epub 2011 Dec 
20.

Extinguishing extinction: hemispheric differences in the modulation of
TMS-induced visual extinction by directing covert spatial attention.

Bien N, Goebel R, Sack AT.

Department of Cognitive Neuroscience, Faculty of Psychology and Neuroscience,
Maastricht University, P.O. Box 616, 6200 MD Maastricht, the Netherlands.

PMID: 22185491  [PubMed - indexed for MEDLINE]

Click here for link to PubMed for article 22185491
```

----------------------------

----------------------------

```
Abstract 53 of 362

1. PLoS One. 2011;6(11):e27768. doi: 10.1371/journal.pone.0027768. Epub 2011 Nov 23.

Neural correlates of effective learning in experienced medical decision-makers.

Downar J, Bhatt M, Montague PR.

Department of Psychiatry, University of Toronto and Toronto Western Hospital,
Toronto, Ontario, Canada.

PMCID: PMC3223201
PMID: 22132137  [PubMed - indexed for MEDLINE]

Click here for link to PubMed for article 22132137
```

----------------------------

----------------------------

```
Abstract 54 of 362

1. Ir Med J. 2011 Sep;104(8):250-2.

Virtual patients: an effective educational intervention to improve paediatric
basic specialist trainee education in the management of suspected child abuse?

McEvoy MM, Butler B, MacCarrick G, Nicholson AJ.

The Children's University Hospital, Temple Street, Dublin 1.

PMID: 22125882  [PubMed - indexed for MEDLINE]

Click here for link to PubMed for article 22125882
```

----------------------------

----------------------------

```
Abstract 55 of 362

1. Int J Clin Pharm. 2012 Feb;34(1):17-22. doi: 10.1007/s11096-011-9584-0. Epub 2011
Nov 23.

Assessment of a computerised decision support system for allergic
rhino-conjunctivitis counselling in German pharmacy.

Bertsche T, Nachbar M, Fiederling J, Schmitt SP, Kaltschmidt J, Seidling HM,
Haefeli WE.

Department of Clinical Pharmacology and Pharmacoepidemiology, University of
Heidelberg, Im Neuenheimer Feld 410, 69120, Heidelberg, Germany.

PMID: 22108789  [PubMed - indexed for MEDLINE]

Click here for link to PubMed for article 22108789
```

----------------------------

----------------------------

```
Abstract 56 of 362

1. Eur J Pharm Sci. 2012 Jan 23;45(1-2):90-100. doi: 10.1016/j.ejps.2011.10.022.
Epub 2011 Nov 10.

Efficacy, nephrotoxicity and ototoxicity of aminoglycosides, mathematically
modelled for modelling-supported therapeutic drug monitoring.

Croes S, Koop AH, van Gils SA, Neef C.

Department of Clinical Pharmacy & Toxicology, Maastricht University Medical
Center, Maastricht, The Netherlands.

Copyright © 2011 Elsevier B.V. All rights reserved.

PMID: 22094306  [PubMed - indexed for MEDLINE]

Click here for link to PubMed for article 22094306
```

----------------------------

----------------------------

```
Abstract 57 of 362

1. J Psychiatr Ment Health Nurs. 2012 Jun;19(5):410-8. doi:
10.1111/j.1365-2850.2011.01797.x. Epub 2011 Nov 1.

What can virtual patient simulation offer mental health nursing education?

Guise V, Chambers M, Välimäki M.

Faculty of Health and Social Care Sciences, St George's University of London &
Kingston University, London, UK.

© 2011 Blackwell Publishing.

PMID: 22070549  [PubMed - indexed for MEDLINE]

Click here for link to PubMed for article 22070549
```

----------------------------

----------------------------

```
Abstract 58 of 362

1. Nurse Educ Today. 2012 Aug;32(6):683-9. doi: 10.1016/j.nedt.2011.09.004. Epub
2011 Nov 4.

Development, implementation and initial evaluation of narrative virtual patients 
for use in vocational mental health nurse training.

Guise V, Chambers M, Conradi E, Kavia S, Välimäki M.

Faculty of Health and Social Care Sciences, St George's University of London &
Kingston University, Cranmer Terrace, London SW17 0RE, UK.

Copyright © 2011 Elsevier Ltd. All rights reserved.

PMID: 22056146  [PubMed - indexed for MEDLINE]

Click here for link to PubMed for article 22056146
```

----------------------------

----------------------------

```
Abstract 59 of 362

1. Med Teach. 2011;33(11):933-7. doi: 10.3109/0142159X.2011.613501.

Virtual patients: a year of change.

Poulton T, Balasubramaniam C.

Centre for Medical and Healthcare Education, St George's University of London,
UK.

PMID: 22022903  [PubMed - indexed for MEDLINE]

Click here for link to PubMed for article 22022903
```

----------------------------

----------------------------

```
Abstract 60 of 362

1. Acad Psychiatry. 2011 Fall;35(5):328-30. doi: 10.1176/appi.ap.35.5.328.

Assessment of competencies by use of virtual patient technology.

Williams K, Wryobeck J, Edinger W, McGrady A, Fors U, Zary N.

Dept. of Psychiatry, University of Toledo, OH, USA.

PMID: 22007093  [PubMed - indexed for MEDLINE]

Click here for link to PubMed for article 22007093
```

----------------------------

----------------------------

```
Abstract 61 of 362

1. Facial Plast Surg Clin North Am. 2011 Nov;19(4):615-22, viii. doi:
10.1016/j.fsc.2011.07.005.

Creation of the virtual patient for the study of facial morphology.

Kau CH.

Department of Orthodontics, University of Alabama at Birmingham, School of
Dentistry, 1919 7th Avenue South, Birmingham, AL 35294, USA.

Copyright © 2011 Elsevier Inc. All rights reserved.

PMID: 22004856  [PubMed - indexed for MEDLINE]

Click here for link to PubMed for article 22004856
```

----------------------------

----------------------------

```
Abstract 62 of 362

1. J Gen Intern Med. 2011 Nov;26 Suppl 2:639-47. doi: 10.1007/s11606-011-1806-6.

A re-conceptualization of access for 21st century healthcare.

Fortney JC, Burgess JF Jr, Bosworth HB, Booth BM, Kaboli PJ.

Health Services Research and Development (HSR&D), Center for Mental Health and
Outcomes Research, Central Arkansas Veterans Healthcare System, 2200 Fort Roots
Drive, North Little Rock, AR 72114, USA.

PMCID: PMC3191218
PMID: 21989616  [PubMed - indexed for MEDLINE]

Click here for link to PubMed for article 21989616
```

----------------------------

----------------------------

```
Abstract 63 of 362

1. BMC Med Educ. 2011 Oct 12;11:82. doi: 10.1186/1472-6920-11-82.

Student perception of two different simulation techniques in oral and
maxillofacial surgery undergraduate training.

Lund B, Fors U, Sejersen R, Sallnäs EL, Rosén A.

Division of Oral and Maxillofacial Surgery, Department of Dental Medicine,
Karolinska Institutet, Stockholm, Sweden.

PMCID: PMC3209444
PMID: 21992604  [PubMed - indexed for MEDLINE]

Click here for link to PubMed for article 21992604
```

----------------------------

----------------------------

```
Abstract 64 of 362

1. Acad Emerg Med. 2011 Oct;18(10):1094-8. doi: 10.1111/j.1553-2712.2011.01166.x.
Epub 2011 Sep 26.

Theoretical analysis of the effect of positioning on hemodynamic stability during
pregnancy.

Summers RL, Harrison JM, Thompson JR, Porter J, Coleman TG.

Department of Emergency Medicine, University of Mississippi Medical Center,
Jackson, USA.

© 2011 by the Society for Academic Emergency Medicine.

PMID: 21951760  [PubMed - indexed for MEDLINE]

Click here for link to PubMed for article 21951760
```

----------------------------

----------------------------

```
Abstract 65 of 362

1. Med Teach. 2011;33(10):e522-7. doi: 10.3109/0142159X.2011.599889.

A virtual surgery in general practice: evaluation of a novel undergraduate
virtual patient learning package.

Gormley GJ, McGlade K, Thomson C, McGill M, Sun J.

Queen's University Belfast, UK.

PMID: 21942488  [PubMed - indexed for MEDLINE]

Click here for link to PubMed for article 21942488
```

----------------------------

----------------------------

```
Abstract 66 of 362

1. Comput Methods Programs Biomed. 2011 Sep 20. [Epub ahead of print]

Impact of variation in patient response on model-based control of glycaemia in
critically ill patients.

Le Compte AJ, Pretty CG, Lin J, Shaw GM, Lynn A, Chase JG.

Department of Mechanical Engineering, University of Canterbury, Christchurch, New
Zealand.

Copyright © 2011 Elsevier Ireland Ltd. All rights reserved.

PMID: 21940063  [PubMed - as supplied by publisher]

Click here for link to PubMed for article 21940063
```

----------------------------

----------------------------

```
Abstract 67 of 362

1. Clin Pharmacokinet. 2011 Oct;50(10):675-86. doi:
10.2165/11595320-000000000-00000.

Rivaroxaban: population pharmacokinetic analyses in patients treated for acute
deep-vein thrombosis and exposure simulations in patients with atrial
fibrillation treated for stroke prevention.

Mueck W, Lensing AW, Agnelli G, Decousus H, Prandoni P, Misselwitz F.

Bayer HealthCare, Wuppertal, Germany.

Erratum in
    Clin Pharmacokinet. 2012 Feb;51(2):136.

PMID: 21895039  [PubMed - indexed for MEDLINE]

Click here for link to PubMed for article 21895039
```

----------------------------

----------------------------

```
Abstract 68 of 362

1. Stud Health Technol Inform. 2011;169:233-7.

Implementation of a web-based interactive virtual patient case simulation as a
training and assessment tool for medical students.

Oliven A, Nave R, Gilad D, Barch A.

Faculty of Medicine, Technion, Haifa, Israel.

PMID: 21893748  [PubMed - indexed for MEDLINE]

Click here for link to PubMed for article 21893748
```

----------------------------

----------------------------

```
Abstract 69 of 362

1. Stud Health Technol Inform. 2011;169:203-7.

Push and pull models to manage patient consent and licensing of multimedia
resources in digital repositories for case-based reasoning.

Kononowicz AA, Zary N, Davies D, Heid J, Woodham L, Hege I.

Jagiellonian University Medical College, Kraków, Poland.

PMID: 21893742  [PubMed - indexed for MEDLINE]

Click here for link to PubMed for article 21893742
```

----------------------------

----------------------------

```
Abstract 70 of 362

1. GMS Z Med Ausbild. 2011;28(3):Doc42. doi: 10.3205/zma000754. Epub 2011 Aug 8.

Acceptance of medical training cases as supplement to lectures.

Hörnlein A, Mandel A, Ifland M, Lüneberg E, Deckert J, Puppe F.

Universität Würzburg, Fakultät für Mathematik und Informatik, Lehrstuhl für
Künstliche Intelligenz und Angewandte Informatik, Würzburg, Deutschland.

PMCID: PMC3159197
PMID: 21866244  [PubMed]

Click here for link to PubMed for article 21866244
```

----------------------------

----------------------------

```
Abstract 71 of 362

1. IEEE Trans Biomed Eng. 2012 Jan;59(1):54-7. doi: 10.1109/TBME.2011.2163310. Epub 
2011 Jul 29.

In silico evaluation of glucose control protocols for critically ill patients.

Lee JC, Kim M, Choi KR, Oh TJ, Kim MY, Cho YM, Kim K, Kim HC, Kim S.

Institute of Medical and Biological Engineering, Seoul National University, Seoul
110-799, Korea.

© 2011 IEEE

PMID: 21803673  [PubMed - indexed for MEDLINE]

Click here for link to PubMed for article 21803673
```

----------------------------

----------------------------

```
Abstract 72 of 362

1. Med Phys. 2011 May;38(5):2639-50.

Real time 4D IMRT treatment planning based on a dynamic virtual patient model:
proof of concept.

Guo B, Xu XG, Shi C.

Radiation Oncology, University of Michigan, Ann Arbor, Michigan 48109, USA.

PMCID: PMC3107830
PMID: 21776801  [PubMed - indexed for MEDLINE]

Click here for link to PubMed for article 21776801
```

----------------------------

----------------------------

```
Abstract 73 of 362

1. Stud Health Technol Inform. 2011;167:165-9.

Simulated interviews 3.0: virtual humans to train abilities of
diagnosis--usability assessment.

Peñaloza-Salazar C, Gutierrez-Maldonado J, Ferrer-Garcia M, Garcia-Palacios A,
Andres-Pueyo A, Aguilar-Alonso A.

University of Barcelona, Spain.

PMID: 21685661  [PubMed - indexed for MEDLINE]

Click here for link to PubMed for article 21685661
```

----------------------------

----------------------------

```
Abstract 74 of 362

1. Eur J Vasc Endovasc Surg. 2011 Oct;42(4):539-48. doi: 10.1016/j.ejvs.2011.05.003.
Epub 2011 Jun 15.

Construct validity and reliability of structured assessment of endoVascular
expertise in a simulated setting.

Bech B, Lönn L, Falkenberg M, Bartholdy NJ, Räder SB, Schroeder TV, Ringsted C.

Centre for Clinical Education, University of Copenhagen and Capital Region of
Denmark, Denmark.

Copyright © 2011 European Society for Vascular Surgery. Published by Elsevier
Ltd. All rights reserved.

PMID: 21680207  [PubMed - indexed for MEDLINE]

Click here for link to PubMed for article 21680207
```

----------------------------

----------------------------

```
Abstract 75 of 362

1. Adv Health Sci Educ Theory Pract. 2011 Aug;16(3):331-45. doi:
10.1007/s10459-010-9265-0. Epub 2011 Jun 9.

Experiencing virtual patients in clinical learning: a phenomenological study.

Edelbring S, Dastmalchi M, Hult H, Lundberg IE, Dahlgren LO.

Department of Learning, Informatics, Management and Ethics, Karolinska
Institutet, Stockholm, Sweden.

PMID: 21656337  [PubMed - indexed for MEDLINE]

Click here for link to PubMed for article 21656337
```

----------------------------

----------------------------

```
Abstract 76 of 362

1. Clin Oral Implants Res. 2012 Feb;23(2):257-60. doi:
10.1111/j.1600-0501.2011.02221.x. Epub 2011 Jun 2.

EAO summer camp: a facilitated sharing experience.

EAO Junior Committee members, Jung RE, Kapos T, Nicol A, Nisand D, Palarie V,
Payer M, Rocchietta I, Schwarz F.

Clinic for Fixed and Removable Prosthodontics and Dental Material Science,
University of Zurich, Zurich, Switzerland.

© 2011 John Wiley & Sons A/S.

PMID: 21635558  [PubMed - indexed for MEDLINE]

Click here for link to PubMed for article 21635558
```

----------------------------

----------------------------

```
Abstract 77 of 362

1. Yonsei Med J. 2011 Jul;52(4):616-23. doi: 10.3349/ymj.2011.52.4.616.

Teicoplanin dosing strategy for treatment of Staphylococcus aureus in Korean
patients with neutropenic fever.

Ahn BJ, Yim DS, Lee DG, Kwon JC, Kim SH, Choi SM.

Department of Internal Medicine, College of Medicine, The Catholic University of 
Korea, 505 Banpo-dong, Seocho-gu, Seoul 137-701, Korea.

PMCID: PMC3104459
PMID: 21623604  [PubMed - indexed for MEDLINE]

Click here for link to PubMed for article 21623604
```

----------------------------

----------------------------

```
Abstract 78 of 362

1. Cogn Neuropsychiatry. 2012;17(1):36-63. doi: 10.1080/13546805.2011.564925. Epub
2011 Jun 28.

"That's not my arm": a hypnotic analogue of somatoparaphrenia.

Rahmanovic A, Barnier AJ, Cox RE, Langdon RA, Coltheart M.

Macquarie Centre for Cognitive Science (MACCS), Macquarie University, Sydney,
Australia.

PMID: 21623487  [PubMed - indexed for MEDLINE]

Click here for link to PubMed for article 21623487
```

----------------------------

----------------------------

```
Abstract 79 of 362

1. Med Humanit. 2011 Jun;37(1):9-12. doi: 10.1136/jmh.2010.005637. Epub 2011 Feb 17.

Black, white or green: 'race', gender and avatars within the therapeutic space.

Graber MA, Graber AD.

Department of Emergency Medicine, University of Iowa Carver College of Medicine, 
Iowa 52242, USA.

PMID: 21593244  [PubMed - indexed for MEDLINE]

Click here for link to PubMed for article 21593244
```

----------------------------

----------------------------

```
Abstract 80 of 362

1. Chirurg. 2012 Jan;83(1):45-53. doi: 10.1007/s00104-011-2102-z.

[Implementation of the eLearning project NESTOR. A network for students in
traumatology and orthopedics].

[Article in German]

Back DA, Haberstroh N, Hoff E, Plener J, Haas NP, Perka C, Schmidmaier G.

Abteilung für Orthopädie und Unfallchirurgie, Bundeswehrkrankenhaus Berlin,
Scharnhorststr. 13, 10115, Berlin, Deutschland.

PMID: 21559928  [PubMed - indexed for MEDLINE]

Click here for link to PubMed for article 21559928
```

----------------------------

----------------------------

```
Abstract 81 of 362

1. Front Physiol. 2011 Feb 24;2:4. doi: 10.3389/fphys.2011.00004.

A computational systems biology software platform for multiscale modeling and
simulation: integrating whole-body physiology, disease biology, and molecular
reaction networks.

Eissing T, Kuepfer L, Becker C, Block M, Coboeken K, Gaub T, Goerlitz L, Jaeger
J, Loosen R, Ludewig B, Meyer M, Niederalt C, Sevestre M, Siegmund HU, Solodenko 
J, Thelen K, Telle U, Weiss W, Wendl T, Willmann S, Lippert J.

Competence Center Systems Biology and Computational Solutions, Bayer Technology
Services GmbH Leverkusen, Germany.

PMCID: PMC3070480
PMID: 21483730  [PubMed]

Click here for link to PubMed for article 21483730
```

----------------------------

----------------------------

```
Abstract 82 of 362

1. Med Teach. 2011;33(4):319-24. doi: 10.3109/0142159X.2011.540268.

A collaborative model for developing and maintaining virtual patients for medical
education.

Berman NB, Fall LH, Chessman AW, Dell MR, Lang VJ, Leong SL, Nixon LJ, Smith S.

Dartmouth Medical School, Lebanon, NH, USA.

PMID: 21456990  [PubMed - indexed for MEDLINE]

Click here for link to PubMed for article 21456990
```

----------------------------

----------------------------

```
Abstract 83 of 362

1. Med Teach. 2011;33(4):303-10. doi: 10.3109/0142159X.2011.550969.

Design for learning: deconstructing virtual patient activities.

Ellaway RH, Davies D.

Northern Ontario School of Medicine, Canada.

PMID: 21456988  [PubMed - indexed for MEDLINE]

Click here for link to PubMed for article 21456988
```

----------------------------

----------------------------

```
Abstract 84 of 362

1. Med Teach. 2011;33(4):273-8. doi: 10.3109/0142159X.2011.544796.

How we created virtual patient cases for primary care-based learning.

Adams EC, Rodgers CJ, Harrington R, Young MD, Sieber VK.

John Radcliffe Hospital, UK.

PMID: 21456983  [PubMed - indexed for MEDLINE]

Click here for link to PubMed for article 21456983
```

----------------------------

----------------------------

```
Abstract 85 of 362

1. Br J Clin Pharmacol. 2011 Apr;71(4):536-43. doi:
10.1111/j.1365-2125.2010.03883.x.

In silico prediction of efavirenz and rifampicin drug-drug interaction
considering weight and CYP2B6 phenotype.

Rekić D, Röshammar D, Mukonzo J, Ashton M.

Unit for Pharmacokinetics and Drug Metabolism, Department of Pharmacology,
Sahlgrenska Academy at University of Gothenburg, Gothenburg, Sweden.

© 2011 The Authors. British Journal of Clinical Pharmacology © 2011 The British
Pharmacological Society.

PMCID: PMC3080641
PMID: 21395646  [PubMed - indexed for MEDLINE]

Click here for link to PubMed for article 21395646
```

----------------------------

----------------------------

```
Abstract 86 of 362

1. Int J Occup Environ Health. 2011 Jan-Mar;17(1):63-70.

Implementation of virtual patients in the training for occupational health in
Latin America.

Radon K, Carvalho D, Calvo MJ, Struempell S, Herrera V, Wengenroth L, Kausel G,
Marchetti N, Rojas DS, Russ P, Hege I.

University Hospital of Munich (LMU), Ziemssenstr 180336 Munich, Germany.

PMID: 21344821  [PubMed - indexed for MEDLINE]

Click here for link to PubMed for article 21344821
```

----------------------------

----------------------------

```
Abstract 87 of 362

1. Stud Health Technol Inform. 2011;163:650-2.

Single and multi-user virtual patient design in the virtual world.

Taylor D, Patel V, Cohen D, Aggarwal R, Kerr K, Sevdalis N, Batrick N, Darzi A.

Division of Surgery, Department of Surgery and Cancer, Imperial College London,
St Mary's Hospital, London, UK.

PMID: 21335873  [PubMed - indexed for MEDLINE]

Click here for link to PubMed for article 21335873
```

----------------------------

----------------------------

```
Abstract 88 of 362

1. Stud Health Technol Inform. 2011;163:447-53.

Patient-specific cases for an ultrasound training simulator.

Petrinec K, Savitsky E, Hein C.

Center for Advanced Surgical and Interventional Technology (CASIT), UCLA Computer
Science Department, Los Angeles, CA 90095, USA.

PMID: 21335837  [PubMed - indexed for MEDLINE]

Click here for link to PubMed for article 21335837
```

----------------------------

----------------------------

```
Abstract 89 of 362

1. Stud Health Technol Inform. 2011;163:440-6.

Implementation of virtual online patient simulation.

Patel V, Aggarwal R, Taylor D, Darzi A.

Division of Surgery, Department of Surgery and Cancer, Imperial College London,
St Mary's Hospital, London, UK.

PMID: 21335836  [PubMed - indexed for MEDLINE]

Click here for link to PubMed for article 21335836
```

----------------------------

----------------------------

```
Abstract 90 of 362

1. Stud Health Technol Inform. 2011;163:173-9.

CliniSpace: a multiperson 3D online immersive training environment accessible
through a browser.

Dev P, Heinrichs WL, Youngblood P.

Innovation in Learning Inc, Los Altos Hills, CA, USA.

PMID: 21335784  [PubMed - indexed for MEDLINE]

Click here for link to PubMed for article 21335784
```

----------------------------

----------------------------

```
Abstract 91 of 362

1. Stud Health Technol Inform. 2011;163:144-6.

Mixed virtual reality simulation--taking endoscopic simulation one step further.

Courteille O, Felländer-Tsai L, Hedman L, Kjellin A, Enochsson L, Lindgren G,
Fors U.

Department LIME, Karolinska Institutet, Stockholm, Sweden.

PMID: 21335778  [PubMed - indexed for MEDLINE]

Click here for link to PubMed for article 21335778
```

----------------------------

----------------------------

```
Abstract 92 of 362

1. J Telemed Telecare. 2011;17(3):146-9. doi: 10.1258/jtt.2010.100614. Epub 2011 Feb
8.

Use of telemedicine for haemodialysis in very remote areas: the Canadian First
Nations.

Sicotte C, Moqadem K, Vasilevsky M, Desrochers J, St-Gelais M.

Department of Health Administration, University of Montreal, PO Box 6128, Station
Downtown, Montreal, Quebec H3C 3J7, Canada.

PMID: 21303935  [PubMed - indexed for MEDLINE]

Click here for link to PubMed for article 21303935
```

----------------------------

----------------------------

```
Abstract 93 of 362

1. J Reprod Med. 2010 Nov-Dec;55(11-12):498-502.

Randomized, controlled trial of a normal pregnancy virtual patient to teach
medical students counseling skills.

Ricciotti HA, Hacker MR, De Flesco LD, Dodge LE, Huang GC.

Department of Obstetrics and Gynecology, Beth Israel Deaconess Medical Center,
330 Brookline Avenue, Boston, MA 02215, USA.

PMID: 21291036  [PubMed - indexed for MEDLINE]

Click here for link to PubMed for article 21291036
```

----------------------------

----------------------------

```
Abstract 94 of 362

1. Acad Med. 2011 Feb;86(2):151; author reply 151. doi:
10.1097/ACM.0b013e3182041db4.

Virtual patients: are we in a new era?

Bateman J, Davies D.

Comment on
    Acad Med. 2010 Oct;85(10):1589-602.

PMID: 21270545  [PubMed - indexed for MEDLINE]

Click here for link to PubMed for article 21270545
```

----------------------------

----------------------------

```
Abstract 95 of 362

1. Comput Methods Programs Biomed. 2011 May;102(2):181-91. doi:
10.1016/j.cmpb.2010.03.010. Epub 2011 Jan 17.

Development of blood glucose control for extremely premature infants.

Le Compte AJ, Chase JG, Lynn A, Hann CE, Shaw GM, Lin J.

Department of Mechanical Engineering, University of Canterbury, New Zealand.

Copyright © 2011. Published by Elsevier Ireland Ltd.

PMID: 21247652  [PubMed - indexed for MEDLINE]

Click here for link to PubMed for article 21247652
```

----------------------------

----------------------------

```
Abstract 96 of 362

1. Am J Pharm Educ. 2010 Oct 11;74(8):143.

Virtual patients and problem-based learning in advanced therapeutics.

Benedict N.

University of Pittsburgh School of Pharmacy, Pittsburgh, Pennsylvania, USA.

PMCID: PMC2987283
PMID: 21179254  [PubMed - indexed for MEDLINE]

Click here for link to PubMed for article 21179254
```

----------------------------

----------------------------

```
Abstract 97 of 362

1. Nurse Educ Today. 2011 Nov;31(8):757-62. doi: 10.1016/j.nedt.2010.11.015. Epub
2010 Dec 14.

Virtual patients for assessment of clinical reasoning in nursing -- a pilot
study.

Forsberg E, Georg C, Ziegert K, Fors U.

School of Social and Health Sciences, Halmstad University, Sweden.

Copyright Â© 2010 Elsevier Ltd. All rights reserved.

PMID: 21159412  [PubMed - indexed for MEDLINE]

Click here for link to PubMed for article 21159412
```

----------------------------

----------------------------

```
Abstract 98 of 362

1. Biomed Eng Online. 2010 Dec 14;9:84. doi: 10.1186/1475-925X-9-84.

Validation of a model-based virtual trials method for tight glycemic control in
intensive care.

Chase JG, Suhaimi F, Penning S, Preiser JC, Le Compte AJ, Lin J, Pretty CG, Shaw 
GM, Moorhead KT, Desaive T.

Dept. of Mechanical Engoneering, Centre for Bio-Engineering, University of
Canterbury, Christchurch, New Zealand.

PMCID: PMC3224899
PMID: 21156053  [PubMed - indexed for MEDLINE]

Click here for link to PubMed for article 21156053
```

----------------------------

----------------------------

```
Abstract 99 of 362

1. Ann Behav Med. 2011 Jun;41(3):363-72. doi: 10.1007/s12160-010-9242-0.

Impact of genetic causal information on medical students' clinical encounters
with an obese virtual patient: health promotion and social stigma.

Persky S, Eccleston CP.

Social and Behavioral Research Branch, National Human Genome Research Institute, 
NIH, Bethesda, MD 20892, USA.

PMCID: PMC3098938
PMID: 21136226  [PubMed - indexed for MEDLINE]

Click here for link to PubMed for article 21136226
```

----------------------------

----------------------------

```
Abstract 100 of 362

1. Clin Teach. 2010 Sep;7(3):202-5. doi: 10.1111/j.1743-498X.2010.00382.x.

Virtual patients: practical advice for clinical authors using Labyrinth.

Begg M.

eLearning Manager, Learning Technology Section, University of Edinburgh, UK.

© Blackwell Publishing Ltd 2010.

PMID: 21134184  [PubMed - indexed for MEDLINE]

Click here for link to PubMed for article 21134184
```

----------------------------

----------------------------

```
Abstract 101 of 362

1. J Diabetes Sci Technol. 2010 Nov 1;4(6):1424-37.

Combining basal-bolus insulin infusion for tight postprandial glucose control: an
in silico evaluation in adults, children, and adolescents.

Revert A, Rossetti P, Calm R, Vehí J, Bondia J.

Instituto Universitario de Automática e Informática Industrial, Universidad
Politécnica de Valencia, Camino de Vera s/n, Valencia, Spain.

© 2010 Diabetes Technology Society.

PMCID: PMC3005053
PMID: 21129338  [PubMed - indexed for MEDLINE]

Click here for link to PubMed for article 21129338
```

----------------------------

----------------------------

```
Abstract 102 of 362

1. BMC Med Educ. 2010 Dec 4;10:91. doi: 10.1186/1472-6920-10-91.

Virtual patient simulation: what do students make of it? A focus group study.

Botezatu M, Hult H, Fors UG.

Department of Learning, Informatics, Management and Ethics, Karolinska
Institutet, Stockholm, Sweden.

PMCID: PMC3014956
PMID: 21129220  [PubMed - indexed for MEDLINE]

Click here for link to PubMed for article 21129220
```

----------------------------

----------------------------

```
Abstract 103 of 362

1. Eur J Clin Pharmacol. 2011 Mar;67(3):261-6. doi: 10.1007/s00228-010-0928-9. Epub 
2010 Nov 16.

CYP2C19-guided design of a proton pump inhibitor dose regimen to avoid the need
for pharmacogenetic individualization in H. pylori eradication.

Ward MB, Foster DJ.

Sansom Institute for Health Research, School of Pharmacy and Medical Sciences,
University of South Australia, Adelaide, South Australia, Australia.

PMID: 21079935  [PubMed - indexed for MEDLINE]

Click here for link to PubMed for article 21079935
```

----------------------------

----------------------------

```
Abstract 104 of 362

1. Inform Prim Care. 2010;18(2):73-7.

The Hayes principles: learning from the national pilot of information technology 
and core generalisable theory in informatics.

de Lusignan S, Krause P.

Division of Population Health Sciences and Education, St George's - University of
London, London, UK.

PMID: 21078229  [PubMed - indexed for MEDLINE]

Click here for link to PubMed for article 21078229
```

----------------------------

----------------------------

```
Abstract 105 of 362

1. Prehosp Disaster Med. 2010 Sep-Oct;25(5):424-32.

Training healthcare personnel for mass-casualty incidents in a virtual emergency 
department: VED II.

Heinrichs WL, Youngblood P, Harter P, Kusumoto L, Dev P.

Stanford University Medical Center, Stanford, CA, USA.

Comment in
    Prehosp Disaster Med. 2010 Sep-Oct;25(5):433-4.

PMID: 21053190  [PubMed - indexed for MEDLINE]

Click here for link to PubMed for article 21053190
```

----------------------------

----------------------------

```
Abstract 106 of 362

1. Med Teach. 2010;32(11):e509-16. doi: 10.3109/0142159X.2010.519066.

As time goes by: Stakeholder opinions on the implementation and use of a virtual 
patient simulation system.

Botezatu M, Hult H, Kassaye Tessma M, Fors UG.

Department of Learning, Informatics, Management and Ethics Karolinska Institutet,
Berzelius väg 3, Stockholm 17177, Sweden.

PMID: 21039093  [PubMed - indexed for MEDLINE]

Click here for link to PubMed for article 21039093
```

----------------------------

----------------------------

```
Abstract 107 of 362

1. Ann N Y Acad Sci. 2010 Oct;1208:133-41. doi: 10.1111/j.1749-6632.2010.05756.x.

House calls revisited: leveraging technology to overcome obstacles to veteran
psychiatric care and improve treatment outcomes.

Olden M, Cukor J, Rizzo AS, Rothbaum B, Difede J.

Department of Psychiatry, Weill Cornell Medical College of Cornell University,
New York, New York 10065, USA.

© 2010 Association for Research in Nervous and Mental Disease.

PMID: 20955335  [PubMed - indexed for MEDLINE]

Click here for link to PubMed for article 20955335
```

----------------------------

----------------------------

```
Abstract 108 of 362

1. Nurse Educ Pract. 2011 May;11(3):192-8. doi: 10.1016/j.nepr.2010.08.008. Epub
2010 Sep 24.

Using a virtual patient activity to teach nurse prescribing.

Hurst HM, Marks-Maran D.

Faculty of Health and Social Care Sciences, Kingston University/St George's
University of London, SFL Building, Kingston Hill Campus, Kingston Hill,
Kingston-upon-Thames, KT2 7LB, United Kingdom.

Copyright © 2010 Elsevier Ltd. All rights reserved.

PMID: 20869319  [PubMed - indexed for MEDLINE]

Click here for link to PubMed for article 20869319
```

----------------------------

----------------------------

```
Abstract 109 of 362

1. J Am Geriatr Soc. 2010 Sep;58(9):1786-7. doi: 10.1111/j.1532-5415.2010.03027.x.

Review: women's health module.

Callahan KE.

Geriatrics and Adult Development, Terence Cardinal Cooke Health Care Center,
Mount Sinai School of Medicine, New York, New York 10029, USA.

© 2010, Copyright the Authors. Journal compilation © 2010, The American
Geriatrics Society.

PMID: 20863338  [PubMed - indexed for MEDLINE]

Click here for link to PubMed for article 20863338
```

----------------------------

----------------------------

```
Abstract 110 of 362

1. Med Teach. 2010;32(10):845-50. doi: 10.3109/01421591003695287.

Virtual patient simulation for learning and assessment: Superior results in
comparison with regular course exams.

Botezatu M, Hult H, Tessma MK, Fors UG.

Karolinska Institutet, Stockholm, Sweden.

PMID: 20854161  [PubMed - indexed for MEDLINE]

Click here for link to PubMed for article 20854161
```

----------------------------

----------------------------

```
Abstract 111 of 362

1. Stud Health Technol Inform. 2010;160(Pt 1):238-41.

Implementation, monitoring and utilization of an integrated Hospital Information 
System--lessons from a case study.

Cruz-Correia RJ.

CINTESIS, Al. Prof. Hernani, 4200 Porto, Portugal.

PMID: 20841685  [PubMed - indexed for MEDLINE]

Click here for link to PubMed for article 20841685
```

----------------------------

----------------------------

```
Abstract 112 of 362

1. Int J Obes (Lond). 2011 May;35(5):728-35. doi: 10.1038/ijo.2010.173. Epub 2010
Sep 7.

Medical student bias and care recommendations for an obese versus non-obese
virtual patient.

Persky S, Eccleston CP.

Social and Behavioral Research Branch, National Human Genome Research Institute, 
Bethesda, MD 20892, USA.

PMCID: PMC3000449
PMID: 20820169  [PubMed - indexed for MEDLINE]

Click here for link to PubMed for article 20820169
```

----------------------------

----------------------------

```
Abstract 113 of 362

1. Cleft Palate Craniofac J. 2011 Nov;48(6):639-45. doi: 10.1597/08-181. Epub 2010
Aug 16.

Histology and function: analyzing the uvular muscle.

Landes CA, Weichert F, Steinbauer T, Walczak L, Hasenfus A, Veith C, Schröder A, 
Fritsch H, Theegarten D, Wagner M.

PMID: 20815710  [PubMed - in process]

Click here for link to PubMed for article 20815710
```

----------------------------

----------------------------

```
Abstract 114 of 362

1. Int J Med Inform. 2010 Oct;79(10):716-21. doi: 10.1016/j.ijmedinf.2010.07.007.
Epub 2010 Aug 21.

Using a web-based orthopaedic clinic in the curricular teaching of a German
university hospital: analysis of learning effect, student usage and reception.

Wünschel M, Leichtle U, Wülker N, Kluba T.

University Hospital Tübingen, Department of Orthopaedics, Hoppe-Seyler-Str. 3,
72076 Tübingen, Germany. 

Copyright © 2010 Elsevier Ireland Ltd. All rights reserved.

PMID: 20732829  [PubMed - indexed for MEDLINE]

Click here for link to PubMed for article 20732829
```

----------------------------

----------------------------

```
Abstract 115 of 362

1. Phys Med Biol. 2010 Sep 21;55(18):5283-97. doi: 10.1088/0031-9155/55/18/003. Epub
2010 Aug 18.

Applications of tissue heterogeneity corrections and biologically effective dose 
volume histograms in assessing the doses for accelerated partial breast
irradiation using an electronic brachytherapy source.

Shi C, Guo B, Cheng CY, Eng T, Papanikolaou N.

Cancer Therapy and Research Center, University of Texas Health Science Center at 
San Antonio, TX 78229, USA.

PMID: 20720283  [PubMed - indexed for MEDLINE]

Click here for link to PubMed for article 20720283
```

----------------------------

----------------------------

```
Abstract 116 of 362

1. Acad Med. 2010 Oct;85(10):1589-602. doi: 10.1097/ACM.0b013e3181edfe13.

Computerized virtual patients in health professions education: a systematic
review and meta-analysis.

Cook DA, Erwin PJ, Triola MM.

Office of Education Research, College of Medicine, Mayo Clinic, Rochester,
Minnesota, USA.

Comment in
    Acad Med. 2011 Feb;86(2):151; author reply 151.

PMID: 20703150  [PubMed - indexed for MEDLINE]

Click here for link to PubMed for article 20703150
```

----------------------------

----------------------------

```
Abstract 117 of 362

1. Clin Pharmacokinet. 2010 Sep;49(9):567-71. doi: 10.2165/11532960-000000000-00000.

Routine plasma anti-Xa monitoring is required for low-molecular-weight heparins.

Al-Sallami HS, Barras MA, Green B, Duffull SB.

School of Pharmacy, University of Otago, Dunedin, New Zealand.

Comment in
    Clin Pharmacokinet. 2010 Nov;49(11):773-4.

PMID: 20690780  [PubMed - indexed for MEDLINE]

Click here for link to PubMed for article 20690780
```

----------------------------

----------------------------

```
Abstract 118 of 362

1. Acad Emerg Med. 2010 Aug;17(8):870-6. doi: 10.1111/j.1553-2712.2010.00728.x.

Virtual reality triage training provides a viable solution for
disaster-preparedness.

Andreatta PB, Maslowski E, Petty S, Shim W, Marsh M, Hall T, Stern S, Frankel J.

Department of Medical Education, University of Michigan Medical School, Ann
Arbor, MI, USA.

PMID: 20670325  [PubMed - indexed for MEDLINE]

Click here for link to PubMed for article 20670325
```

----------------------------

----------------------------

```
Abstract 119 of 362

1. Med Teach. 2010;32(7):562-8. doi: 10.3109/01421590903514630.

Virtual patient simulation: knowledge gain or knowledge loss?

Botezatu M, Hult H, Tessma MK, Fors U.

Karolinska Institutet, Stockholm, Sweden.

PMID: 20653378  [PubMed - indexed for MEDLINE]

Click here for link to PubMed for article 20653378
```

----------------------------

----------------------------

```
Abstract 120 of 362

1. Math Biosci. 2010 Sep;227(1):44-55. doi: 10.1016/j.mbs.2010.06.001. Epub 2010 Jun
23.

A fast generalizable solution method for glucose control algorithms.

Hann CE, Docherty P, Chase JG, Shaw GM.

Department of Mechanical Engineering, University of Canterbury, Christchurch, New
Zealand.

Copyright 2010 Elsevier Inc. All rights reserved.

PMID: 20600161  [PubMed - indexed for MEDLINE]

Click here for link to PubMed for article 20600161
```

----------------------------

----------------------------

```
Abstract 121 of 362

1. Physiol Res. 2010;59(6):897-908. Epub 2010 Jun 9.

Restoration of Guyton´s diagram for regulation of the circulation as a basis for 
quantitative physiological model development.

Kofránek J, Rusz J.

Department of Circuit Theory, Faculty of Electrical Engineering, Czech Technical 
University, Prague, Czech Republic.

PMID: 20533860  [PubMed - indexed for MEDLINE]

Click here for link to PubMed for article 20533860
```

----------------------------

----------------------------

```
Abstract 122 of 362

1. Future Oncol. 2010 Jun;6(6):917-27. doi: 10.2217/fon.10.61.

From the evolution of toxin resistance to virtual clinical trials: the role of
mathematical models in oncology.

Agur Z.

Institute for Medical BioMathematics (IMBM), PO Box 282, Hateena Street, 10 Bene 
Ataroth 60991, Israel.

PMID: 20528230  [PubMed - indexed for MEDLINE]

Click here for link to PubMed for article 20528230
```

----------------------------

----------------------------

```
Abstract 123 of 362

1. Med Educ. 2010 May;44(5):519. doi: 10.1111/j.1365-2923.2010.03665.x.

Virtual patient design and curricular integration evaluation toolkit.

Huwendiek S, de Leng BA.

Department of Paediatrics 1, Children's Hospital Heidelberg, Im Neuenheimer Feld 
153, D-69120 Heidelberg, Germany.

PMID: 20519003  [PubMed - indexed for MEDLINE]

Click here for link to PubMed for article 20519003
```

----------------------------

----------------------------

```
Abstract 124 of 362

1. Gerontol Geriatr Educ. 2010;31(2):163-73. doi: 10.1080/02701961003795813.

Virtual patients in geriatric education.

Tan ZS, Mulhausen PL, Smith SR, Ruiz JG.

Geriatric Research, Education and Clinical Center, VA Boston Healthcare System,
Harvard Medical School, Boston, Massachusetts, USA.

PMID: 20509062  [PubMed - indexed for MEDLINE]

Click here for link to PubMed for article 20509062
```

----------------------------

----------------------------

```
Abstract 125 of 362

1. Med Phys. 2010 Apr;37(4):1714-21.

Implications of tissue magnetic susceptibility-related distortion on the rotating
magnet in an MR-linac design.

Wachowicz K, Stanescu T, Thomas SD, Fallone BG.

Department of Medical Physics, Cross Cancer Institute, 11560 University Avenue,
Edmonton, Alberta T6G 1Z2, Canada.

PMID: 20443492  [PubMed - indexed for MEDLINE]

Click here for link to PubMed for article 20443492
```

----------------------------

----------------------------

```
Abstract 126 of 362

1. Comput Methods Programs Biomed. 2010 Jul;99(1):113-23. doi:
10.1016/j.cmpb.2010.02.010. Epub 2010 Apr 28.

Feedforward-feedback multiple predictive controllers for glucose regulation in
type 1 diabetes.

Abu-Rmileh A, Garcia-Gabin W.

Department of Electrical, Electronics and Control Engineering, University of
Girona, Girona, Spain.

Copyright 2010 Elsevier Ireland Ltd. All rights reserved.

PMID: 20430467  [PubMed - indexed for MEDLINE]

Click here for link to PubMed for article 20430467
```

----------------------------

----------------------------

```
Abstract 127 of 362

1. Diabetes Technol Ther. 2010 May;12(5):353-63. doi: 10.1089/dia.2009.0158.

Enhanced accuracy of continuous glucose monitoring by online extended kalman
filtering.

Facchinetti A, Sparacino G, Cobelli C.

Department of Information Engineering, University of Padova , Padova, Italy .

PMID: 20388045  [PubMed - indexed for MEDLINE]

Click here for link to PubMed for article 20388045
```

----------------------------

----------------------------

```
Abstract 128 of 362

1. Med Educ. 2010 May;44(5):521-2. doi: 10.1111/j.1365-2923.2010.03653.x. Epub 2010 
Mar 30.

Blended learning using virtual patients and skills laboratory training.

Lehmann R, Bosse HM, Huwendiek S.

University Children's Hospital Heidelberg, Im Neuenheimer Feld 153, 69120
Heidelberg, Germany.

PMID: 20374472  [PubMed - indexed for MEDLINE]

Click here for link to PubMed for article 20374472
```

----------------------------

----------------------------

```
Abstract 129 of 362

1. Med Phys. 2010 Feb;37(2):662-71.

Comparison of organ doses for patients undergoing balloon brachytherapy of the
breast with HDR 192Ir or electronic sources using monte carlo simulations in a
heterogeneous human phantom.

Mille MM, Xu XG, Rivard MJ.

Nuclear Engineering and Engineering Physics Program, Rensselaer Polytechnic
Institute, Troy, New York 12180, USA.

PMCID: PMC2905452
PMID: 20229875  [PubMed - indexed for MEDLINE]

Click here for link to PubMed for article 20229875
```

----------------------------

----------------------------

```
Abstract 130 of 362

1. J Extra Corpor Technol. 2009 Dec;41(4):206-12.

Virtual patient simulator for the perfusion resource management drill.

Ninomiya S, Tokaji M, Tokumine A, Kurosaki T.

Department of Clinical Engineering, Hiroshima International University,
Hiroshima, Japan.

PMID: 20092074  [PubMed - indexed for MEDLINE]

Click here for link to PubMed for article 20092074
```

----------------------------

----------------------------

```
Abstract 131 of 362

1. Acad Med. 2010 Jan;85(1):155-8. doi: 10.1097/ACM.0b013e3181c4f8bf.

Virtual patients: ED-2 band-aid or valuable asset in the learning portfolio?

Tworek J, Coderre S, Wright B, McLaughlin K.

Office of Undergraduate Medical Education, University of Calgary, Calgary,
Alberta, Canada.

PMID: 20042842  [PubMed - indexed for MEDLINE]

Click here for link to PubMed for article 20042842
```

----------------------------

----------------------------

```
Abstract 132 of 362

1. Int J Comput Assist Radiol Surg. 2009 Nov;4(6):561-70. doi:
10.1007/s11548-009-0371-5. Epub 2009 Jun 13.

An intersubject variable regional anesthesia simulator with a virtual patient
architecture.

Ullrich S, Grottke O, Fried E, Frommen T, Liao W, Rossaint R, Kuhlen T, Deserno
TM.

Virtual Reality Group, RWTH Aachen University, Aachen, Germany.

PMID: 20033332  [PubMed - indexed for MEDLINE]

Click here for link to PubMed for article 20033332
```

----------------------------

----------------------------

```
Abstract 133 of 362

1. Educ Health (Abingdon). 2009 Aug;22(2):269. Epub 2009 Jul 30.

Blended E-learning in a Web-based virtual hospital: a useful tool for
undergraduate education in urology.

Horstmann M, Renninger M, Hennenlotter J, Horstmann CC, Stenzl A.

Department of Urology, University of Tuebingen, Hoppe Seyler Strasse 3,
Tuebingen, Germany.

PMID: 20029750  [PubMed - indexed for MEDLINE]

Click here for link to PubMed for article 20029750
```

----------------------------

----------------------------

```
Abstract 134 of 362

1. Haematologica. 2010 Jun;95(6):900-7. doi: 10.3324/haematol.2009.015271. Epub 2009
Dec 8.

Tyrosine kinase inhibitor therapy can cure chronic myeloid leukemia without
hitting leukemic stem cells.

Lenaerts T, Pacheco JM, Traulsen A, Dingli D.

MLG, Département d'Informatique, Université Libre de Bruxelles, Brussels,
Belgium.

PMCID: PMC2878786
PMID: 20007137  [PubMed - indexed for MEDLINE]

Click here for link to PubMed for article 20007137
```

----------------------------

----------------------------

```
Abstract 135 of 362

1. Conf Proc IEEE Eng Med Biol Soc. 2009;2009:3901-4. doi:
10.1109/IEMBS.2009.5333563.

Numerical analysis of a comprehensive in silico subcutaneous insulin absorption
compartmental model.

Sebald D, Ruchti T.

Global Device R&D, Department of Hospira, Inc., Lake Forest, IL, USA.

PMID: 19964316  [PubMed - indexed for MEDLINE]

Click here for link to PubMed for article 19964316
```

----------------------------

----------------------------

```
Abstract 136 of 362

1. PLoS Comput Biol. 2009 Nov;5(11):e1000554. doi: 10.1371/journal.pcbi.1000554.
Epub 2009 Nov 26.

Nutritional systems biology modeling: from molecular mechanisms to physiology.

de Graaf AA, Freidig AP, De Roos B, Jamshidi N, Heinemann M, Rullmann JA, Hall
KD, Adiels M, van Ommen B.

Biosciences, TNO Quality of Life, Zeist, The Netherlands.

PMCID: PMC2777333
PMID: 19956660  [PubMed - indexed for MEDLINE]

Click here for link to PubMed for article 19956660
```

----------------------------

----------------------------

```
Abstract 137 of 362

1. Altern Lab Anim. 2009 Sep;37 Suppl 1:39-45.

Use of virtual patient populations for rescuing discontinued drug candidates and 
for reducing the number of patients in clinical trials.

Kleiman M, Sagi Y, Bloch N, Agur Z.

Optimata Ltd, Ramat-Gan, Israel.

2009 FRAME.

PMID: 19807203  [PubMed - indexed for MEDLINE]

Click here for link to PubMed for article 19807203
```

----------------------------

----------------------------

```
Abstract 138 of 362

1. J Dent Educ. 2009 Nov;73(11):1260-4.

Second life for dental education.

Phillips J, Berge ZL.

Department of Health Promotion and Policy, University of Maryland/Baltimore
College of Dental Surgery, MD, USA.

PMID: 19910474  [PubMed - indexed for MEDLINE]

Click here for link to PubMed for article 19910474
```

----------------------------

----------------------------

```
Abstract 139 of 362

1. J Healthc Qual. 2009 Sep-Oct;31(5):48-52.

Virtual patient safety rounds: one hospital system's approach to sharing
knowledge.

Graham JM, Brinson M, Magtibay LV, Regan B, Lazar EJ.

New York-Presbyterian Healthcare System, New York, NY, USA.

PMID: 19813561  [PubMed - indexed for MEDLINE]

Click here for link to PubMed for article 19813561
```

----------------------------

----------------------------

```
Abstract 140 of 362

1. Med Teach. 2009 Aug;31(8):764-9.

Training staff to create simple interactive virtual patients: the impact on a
medical and healthcare institution.

Round J, Conradi E, Poulton T.

St George's University of London, UK.

PMID: 19811216  [PubMed - indexed for MEDLINE]

Click here for link to PubMed for article 19811216
```

----------------------------

----------------------------

```
Abstract 141 of 362

1. Med Teach. 2009 Aug;31(8):759-63.

Improving assessment with virtual patients.

Round J, Conradi E, Poulton T.

St. George's University of London, UK.

PMID: 19811215  [PubMed - indexed for MEDLINE]

Click here for link to PubMed for article 19811215
```

----------------------------

----------------------------

```
Abstract 142 of 362

1. Med Teach. 2009 Aug;31(8):752-8.

The replacement of 'paper' cases by interactive online virtual patients in
problem-based learning.

Poulton T, Conradi E, Kavia S, Round J, Hilton S.

St George's, University of London, UK.

PMID: 19811214  [PubMed - indexed for MEDLINE]

Click here for link to PubMed for article 19811214
```

----------------------------

----------------------------

```
Abstract 143 of 362

1. Med Teach. 2009 Aug;31(8):749-51.

Creation of virtual patients from CT images of cadavers to enhance integration of
clinical and basic science student learning in anatomy.

Jacobson S, Epstein SK, Albright S, Ochieng J, Griffiths J, Coppersmith V, Polak 
JF.

Tufts University School of Medicine, Boston, MA, USA.

PMID: 19811213  [PubMed - indexed for MEDLINE]

Click here for link to PubMed for article 19811213
```

----------------------------

----------------------------

```
Abstract 144 of 362

1. Med Teach. 2009 Aug;31(8):743-8.

Towards a typology of virtual patients.

Huwendiek S, De leng BA, Zary N, Fischer MR, Ruiz JG, Ellaway R.

Heidelberg University, Germany.

PMID: 19811212  [PubMed - indexed for MEDLINE]

Click here for link to PubMed for article 19811212
```

----------------------------

----------------------------

```
Abstract 145 of 362

1. Med Teach. 2009 Aug;31(8):739-42.

The use of virtual patients to assess the clinical skills and reasoning of
medical students: initial insights on student acceptance.

Gesundheit N, Brutlag P, Youngblood P, Gunning WT, Zary N, Fors U.

Stanford University School of Medicine, California 94305-5404, USA.

PMID: 19811211  [PubMed - indexed for MEDLINE]

Click here for link to PubMed for article 19811211
```

----------------------------

----------------------------

```
Abstract 146 of 362

1. Med Teach. 2009 Aug;31(8):732-8.

Cross-cultural use and development of virtual patients.

Fors UG, Muntean V, Botezatu M, Zary N.

Karolinska Institutet, Sweden.

PMID: 19811210  [PubMed - indexed for MEDLINE]

Click here for link to PubMed for article 19811210
```

----------------------------

----------------------------

```
Abstract 147 of 362

1. Med Teach. 2009 Aug;31(8):721-4.

Online virtual patients - A driver for change in medical and healthcare
professional education in developing countries?

Dewhurst D, Borgstein E, Grant ME, Begg M.

University of Edinburgh, UK.

PMID: 19811208  [PubMed - indexed for MEDLINE]

Click here for link to PubMed for article 19811208
```

----------------------------

----------------------------

```
Abstract 148 of 362

1. Med Teach. 2009 Aug;31(8):713-20.

Virtual patients in a virtual world: Training paramedic students for practice.

Conradi E, Kavia S, Burden D, Rice A, Woodham L, Beaumont C, Savin-Baden M,
Poulton T.

St. George's University of London, UK.

PMID: 19811207  [PubMed - indexed for MEDLINE]

Click here for link to PubMed for article 19811207
```

----------------------------

----------------------------

```
Abstract 149 of 362

1. Med Teach. 2009 Aug;31(8):709-12.

The role of intellectual property in creating, sharing and repurposing virtual
patients.

Campbell G, Miller A, Balasubramaniam C.

Association of American Medical Colleges, Washington, DC 20037, USA.

PMID: 19811206  [PubMed - indexed for MEDLINE]

Click here for link to PubMed for article 19811206
```

----------------------------

----------------------------

```
Abstract 150 of 362

1. Med Teach. 2009 Aug;31(8):683-4.

Virtual patients come of age.

Ellaway RH, Poulton T, Smothers V, Greene P.

Northern Ontario School of Medicine, Canada.

PMID: 19811203  [PubMed - indexed for MEDLINE]

Click here for link to PubMed for article 19811203
```

----------------------------

----------------------------

```
Abstract 151 of 362

1. Med Teach. 2009 Sep;31(9):e397-401.

Medical education in Korea: the e-learning consortium.

Kim KJ, Han J, Park IeB, Kee C.

Sungkyunkwan University School of Medicine, Korea.

PMID: 19811175  [PubMed - indexed for MEDLINE]

Click here for link to PubMed for article 19811175
```

----------------------------

----------------------------

```
Abstract 152 of 362

1. Stud Health Technol Inform. 2009;150:826-30.

Enabling interoperability, accessibility and reusability of virtual patients
across Europe - design and implementation.

Zary N, Hege I, Heid J, Woodham L, Donkers J, Kononowicz AA.

Virtual Patient Lab, Department of LIME, Karolinska Institutet, 17177 Stockholm, 
Sweden.

PMID: 19745428  [PubMed - indexed for MEDLINE]

Click here for link to PubMed for article 19745428
```

----------------------------

----------------------------

```
Abstract 153 of 362

1. Stud Health Technol Inform. 2009;150:185-9.

Development and validation of strategies to test for interoperability of virtual 
patients.

Kononowicz AA, Heid J, Donkers J, Hege I, Woodham L, Zary N.

Department of Bioinformatics and Telemedicine, Jagiellonian University, Kraków,
Poland.

PMID: 19745294  [PubMed - indexed for MEDLINE]

Click here for link to PubMed for article 19745294
```

----------------------------

----------------------------

```
Abstract 154 of 362

1. Fam Med. 2009 Sep;41(8):594.

Are virtual patients a valid assessment tool?

Bardella IJ.

University of Colorado, USA.

Comment on
    Med Teach. 2008 Feb;30(1):17-24.

PMID: 19724946  [PubMed]

Click here for link to PubMed for article 19724946
```

----------------------------

----------------------------

```
Abstract 155 of 362

1. Vojnosanit Pregl. 2009 Jul;66(7):556-62.

Cost-effectiveness of four immunomodulatory therapies for relapsing-remitting
multiple sclerosis: a Markov model based on data a Balkan country in
socioeconomic transition.

Janković SM, Kostić M, Radosavljević M, Tesić D, Stefanović-Stoimenov N,
Stevanović I, Raković S, Aleksić J, Folić M, Aleksić A, Mihajlović I, Biorac N,
Borlja J, Vucković R.

University of Kragujevac, Medical Faculty, Ul. Svetozara Markovića 69, 34 000
Kragujevac, Serbia.

PMID: 19678581  [PubMed - indexed for MEDLINE]

Click here for link to PubMed for article 19678581
```

----------------------------

----------------------------

```
Abstract 156 of 362

1. Am J Rhinol Allergy. 2009 Jul-Aug;23(4):442-7. doi: 10.2500/ajra.2009.23.3335.

Integration of patient-specific paranasal sinus computed tomographic data into a 
virtual surgical environment.

Parikh SS, Chan S, Agrawal SK, Hwang PH, Salisbury CM, Rafii BY, Varma G,
Salisbury KJ, Blevins NH.

Department of Otolaryngology-Head and Neck Surgery, Stanford University,
Stanford, California 94305-5739, USA.

PMID: 19671264  [PubMed - indexed for MEDLINE]

Click here for link to PubMed for article 19671264
```

----------------------------

----------------------------

```
Abstract 157 of 362

1. IEEE Trans Biomed Eng. 2009 Nov;56(11):2627-33. doi: 10.1109/TBME.2009.2028013.
Epub 2009 Jul 31.

A 3-D mixed-reality system for stereoscopic visualization of medical dataset.

Ferrari V, Megali G, Troia E, Pietrabissa A, Mosca F.

EndoCAS Center, Department of Oncology, Transplantation and Advanced Technologies
in Medicine, University of Pisa, Pisa, Italy.


PMID: 19651551  [PubMed - indexed for MEDLINE]

Click here for link to PubMed for article 19651551
```

----------------------------

----------------------------

```
Abstract 158 of 362

1. Am J Obstet Gynecol. 2009 Sep;201(3):328.e1-6. doi: 10.1016/j.ajog.2009.05.051.
Epub 2009 Jul 24.

Improving residents' understanding of issues, comfort levels, and patient needs
regarding screening for and diagnosing Down syndrome.

Kleinert HL, Lunney CA, Campbell L, Ferguson JE 2nd.

Human Development Institute, University of Kentucky and Chandler Medical Center, 
Lexington, KY, USA.

PMID: 19631921  [PubMed - indexed for MEDLINE]

Click here for link to PubMed for article 19631921
```

----------------------------

----------------------------

```
Abstract 159 of 362

1. Rev Med Chil. 2009 Mar;137(3):438-43. doi: /S0034-98872009000300018. Epub 2009
Jun 15.

A letter from the United States: the romance of medicine--voyages and heroes.

Richard V L.

PMID: 19621189  [PubMed - indexed for MEDLINE]

Click here for link to PubMed for article 19621189
```

----------------------------

----------------------------

```
Abstract 160 of 362

1. Stud Health Technol Inform. 2009;144:264-8.

Optimizing Clinical Training for the Treatment of PTSD Using Virtual Patients.

Kenny PG, Parsons TD, Rothbaum B, Difede J, Reger G, Rizzo A.

University of Southern California Institute for Creative Technologies, Marina Del
Rey, California, USA.

PMID: 19592778  [PubMed - in process]

Click here for link to PubMed for article 19592778
```

----------------------------

----------------------------

```
Abstract 161 of 362

1. Stud Health Technol Inform. 2009;144:122-4.

A Comparative Analysis between Experts and Novices Interacting with a Virtual
Patient with PTSD.

Kenny PG, Parsons TD, Rizzo A.

Institute for Creative Technologies, University of Southern California, Marina
Del Rey, CA 90292, USA.

PMID: 19592746  [PubMed - in process]

Click here for link to PubMed for article 19592746
```

----------------------------

----------------------------

```
Abstract 162 of 362

1. Math Med Biol. 2009 Dec;26(4):297-307. doi: 10.1093/imammb/dqp005. Epub 2009 Jul 
7.

Immune system-tumour efficiency ratio as a new oncological index for radiotherapy
treatment optimization.

Sotolongo-Grau O, Rodríguez-Pérez D, Santos-Miranda JA, Sotolongo-Costa O,
Antoranz JC.

Dipartimento de Física Matemática y de Fluidos, Facultad de Ciencias, Universidad
Nacional de Educación a Distancia, Senda del Rey 9, Madrid 28040, Spain.

PMID: 19584118  [PubMed - indexed for MEDLINE]

Click here for link to PubMed for article 19584118
```

----------------------------

----------------------------

```
Abstract 163 of 362

1. Acad Med. 2009 Jul;84(7):942-9. doi: 10.1097/ACM.0b013e3181a8c668.

Integration strategies for using virtual patients in clinical clerkships.

Berman N, Fall LH, Smith S, Levine DA, Maloney CG, Potts M, Siegel B,
Foster-Johnson L.

Department of Pediatrics, Dartmouth Medical School, Hanover, New Hampshire, USA.

PMID: 19550193  [PubMed - indexed for MEDLINE]

Click here for link to PubMed for article 19550193
```

----------------------------

----------------------------

```
Abstract 164 of 362

1. Br J Anaesth. 2009 Aug;103(2):291-7. doi: 10.1093/bja/aep140. Epub 2009 Jun 18.

Indices of pulmonary oxygenation in pathological lung states: an investigation
using high-fidelity, computational modelling.

Kathirgamanathan A, McCahon RA, Hardman JG.

University Department of Anaesthesia, Queen's Medical Centre, Nottingham NG7 2UH,
UK.

PMID: 19541678  [PubMed - indexed for MEDLINE]

Click here for link to PubMed for article 19541678
```

----------------------------

----------------------------

```
Abstract 165 of 362

1. Med Teach. 2009 Aug;31(8):701-8. doi: 10.1080/01421590902793867.

12 Tips: Guidelines for authoring virtual patient cases.

Posel N, Fleiszer D, Shore BM.

McGill University, Canada.

PMID: 19513927  [PubMed - indexed for MEDLINE]

Click here for link to PubMed for article 19513927
```

----------------------------

----------------------------

```
Abstract 166 of 362

1. Med Educ. 2009 Jun;43(6):580-8. doi: 10.1111/j.1365-2923.2009.03369.x.

Design principles for virtual patients: a focus group study among students.

Huwendiek S, Reichert F, Bosse HM, de Leng BA, van der Vleuten CP, Haag M,
Hoffmann GF, Tönshoff B.

Department of Paediatrics, University Children's Hospital, Heidelberg, Germany.

PMID: 19493183  [PubMed - indexed for MEDLINE]

Click here for link to PubMed for article 19493183
```

----------------------------

----------------------------

```
Abstract 167 of 362

1. Yakugaku Zasshi. 2009 Jun;129(6):749-57.

Clinical trial simulations for dosage optimization of docetaxel in patients with 
liver dysfunction, based on a log-binominal regression for febrile neutropenia.

Ozawa K, Minami H, Sato H.

Department of Clinical and Molecular Pharmacokinetics/Pharmacodynamics, School of
Pharmaceutical Sciences, Showa University, Tokyo, Japan.

PMID: 19483418  [PubMed - indexed for MEDLINE]

Click here for link to PubMed for article 19483418
```

----------------------------

----------------------------

```
Abstract 168 of 362

1. Int J Med Robot. 2009 Sep;5(3):267-75. doi: 10.1002/rcs.257.

Optimized port placement for in vivo biosensors.

King BW, Reisner LA, Ellis RD, Klein MD, Auner GW, Pandya AK.

Department of Electrical and Computer Engineering, Wayne State University,
Detroit, MI 48202, USA.

PMID: 19402052  [PubMed - indexed for MEDLINE]

Click here for link to PubMed for article 19402052
```

----------------------------

----------------------------

```
Abstract 169 of 362

1. Stud Health Technol Inform. 2009;142:395-7.

The use of virtual reality simulation of head trauma in a surgical boot camp.

Vergara VM, Panaiotis, Kingsley D, Alverson DC, Godsmith T, Xia S, Caudell TP.

The University of New Mexico, New Mexico, USA.

PMID: 19377192  [PubMed - indexed for MEDLINE]

Click here for link to PubMed for article 19377192
```

----------------------------

----------------------------

```
Abstract 170 of 362

1. Stud Health Technol Inform. 2009;142:224-9.

Integrating cognitive simulation into the Maryland virtual patient.

Nirenburg S, McShane M, Beale S, Jarrell B, Fantry G.

University of Maryland, Baltimore, Maryland, USA.

PMID: 19377155  [PubMed - indexed for MEDLINE]

Click here for link to PubMed for article 19377155
```

----------------------------

----------------------------

```
Abstract 171 of 362

1. Med Educ. 2009 Apr;43(4):303-11. doi: 10.1111/j.1365-2923.2008.03286.x.

Virtual patients: a critical literature review and proposed next steps.

Cook DA, Triola MM.

Division of General Internal Medicine, Mayo Clinic College of Medicine,
Rochester, Minnesota 55905, USA.

Comment in
    Med Educ. 2012 Feb;46(2):235.

PMID: 19335571  [PubMed - indexed for MEDLINE]

Click here for link to PubMed for article 19335571
```

----------------------------

----------------------------

```
Abstract 172 of 362

1. Methods Inf Med. 2009;48(2):190-5. doi: 10.3414/ME9217. Epub 2009 Feb 18.

The impact of SOA for achieving healthcare interoperability. An empirical
investigation based on a hypothetical adoption.

Daskalakis S, Mantas J.

National and Kapodistrian University of Athens, 115 27 Athens, Greece.

PMID: 19283318  [PubMed - indexed for MEDLINE]

Click here for link to PubMed for article 19283318
```

----------------------------

----------------------------

```
Abstract 173 of 362

1. J Korean Med Sci. 2009 Feb;24(1):7-12. doi: 10.3346/jkms.2009.24.1.7. Epub 2009
Feb 28.

Simulation of the AUC changes after generic substitution in patients.

Yim DS.

Department of Pharmacology, College of Medicine, The Catholic University of
Korea, Seoul, Korea.

PMCID: PMC2650971
PMID: 19270806  [PubMed - indexed for MEDLINE]

Click here for link to PubMed for article 19270806
```

----------------------------

----------------------------

```
Abstract 174 of 362

1. Otolaryngol Head Neck Surg. 2009 Mar;140(3):398-402. doi:
10.1016/j.otohns.2008.11.033.

Interactive Internet-based cases for undergraduate otolaryngology education.

Kandasamy T, Fung K.

Schulich School of Medicine and Dentistry, Division of Head and Neck Oncology and
Reconstructive Surgery, University of Western Ontario, London, Ontario, Canada.

PMID: 19248951  [PubMed - indexed for MEDLINE]

Click here for link to PubMed for article 19248951
```

----------------------------

----------------------------

```
Abstract 175 of 362

1. Eur J Dent Educ. 2009 Feb;13(1):2-9. doi: 10.1111/j.1600-0579.2007.00470.x.

Web-based virtual patients in dentistry: factors influencing the use of cases in 
the Web-SP system.

Zary N, Johnson G, Fors U.

Virtual Patient Lab, Karolinska Institute, Berzelius väg 3, Stockholm, Sweden.

PMID: 19196286  [PubMed - indexed for MEDLINE]

Click here for link to PubMed for article 19196286
```

----------------------------

----------------------------

```
Abstract 176 of 362

1. World Hosp Health Serv. 2008;44(3):36-9.

Virtual patient simulation for prevention of medical error: beyond just technical
upskilling.

McConnell H, Pardy A.

Griffith University School of Medicine, Australia.

PMID: 19181024  [PubMed - indexed for MEDLINE]

Click here for link to PubMed for article 19181024
```

----------------------------

----------------------------

```
Abstract 177 of 362

1. Am J Surg. 2009 Jan;197(1):102-6. doi: 10.1016/j.amjsurg.2008.08.012.

A pilot study to integrate an immersive virtual patient with a breast complaint
and breast examination simulator into a surgery clerkship.

Deladisma AM, Gupta M, Kotranza A, Bittner JG 4th, Imam T, Swinson D, Gucwa A,
Nesbit R, Lok B, Pugh C, Lind DS.

Department of Surgery, Medical College of Georgia, School of Medicine, 1120 15th 
St., Augusta, GA 30912, USA.

PMID: 19101251  [PubMed - indexed for MEDLINE]

Click here for link to PubMed for article 19101251
```

----------------------------

----------------------------

```
Abstract 178 of 362

1. Strabismus. 2008;16(4):165-9. doi: 10.1080/09273970802450804.

Computer-based simulation of the Bielschowsky head-tilt test using the SEE++
software system.

Kaltofen T, Buchberger M, Priglinger S.

RISC Software GmbH, Research Unit Medical-Informatics, Hagenberg, Austria.

PMID: 19089762  [PubMed - indexed for MEDLINE]

Click here for link to PubMed for article 19089762
```

----------------------------

----------------------------

```
Abstract 179 of 362

1. Simul Healthc. 2008 Winter;3(4):242-6. doi: 10.1097/SIH.0b013e3181871b58.

High degree of realism in teaching percutaneous coronary interventions by
combining a virtual reality trainer with a full scale patient simulator.

Schuetz M, Moenk S, Vollmer J, Kurz S, Mollnau H, Post F, Heinrichs W.

Department of Anesthesiology, University of Mainz, Mainz, Rhineland-Palatinate,
Germany.

PMID: 19088669  [PubMed - indexed for MEDLINE]

Click here for link to PubMed for article 19088669
```

----------------------------

----------------------------

```
Abstract 180 of 362

1. J Nurs Res. 2008 Dec;16(4):275-85.

The development of a competency-based group health teaching performance
examination model for BSN graduates.

Tai CY, Chung UL.

Department of Nursing, National Taipei College of Nursing.

PMID: 19061174  [PubMed - indexed for MEDLINE]

Click here for link to PubMed for article 19061174
```

----------------------------

----------------------------

```
Abstract 181 of 362

1. AMIA Annu Symp Proc. 2008 Nov 6:1140.

eViP: sharing virtual patients across Europe.

Smothers V, Ellaway R, Balasubramaniam C.

MedBiquitous, Baltimore, MD, USA.

PMID: 18998985  [PubMed - indexed for MEDLINE]

Click here for link to PubMed for article 18998985
```

----------------------------

----------------------------

```
Abstract 182 of 362

1. AMIA Annu Symp Proc. 2008 Nov 6:1150.

Value of information in virtual patients portraying pharyngitis.

Sumner W 2nd, Hagen MD.

Washington University School of Medicine, St. Louis, MO, USA.

PMID: 18998813  [PubMed - indexed for MEDLINE]

Click here for link to PubMed for article 18998813
```

----------------------------

----------------------------

```
Abstract 183 of 362

1. AMIA Annu Symp Proc. 2008 Nov 6:1149.

Value of information in virtual patient performance evaluations.

Sumner W 2nd, Hagen MD.

Washington University School of Medicine, St. Louis, MO, USA.

PMID: 18998811  [PubMed - indexed for MEDLINE]

Click here for link to PubMed for article 18998811
```

----------------------------

----------------------------

```
Abstract 184 of 362

1. Med Educ. 2008 Nov;42(11):1120-1. doi: 10.1111/j.1365-2923.2008.03194.x.

Virtual patients and undergraduate anaesthesia teaching.

Critchley LA, Wong JW, Leung JY.

Department of Anaesthesia and Intensive Care, Prince of Wales Hospital, Shatin,
New Territories, Hong Kong, China.

PMID: 18991996  [PubMed - indexed for MEDLINE]

Click here for link to PubMed for article 18991996
```

----------------------------

----------------------------

```
Abstract 185 of 362

1. Acad Emerg Med. 2008 Nov;15(11):1152-9. doi: 10.1111/j.1553-2712.2008.00223.x.
Epub 2008 Oct 25.

Using immersive simulation for training first responders for mass casualty
incidents.

Wilkerson W, Avstreih D, Gruppen L, Beier KP, Woolliscroft J.

Department of Emergency Medicine, University of Michigan, Ann Arbor, MI, USA.

PMID: 18976333  [PubMed - indexed for MEDLINE]

Click here for link to PubMed for article 18976333
```

----------------------------

----------------------------

```
Abstract 186 of 362

1. Prim Health Care Res Dev. 2008 Oct 1;9(4):257-268.

Virtual standardized patients: an interactive method to examine variation in
depression care among primary care physicians.

Hooper LM, Weinfurt KP, Cooper LA, Mensh J, Harless W, Kuhajda MC, Epstein SA.

Department of Educational Studies in Psychology, Research Methodology, &
Counseling, The University of Alabama, Tuscaloosa, AL, USA.

PMCID: PMC2867621
PMID: 20463864  [PubMed]

Click here for link to PubMed for article 20463864
```

----------------------------

----------------------------

```
Abstract 187 of 362

1. Cochlear Implants Int. 2003 Jun;4(2):96-101. doi: 10.1002/cii.69.

How we do it: using a surgical navigation system in the management of the
ossified cochlea.

Raine CH, Strachan DR, Gopichandran T.

Yorkshire Cochlear Implant Unit, Bradford Royal Infirmary, Bradford BD9 6RJ, UK. 

PMID: 18792141  [PubMed]

Click here for link to PubMed for article 18792141
```

----------------------------

----------------------------

```
Abstract 188 of 362

1. Spec Care Dentist. 2008 Sep-Oct;28(5):205-13. doi:
10.1111/j.1754-4505.2008.00038.x.

Virtual patient instruction for dental students: can it improve dental care
access for persons with special needs?

Sanders C, Kleinert HL, Boyd SE, Herren C, Theiss L, Mink J.

Human Development Institute, University of Kentucky, USA. 

PMID: 18782198  [PubMed - indexed for MEDLINE]

Click here for link to PubMed for article 18782198
```

----------------------------

----------------------------

```
Abstract 189 of 362

1. J Midwifery Womens Health. 2008 Sep-Oct;53(5):453-60. doi:
10.1016/j.jmwh.2008.04.017.

Virtual patient training to improve reproductive health care for women with
intellectual disabilities.

Boyd SE, Sanders CL, Kleinert HL, Huff MB, Lock S, Johnson S, Clevenger K, Bush
NA, Van Dyke E, Clark TL.

Human Development Institute, 8 Mineral Industries Bldg., University of Kentucky, 
Lexington, KY 40506, USA.

PMID: 18761299  [PubMed - indexed for MEDLINE]

Click here for link to PubMed for article 18761299
```

----------------------------

----------------------------

```
Abstract 190 of 362

1. AMIA Annu Symp Proc. 2007 Oct 11:741-5.

An XML standard for virtual patients: exchanging case-based simulations in
medical education.

Triola MM, Campion N, McGee JB, Albright S, Greene P, Smothers V, Ellaway R.

New York University School of Medicine, New York, NY, USA.

PMCID: PMC2655833
PMID: 18693935  [PubMed - indexed for MEDLINE]

Click here for link to PubMed for article 18693935
```

----------------------------

----------------------------

```
Abstract 191 of 362

1. AMIA Annu Symp Proc. 2007 Oct 11:706-10.

Modeling relief.

Sumner W 2nd, Xu JZ, Roussel G, Hagen MD.

Washington University, St. Louis, Missouri, USA.

PMCID: PMC2655778
PMID: 18693928  [PubMed - indexed for MEDLINE]

Click here for link to PubMed for article 18693928
```

----------------------------

----------------------------

```
Abstract 192 of 362

1. AMIA Annu Symp Proc. 2007 Oct 11:181-5.

Virtual patient model for multi-person virtual medical environments.

Dev P, Heinrichs WL, Youngblood P, Kung S, Cheng R, Kusumoto L, Hendrick A.

Stanford University, Stanford, CA, USA.

PMCID: PMC2655782
PMID: 18693822  [PubMed - indexed for MEDLINE]

Click here for link to PubMed for article 18693822
```

----------------------------

----------------------------

```
Abstract 193 of 362

1. Physiol Meas. 2008 Aug;29(8):959-78. doi: 10.1088/0967-3334/29/8/008. Epub 2008
Jul 18.

A simulation model of glucose regulation in the critically ill.

Hovorka R, Chassin LJ, Ellmerer M, Plank J, Wilinska ME.

Institute of Metabolic Science, Metabolic Research Laboratories, Level 4, Box
289, Addenbrooke's Hospital, University of Cambridge, Hills Road, Cambridge CB2
0QQ, UK. 

PMID: 18641427  [PubMed - indexed for MEDLINE]

Click here for link to PubMed for article 18641427
```

----------------------------

----------------------------

```
Abstract 194 of 362

1. Clin Cardiol. 2008 Jul;31(7):334-9. doi: 10.1002/clc.20213.

Using virtual patients to improve cardiac examination competency in medical
students.

Vukanovic-Criley JM, Boker JR, Criley SR, Rajagopalan S, Criley JM.

David L Geffen School of Medicine at UCLA, St. Mary's Medical Center, Long Beach,
CA 90813, USA. 

Copyright (c) 2008 Wiley Periodicals, Inc.

PMID: 18636531  [PubMed - indexed for MEDLINE]

Click here for link to PubMed for article 18636531
```

----------------------------

----------------------------

```
Abstract 195 of 362

1. Int J Orthod Milwaukee. 2008 Spring;19(1):15-21.

Three dimensional computed technology--a new standard of care.

Tipton WL, Metz P.

PMID: 18512657  [PubMed - indexed for MEDLINE]

Click here for link to PubMed for article 18512657
```

----------------------------

----------------------------

```
Abstract 196 of 362

1. Clin Oral Implants Res. 2008 Jul;19(7):709-16. doi:
10.1111/j.1600-0501.2007.01430.x. Epub 2008 May 19.

Evaluation of the accuracy of three different computer-aided surgery systems in
dental implantology: optical tracking vs. stereolithographic splint systems.

Ruppin J, Popovic A, Strauss M, Spüntrup E, Steiner A, Stoll C.

PMID: 18492085  [PubMed - indexed for MEDLINE]

Click here for link to PubMed for article 18492085
```

----------------------------

----------------------------

```
Abstract 197 of 362

1. Stud Health Technol Inform. 2008;136:285-90.

Evaluating the impact of a service-oriented framework for healthcare
interoperability.

Daskalakis S, Mantas J.

Health Informatics Laboratory, Faculty of Nursing, National and Kapodistrian
University of Athens, Greece.

PMID: 18487745  [PubMed - indexed for MEDLINE]

Click here for link to PubMed for article 18487745
```

----------------------------

----------------------------

```
Abstract 198 of 362

1. Med Teach. 2008;30(3):e66-76. doi: 10.1080/01421590801910216.

The use of a virtual patient case in an OSCE-based exam--a pilot study.

Courteille O, Bergin R, Stockeld D, Ponzer S, Fors U.

Karolinska Institutet, Stockholm, Sweden.

PMID: 18484444  [PubMed - indexed for MEDLINE]

Click here for link to PubMed for article 18484444
```

----------------------------

----------------------------

```
Abstract 199 of 362

1. Diabetes Technol Ther. 2008 Jun;10(3):178-87. doi: 10.1089/dia.2007.0281.

How would patients behave if they were continually informed of their blood
glucose levels? A simulation study using a "virtual" patient.

Biermann E, Barkhausen K, Standl E.

Staedtisches Klinikum Muenchen GmbH, Klinikum Schwabing, Muenchen, Germany.

PMID: 18473691  [PubMed - indexed for MEDLINE]

Click here for link to PubMed for article 18473691
```

----------------------------

----------------------------

```
Abstract 200 of 362

1. Med Teach. 2008;30(2):170-4. doi: 10.1080/01421590701874074.

Building a virtual patient commons.

Ellaway R, Poulton T, Fors U, McGee JB, Albright S.

University of London, UK.

PMID: 18464142  [PubMed - indexed for MEDLINE]

Click here for link to PubMed for article 18464142
```

----------------------------

----------------------------

```
Abstract 201 of 362

1. J Diabetes Sci Technol. 2008 May;2(3):436-49.

In silico simulation of long-term type 1 diabetes glycemic control treatment
outcomes.

Wong XW, Chase JG, E Hann C, F Lotz T, Lin J, Le Compte AJ, Shaw GM.

Department of Mechanical Engineering, University of Canterbury, Christchurch, New
Zealand.

PMCID: PMC2769739
PMID: 19885208  [PubMed]

Click here for link to PubMed for article 19885208
```

----------------------------

----------------------------

```
Abstract 202 of 362

1. J Diabetes Sci Technol. 2008 May;2(3):424-35.

Development of a clinical type 1 diabetes metabolic system model and in silico
simulation tool.

Wong XW, Chase JG, Hann CE, Lotz TF, Lin J, Le AJ, Shaw GM.

Department of Mechanical Engineering, University of Canterbury, Christchurch, New
Zealand.

PMCID: PMC2769735
PMID: 19885207  [PubMed]

Click here for link to PubMed for article 19885207
```

----------------------------

----------------------------

```
Abstract 203 of 362

1. Stud Health Technol Inform. 2008;132:484-6.

Virtual patient monitors for new user familiarization.

Sprick C, Ruthenbeck GS, Owen H, Reynolds KJ.

School of Informatics & Engineering, Flinders University, South Australia.

PMID: 18391350  [PubMed - indexed for MEDLINE]

Click here for link to PubMed for article 18391350
```

----------------------------

----------------------------

```
Abstract 204 of 362

1. Stud Health Technol Inform. 2008;132:366-71.

Virtual reality training for radiotherapy becomes a reality.

Phillips R, Ward JW, Page L, Grau C, Bojen A, Hall J, Nielsen K, Nordentoft V,
Beavis AW.

Department of Computer Science, University of Hull, East Riding of Yorkshire, UK.

PMID: 18391323  [PubMed - indexed for MEDLINE]

Click here for link to PubMed for article 18391323
```

----------------------------

----------------------------

```
Abstract 205 of 362

1. Stud Health Technol Inform. 2008;132:357-62.

Objective structured clinical interview training using a virtual human patient.

Parsons TD, Kenny P, Ntuen CA, Pataki CS, Pato MT, Rizzo AA, St-George C, Sugar
J.

Institute for Creative Technologies, University of Southern California, Los
Angeles, CA 90292-4019, USA.

PMID: 18391321  [PubMed - indexed for MEDLINE]

Click here for link to PubMed for article 18391321
```

----------------------------

----------------------------

```
Abstract 206 of 362

1. Stud Health Technol Inform. 2008;132:281-6.

Revealing the conceptual substrate of biomedical cognitive models to the wider
community.

McShane M, Jarrell B, Fantry G, Nirenburg S, Beale S, Johnson B.

University of Maryland Baltimore County, MD, USA.

PMID: 18391305  [PubMed - indexed for MEDLINE]

Click here for link to PubMed for article 18391305
```

----------------------------

----------------------------

```
Abstract 207 of 362

1. Stud Health Technol Inform. 2008;132:159-64.

Design and implementation of rule-based medical models: an In Silico
patho-physiological trauma model for hypovolemic shock.

Heinrichs WL, Kung SY, Dev P.

SUMMIT (Stanford University Medical Media and Information Technologies) Stanford 
University School of Medicine, Stanford, CA 94305-5466, USA.

PMID: 18391278  [PubMed - indexed for MEDLINE]

Click here for link to PubMed for article 18391278
```

----------------------------

----------------------------

```
Abstract 208 of 362

1. Stud Health Technol Inform. 2008;132:101-5.

Medical student satisfaction using a virtual patient system to learn
history-taking communication skills.

Deladisma AM, Johnsen K, Raij A, Rossen B, Kotranza A, Kalapurakal M, Szlam S,
Bittner JG 4th, Swinson D, Lok B, Lind DS.

Department of Surgery, Medical College of Georgia, Augusta, GA 30912, USA.

PMID: 18391266  [PubMed - indexed for MEDLINE]

Click here for link to PubMed for article 18391266
```

----------------------------

----------------------------

```
Abstract 209 of 362

1. J Nurs Educ. 2008 Feb;47(2):66-73.

Developmental disabilities: improving competence in care using virtual patients.

Sanders CL, Kleinert HL, Free T, King P, Slusher I, Boyd S.

Human Development Institute, 126 Mineral Industries Building, University of
Kentucky, Lexington, KY 40506, USA.

PMID: 18320957  [PubMed - indexed for MEDLINE]

Click here for link to PubMed for article 18320957
```

----------------------------

----------------------------

```
Abstract 210 of 362

1. Zentralbl Chir. 2008 Feb;133(1):51-4. doi: 10.1055/s-2008-1004666.

[The quality of patient care under the German DRG system using as example the
inguinal hernia repair].

[Article in German]

Rudroff C, Schweins M, Heiss MM.

Klinik für Viszeral-, Gefäb- und Transplantationschirurgie, Kliniken der Stadt
Köln gGmbH, Krankenhaus Köln-Merheim, Lehrstuhl für Chirurgie I der Universität
Witten-Herdecke.

PMID: 18278703  [PubMed - indexed for MEDLINE]

Click here for link to PubMed for article 18278703
```

----------------------------

----------------------------

```
Abstract 211 of 362

1. Med Teach. 2008 Feb;30(1):17-24. doi: 10.1080/01421590701758616.

Virtual patients for assessing medical students--important aspects when
considering the introduction of a new assessment format.

Waldmann UM, Gulich MS, Zeitler HP.

Institute of General Practice, University of Ulm, Hemholtzstrasse 20, D-89069
Ulm, Germany.

Comment in
    Fam Med. 2009 Sep;41(8):594.

PMID: 18278646  [PubMed - indexed for MEDLINE]

Click here for link to PubMed for article 18278646
```

----------------------------

----------------------------

```
Abstract 212 of 362

1. Comput Methods Programs Biomed. 2008 Jun;90(3):240-50. doi:
10.1016/j.cmpb.2007.12.008. Epub 2008 Feb 13.

A SAS-based solution to evaluate study design efficiency of phase I pediatric
oncology trials via discrete event simulation.

Barrett JS, Jayaraman B, Patel D, Skolnik JM.

Clinical Pharmacology & Therapeutics Division, The Children's Hospital of
Philadelphia, USA.

PMID: 18276034  [PubMed - indexed for MEDLINE]

Click here for link to PubMed for article 18276034
```

----------------------------

----------------------------

```
Abstract 213 of 362

1. Gerontol Geriatr Educ. 2008;28(3):73-88. doi: 10.1300/J021v28n03_06.

E-learning virtual patients for geratric education.

Orton E, Mulhausen P.

Division of General Internal Medicine, Department of Internal Medicine,
University of Iowa, Carver College of Medicine, 2153 Westlawn, Iowa City, IA
52242, USA.

PMID: 18215989  [PubMed - indexed for MEDLINE]

Click here for link to PubMed for article 18215989
```

----------------------------

----------------------------

```
Abstract 214 of 362

1. Int J Artif Organs. 2007 Dec;30(12):1047-56.

Gas exchange in a virtual respiratory system--simulation of ventilation without
lung movement.

Golczewski T.

Institute of Biocybernetics and Biomedical Engineering, Polish Academy of
Sciences, Warsaw, Poland.

PMID: 18203066  [PubMed - indexed for MEDLINE]

Click here for link to PubMed for article 18203066
```

----------------------------

----------------------------

```
Abstract 215 of 362

1. Am J Hosp Palliat Care. 2008 Apr-May;25(2):127-31. doi: 10.1177/1049909107310142.
Epub 2008 Jan 15.

Use of thromboprophylaxis in palliative care patients: a survey among experts in 
palliative care, oncology, intensive care, and anticoagulation.

Kierner KA, Gartner V, Schwarz M, Watzke HH.

Unit of Palliative Care, Department of Medicine I, Medical University of Vienna, 
Austria.

PMID: 18198364  [PubMed - indexed for MEDLINE]

Click here for link to PubMed for article 18198364
```

----------------------------

----------------------------

```
Abstract 216 of 362

1. Nurs Manage. 2007 Dec;38(12):27-33. doi: 10.1097/01.NUMA.0000303868.95890.1e.

Virtual patients support point-of-care nursing education.

Curran C, Sheets D, Kirkpatrick B, Bauldoff GS.

OSU College of Nursing, Ohio State University, OH, USA.

PMID: 18188006  [PubMed - indexed for MEDLINE]

Click here for link to PubMed for article 18188006
```

----------------------------

----------------------------

```
Abstract 217 of 362

1. Int J Clin Pharmacol Ther. 2007 Dec;45(12):623-30.

Insulin glargine added to therapy with oral antidiabetic agents improves glycemic
control and reduces long-term complications in patients with type 2 diabetes - a 
simulation with the Diabetes Mellitus Model (DMM).

Janka HU, Hessel F, Walzer S, Mã Ller E.

Klinikum Bremen Nord, Bremen, Germany.

PMID: 18184530  [PubMed - indexed for MEDLINE]

Click here for link to PubMed for article 18184530
```

----------------------------

----------------------------

```
Abstract 218 of 362

1. Z Evid Fortbild Qual Gesundhwes. 2008;102(10):648-53.

[Virtual patients in medical education: a comparison of various strategies for
curricular integration].

[Article in German]

Fischer MR, Hege I, Hörnlein A, Puppe F, Tönshoff B, Huwendiek S.

Schwerpunkt Medizindidaktik, Medizinische Klinik-Innenstadt, Klinikum der
Universität München.

PMID: 19402352  [PubMed - indexed for MEDLINE]

Click here for link to PubMed for article 19402352
```

----------------------------

----------------------------

```
Abstract 219 of 362

1. J Neurosurg. 2007 Dec;107(6 Suppl):474-8.

Monte Carlo simulation of cerebrospinal fluid shunt failure and definition of
instability among shunt-treated patients with hydrocephalus.

Piatt JH Jr, Cosgriff M.

Section of Neurosurgery, St. Christopher's Hospital for Children, Philadelphia,
Pennsylvania 19134-1095, USA.

PMID: 18154016  [PubMed - indexed for MEDLINE]

Click here for link to PubMed for article 18154016
```

----------------------------

----------------------------

```
Abstract 220 of 362

1. Eur J Surg Oncol. 2007 Dec;33 Suppl 2:S111-7. Epub 2007 Nov 28.

Computer-based decision making in medicine: A model for surgery of colorectal
liver metastases.

Langenhoff BS, Krabbe PF, Ruers TJ.

Department of Surgery, Radboud University Nijmegen Medical Centre, P.O. Box 9101,
6500 HB Nijmegen, The Netherlands.

PMID: 18053676  [PubMed - indexed for MEDLINE]

Click here for link to PubMed for article 18053676
```

----------------------------

----------------------------

```
Abstract 221 of 362

1. Ann Biomed Eng. 2008 Feb;36(2):321-34. Epub 2007 Nov 29.

Integrating epidemiological data into a mechanistic model of type 2 diabetes:
validating the prevalence of virtual patients.

Klinke DJ 2nd.

Department of Chemical Engineering, West Virginia University, P.O. Box 6102,
Morgantown, WV 25606, USA.

PMID: 18046647  [PubMed - indexed for MEDLINE]

Click here for link to PubMed for article 18046647
```

----------------------------

----------------------------

```
Abstract 222 of 362

1. Anesth Analg. 2007 Dec;105(6):1629-38, table of contents.

Estimation of optimal modeling weights for a Bayesian-based closed-loop system
for propofol administration using the bispectral index as a controlled variable: 
a simulation study.

De Smet T, Struys MM, Greenwald S, Mortier EP, Shafer SL.

Demed Engineering, Temse, Belgium.

PMID: 18042860  [PubMed - indexed for MEDLINE]

Click here for link to PubMed for article 18042860
```

----------------------------

----------------------------

```
Abstract 223 of 362

1. J Pediatr Nurs. 2007 Dec;22(6):457-66.

Caring for children with intellectual and developmental disabilities: virtual
patient instruction improves students' knowledge and comfort level.

Sanders CL, Kleinert HL, Free T, Slusher I, Clevenger K, Johnson S, Boyd SE.

Human Development Institute, University of Kentucky, Lexington, KY 40509, USA.

PMID: 18036466  [PubMed - indexed for MEDLINE]

Click here for link to PubMed for article 18036466
```

----------------------------

----------------------------

```
Abstract 224 of 362

1. Phys Med Biol. 2007 Dec 7;52(23):7153-66. Epub 2007 Nov 19.

Revealing the mechanisms underlying embolic stroke using computational modelling.

Chung EM, Hague JP, Evans DH.

Medical Physics Department, University Hospitals of Leicester NHS Trust,
Leicester, UK.

PMID: 18029999  [PubMed - indexed for MEDLINE]

Click here for link to PubMed for article 18029999
```

----------------------------

----------------------------

```
Abstract 225 of 362

1. Conf Proc IEEE Eng Med Biol Soc. 2007;2007:3292-5.

Towards optimal virtual patients: an online adaptive control approach.

Ghosh S, Young DL, Gadkar KG, Wennerberg L, Basu K.

Biological Networking Research Group, Center For Research In Wireless Mobility
and Networking (CReWMaN), The University of Texas at Arlington, Arlington, TX
76010, USA.

PMID: 18002699  [PubMed - indexed for MEDLINE]

Click here for link to PubMed for article 18002699
```

----------------------------

----------------------------

```
Abstract 226 of 362

1. Curr Drug Deliv. 2007 Oct;4(4):283-96.

Model-based insulin and nutrition administration for tight glycaemic control in
critical care.

Chase JG, Shaw GM, Lotz T, LeCompte A, Wong J, Lin J, Lonergan T, Willacy M, Hann
CE.

University of Canterbury, Department of Mechanical Engineering, Centre for
Bio-Engineering, Christchurch, New Zealand.

PMID: 17979649  [PubMed - indexed for MEDLINE]

Click here for link to PubMed for article 17979649
```

----------------------------

----------------------------

```
Abstract 227 of 362

1. Acta Anaesthesiol Taiwan. 2007 Sep;45(3):141-7.

Simulation analysis of the performance of target-controlled infusion of propofol 
in Chinese patients.

Ko YP, Hsu YW, Hsu K, Tsai HJ, Huang CJ, Chen CC.

Department of Anesthesiology, Hsinchu Mackay Memorial Hospital, Hsinchu, Taiwan, 
ROC.

PMID: 17972616  [PubMed - indexed for MEDLINE]

Click here for link to PubMed for article 17972616
```

----------------------------

----------------------------

```
Abstract 228 of 362

1. Zhongguo Yi Liao Qi Xie Za Zhi. 2007 Jul;31(4):291-2, 283.

[Development of a method to proofread and correct the 3D virtual patient in CT
simulation of radiotherapy].

[Article in Chinese]

Zhang Y, Tao JM, Hu J, Zhang Y.

Department of Radiotherapy, Shanghai Tenth People's Hospital, 200072, Shanghai.

PMID: 17969513  [PubMed - indexed for MEDLINE]

Click here for link to PubMed for article 17969513
```

----------------------------

----------------------------

```
Abstract 229 of 362

1. J Diabetes Sci Technol. 2007 Nov;1(6):804-12.

Model predictive control of type 1 diabetes: an in silico trial.

Magni L, Raimondo DM, Bossi L, Man CD, De Nicolao G, Kovatchev B, Cobelli C.

Dipartimento di Informatica e Sistemistica, University of Pavia, Pavia, Italy.

PMCID: PMC2769684
PMID: 19885152  [PubMed]

Click here for link to PubMed for article 19885152
```

----------------------------

----------------------------

```
Abstract 230 of 362

1. Nonlinear Biomed Phys. 2007 Jul 16;1(1):6.

Virtual respiratory system in investigation of CPAP influence on optimal
breathing frequency in obstructive lungs disease.

Golczewski T, Darowski M.

Institute of Biocybernetics and Biomedical Engineering, Polish Academy of
Sciences, Warsaw, Poland.

PMCID: PMC1997125
PMID: 17908339  [PubMed]

Click here for link to PubMed for article 17908339
```

----------------------------

----------------------------

```
Abstract 231 of 362

1. J Gastrointest Surg. 2007 Oct;11(10):1328-32. Epub 2007 Aug 8.

A decision analysis model identifies the interval of efficacy for transarterial
chemoembolization (TACE) in cirrhotic patients with hepatocellular carcinoma
awaiting liver transplantation.

Aloia TA, Adam R, Samuel D, Azoulay D, Castaing D.

Division of Abdominal Transplantation and Hepatobiliary Surgery, Department of
Surgery, Baylor College of Medicine, 1709 Dryden, Suite 15.37, Houston, TX 77030,
USA.

PMID: 17682827  [PubMed - indexed for MEDLINE]

Click here for link to PubMed for article 17682827
```

----------------------------

----------------------------

```
Abstract 232 of 362

1. Comput Methods Programs Biomed. 2008 Feb;89(2):141-52. Epub 2007 Jun 4.

Stochastic modelling of insulin sensitivity and adaptive glycemic control for
critical care.

Lin J, Lee D, Chase JG, Shaw GM, Le Compte A, Lotz T, Wong J, Lonergan T, Hann
CE.

Department of Mechanical Engineering, Centre for Bio-Engineering, University of
Canterbury, Christchurch, New Zealand.

PMID: 17544541  [PubMed - indexed for MEDLINE]

Click here for link to PubMed for article 17544541
```

----------------------------

----------------------------

```
Abstract 233 of 362

1. Med Teach. 2007 Feb;29(1):33-7.

Patient-centred learning--back to the future.

Smith SR, Cookson J, McKendree J, Harden RM.

Brown Medical School, Providence, RI 02912, USA.

PMID: 17538831  [PubMed - indexed for MEDLINE]

Click here for link to PubMed for article 17538831
```

----------------------------

----------------------------

```
Abstract 234 of 362

1. Am J Pharm Educ. 2007 Apr 15;71(2):30.

Integrating virtual patients into a self-care course.

Orr KK.

University of Rhode Island College of Pharmacy, USA.

PMCID: PMC1858613
PMID: 17533439  [PubMed - indexed for MEDLINE]

Click here for link to PubMed for article 17533439
```

----------------------------

----------------------------

```
Abstract 235 of 362

1. Int J Med Robot. 2006 Dec;2(4):312-20.

A novel laparoscopic mesh placement part task trainer.

Devarajan V, Wang X, Shen Y, Eberhart R, Watson MJ, Jones D, Villegas L.

The University of Texas at Arlington, TX 76016, USA.

Copyright 2006 John Wiley & Sons, Ltd.

PMID: 17520649  [PubMed - indexed for MEDLINE]

Click here for link to PubMed for article 17520649
```

----------------------------

----------------------------

```
Abstract 236 of 362

1. Am J Surg. 2007 Jun;193(6):756-60.

Do medical students respond empathetically to a virtual patient?

Deladisma AM, Cohen M, Stevens A, Wagner P, Lok B, Bernard T, Oxendine C,
Schumacher L, Johnsen K, Dickerson R, Raij A, Wells R, Duerson M, Harper JG, Lind
DS; Association for Surgical Education.

Department of Surgery, Medical College of Georgia, 1120 15th Street, Augusta, GA 
30912, USA.

PMID: 17512291  [PubMed - indexed for MEDLINE]

Click here for link to PubMed for article 17512291
```

----------------------------

----------------------------

```
Abstract 237 of 362

1. Br J Gen Pract. 2007 May;57(538):401-3.

Which test is best for Helicobacter pylori? A cost-effectiveness model using
decision analysis.

Elwyn G, Taubert M, Davies S, Brown G, Allison M, Phillips C.

Department of Primary Care and Public Health, Cardiff University, Neuadd
Meirionnydd Heath Park, CF14 4YS,Wales.

PMCID: PMC2047016
PMID: 17504592  [PubMed - indexed for MEDLINE]

Click here for link to PubMed for article 17504592
```

----------------------------

----------------------------

```
Abstract 238 of 362

1. Comput Aided Surg. 2007 Mar;12(2):82-90.

Trajectory optimization for the planning of percutaneous radiofrequency ablation 
of hepatic tumors.

Baegert C, Villard C, Schreck P, Soler L, Gangi A.

LSIIT (UMR 7005 CNRS), Université Louis Pasteur Strasbourg I, Illkirch, France.

PMID: 17487658  [PubMed - indexed for MEDLINE]

Click here for link to PubMed for article 17487658
```

----------------------------

----------------------------

```
Abstract 239 of 362

1. Acad Med. 2007 May;82(5):446-51.

Virtual patient simulation at US and Canadian medical schools.

Huang G, Reynolds R, Candler C.

Office of Educational Technology, Carl J Shapiro Institute for Education and
Research at Harvard Medical School and Beth Israel Deaconess Medical Center,
Boston, Massachusetts 02215, USA.

PMID: 17457063  [PubMed - indexed for MEDLINE]

Click here for link to PubMed for article 17457063
```

----------------------------

----------------------------

```
Abstract 240 of 362

1. Int J Med Inform. 2007 May-Jun;76(5-6):331-5.

Virtual biomedical universities and e-learning.

Beux PL, Fieschi M.

PMID: 17407747  [PubMed - indexed for MEDLINE]

Click here for link to PubMed for article 17407747
```

----------------------------

----------------------------

```
Abstract 241 of 362

1. Stud Health Technol Inform. 2007;125:355-60.

A serious gaming/immersion environment to teach clinical cancer genetics.

Nosek TM, Cohen M, Matthews A, Papp K, Wolf N, Wrenn G, Sher A, Coulter K, Martin
J, Wiesner GL.

Case Western Reserve University School of Medicine, Cleveland, Ohio, USA.

PMID: 17377303  [PubMed - indexed for MEDLINE]

Click here for link to PubMed for article 17377303
```

----------------------------

----------------------------

```
Abstract 242 of 362

1. Stud Health Technol Inform. 2007;125:194-9.

An interactive, cognitive simulation of gastroesophageal reflux disease.

Jarrell B, Nirenburg S, McShane M, Fantry G, Beale S, Mallott D, Raczek J.

University of Maryland School of Medicine, MD, USA.

PMID: 17377265  [PubMed - indexed for MEDLINE]

Click here for link to PubMed for article 17377265
```

----------------------------

----------------------------

```
Abstract 243 of 362

1. Stud Health Technol Inform. 2007;125:185-90.

Localized virtual patient model for regional anesthesia simulation training
system.

Hu J, Lim YJ, Tardella N, Chang C, Warren L.

Energid Technologies Corporation, Cambridge, MA 02138, USA.

PMID: 17377263  [PubMed - indexed for MEDLINE]

Click here for link to PubMed for article 17377263
```

----------------------------

----------------------------

```
Abstract 244 of 362

1. Pediatrics. 2007 Mar;119(3):569-78.

Internet-based home monitoring and education of children with asthma is
comparable to ideal office-based care: results of a 1-year asthma in-home
monitoring trial.

Chan DS, Callahan CW, Hatch-Pigott VB, Lawless A, Proffitt HL, Manning NE,
Schweikert M, Malone FJ.

Department of Pediatrics, Tripler Army Medical Center, Honolulu, Hawaii, USA.

PMID: 17332210  [PubMed - indexed for MEDLINE]

Click here for link to PubMed for article 17332210
```

----------------------------

----------------------------

```
Abstract 245 of 362

1. Int J Med Inform. 2007 May-Jun;76(5-6):336-43. Epub 2007 Feb 28.

Training inter-physician communication using the Dynamic Patient Simulator.

Sijstermans R, Jaspers MW, Bloemendaal PM, Schoonderwaldt EM.

Department of Educational and Student Services, Academic Medical Center,
University of Amsterdam, PO Box 22700, 1100 AZ Amsterdam, The Netherlands.

PMID: 17331800  [PubMed - indexed for MEDLINE]

Click here for link to PubMed for article 17331800
```

----------------------------

----------------------------

```
Abstract 246 of 362

1. J Dent Educ. 2007 Feb;71(2):279-86.

Improving student dentist competencies and perception of difficulty in delivering
care to children with developmental disabilities using a virtual patient module.

Kleinert HL, Sanders C, Mink J, Nash D, Johnson J, Boyd S, Challman S.

Interdisciplinary Human Development Institute, University of Kentucky, Lexington,
KY 40506, USA.

PMID: 17314390  [PubMed - indexed for MEDLINE]

Click here for link to PubMed for article 17314390
```

----------------------------

----------------------------

```
Abstract 247 of 362

1. ANZ J Surg. 2007 Jan-Feb;77(1-2):54-9.

Virtual patients in undergraduate surgery education: a randomized controlled
study.

Vash JH, Yunesian M, Shariati M, Keshvari A, Harirchi I.

Academic Centre for Education, Culture and Research, Tehran University of Medical
Sciences, Tehran, Iran.

PMID: 17295822  [PubMed - indexed for MEDLINE]

Click here for link to PubMed for article 17295822
```

----------------------------

----------------------------

```
Abstract 248 of 362

1. Conf Proc IEEE Eng Med Biol Soc. 2004;3:1853-6.

A haptic-based system for medical image examination.

Abolmaesumi P, Hashtrudi-Zaad K, Thompson D, Tahmasebi A.

School of Computing, Queen's University, Kingston, Ontario, Canada.

PMID: 17272071  [PubMed]

Click here for link to PubMed for article 17272071
```

----------------------------

----------------------------

```
Abstract 249 of 362

1. Am J Pharm Educ. 2006 Apr 15;70(2):37.

Adaptive and longitudinal pharmaceutical care instruction using an interactive
voice response/text-to-speech system.

Hussein G, Kawahara N.

School of Pharmacy, Loma Linda University.

PMCID: PMC1636922
PMID: 17149416  [PubMed - indexed for MEDLINE]

Click here for link to PubMed for article 17149416
```

----------------------------

----------------------------

```
Abstract 250 of 362

1. Am J Pharm Educ. 2006 Apr 15;70(2):33.

Learning motivational interviewing: scripting a virtual patient.

Villaume WA, Berger BA, Barker BN.

Harrison School of Pharmacy, Auburn University.

PMCID: PMC1636931
PMID: 17149413  [PubMed - indexed for MEDLINE]

Click here for link to PubMed for article 17149413
```

----------------------------

----------------------------

```
Abstract 251 of 362

1. Stud Health Technol Inform. 2006;122:261-5.

Teaching undergraduate nursing students critical thinking: An innovative
informatics strategy.

Warren JJ, Connors HR, Weaver C, Simpson R.

University of Kansas School of Nursing, Kansas City KS, USA.

PMID: 17102261  [PubMed - indexed for MEDLINE]

Click here for link to PubMed for article 17102261
```

----------------------------

----------------------------

```
Abstract 252 of 362

1. J Pharmacokinet Pharmacodyn. 2006 Dec;33(6):773-94. Epub 2006 Oct 12.

Simulation of correlated continuous and categorical variables using a single
multivariate distribution.

Tannenbaum SJ, Holford NH, Lee H, Peck CC, Mould DR.

Novartis Pharmaceuticals Corp., One Health Plaza 435/1125, East Hanover, NJ
07936, USA.

PMID: 17053984  [PubMed - indexed for MEDLINE]

Click here for link to PubMed for article 17053984
```

----------------------------

----------------------------

```
Abstract 253 of 362

1. Obstet Gynecol. 2006 Oct;108(4):898-905.

Resident physicians' competencies and attitudes in delivering a postnatal
diagnosis of Down syndrome.

Ferguson JE 2nd, Kleinert HL, Lunney CA, Campbell LR.

Department of Obstetrics and Gynecology, Interdisciplinary Human Development
Institute, University of Kentucky and Chandler Medical Center, Lexington,
Kentucky 40536-0293, USA.

PMID: 17012452  [PubMed - indexed for MEDLINE]

Click here for link to PubMed for article 17012452
```

----------------------------

----------------------------

```
Abstract 254 of 362

1. Syst Biol (Stevenage). 2005 Dec;152(4):256-62.

Systems biology for battling rheumatoid arthritis: application of the Entelos
PhysioLab platform.

Rullmann JA, Struemper H, Defranoux NA, Ramanujan S, Meeuwisse CM, van Elsas A.

NV Organon, Oss, The Netherlands.

PMID: 16986268  [PubMed - indexed for MEDLINE]

Click here for link to PubMed for article 16986268
```

----------------------------

----------------------------

```
Abstract 255 of 362

1. Expert Opin Ther Targets. 2006 Oct;10(5):635-8.

Target validation in silico: does the virtual patient cure the pharma pipeline?

Alkema W, Rullmann T, van Elsas A.

PMID: 16981820  [PubMed - indexed for MEDLINE]

Click here for link to PubMed for article 16981820
```

----------------------------

----------------------------

```
Abstract 256 of 362

1. Clin Radiol. 2006 Aug;61(8):640-8.

Radiology education: a glimpse into the future.

Scarsbrook AF, Graham RN, Perriss RW.

Department of Radiology, John Radcliffe Hospital, Headley Way, Headington,
Oxford, UK.

PMID: 16843746  [PubMed - indexed for MEDLINE]

Click here for link to PubMed for article 16843746
```

----------------------------

----------------------------

```
Abstract 257 of 362

1. IEEE Trans Biomed Eng. 2006 Jun;53(6):996-1005.

Run-to-run control of blood glucose concentrations for people with Type 1
diabetes mellitus.

Owens C, Zisser H, Jovanovic L, Srinivasan B, Bonvin D, Doyle FJ 3rd.

Department of Chemical Engineering, University of Delaware, Newark 19716, USA.

PMID: 16761826  [PubMed - indexed for MEDLINE]

Click here for link to PubMed for article 16761826
```

----------------------------

----------------------------

```
Abstract 258 of 362

1. Diabetes Technol Ther. 2006 Apr;8(2):219-36.

Development and validation of the Economic Assessment of Glycemic Control and
Long-Term Effects of diabetes (EAGLE) model.

Mueller E, Maxion-Bergemann S, Gultyaev D, Walzer S, Freemantle N, Mathieu C,
Bolinder B, Gerber R, Kvasz M, Bergemann R.

Analytica International, Loerrach, Germany.

PMID: 16734551  [PubMed - indexed for MEDLINE]

Click here for link to PubMed for article 16734551
```

----------------------------

----------------------------

```
Abstract 259 of 362

1. Am J Surg. 2006 Jun;191(6):806-11.

The use of virtual patients to teach medical students history taking and
communication skills.

Stevens A, Hernandez J, Johnsen K, Dickerson R, Raij A, Harrison C, DiPietro M,
Allen B, Ferdig R, Foti S, Jackson J, Shin M, Cendan J, Watson R, Duerson M, Lok 
B, Cohen M, Wagner P, Lind DS.

College of Medicine, University of Florida, Gainesville, FL, USA.

PMID: 16720154  [PubMed - indexed for MEDLINE]

Click here for link to PubMed for article 16720154
```

----------------------------

----------------------------

```
Abstract 260 of 362

1. J Gen Intern Med. 2006 May;21(5):424-9.

A randomized trial of teaching clinical skills using virtual and live
standardized patients.

Triola M, Feldman H, Kalet AL, Zabar S, Kachur EK, Gillespie C, Anderson M,
Griesser C, Lipkin M.

NYU School of Medicine, New York, NY 10016, USA.

PMCID: PMC1484797
PMID: 16704382  [PubMed - indexed for MEDLINE]

Click here for link to PubMed for article 16704382
```

----------------------------

----------------------------

```
Abstract 261 of 362

1. Comput Methods Programs Biomed. 2006 Jun;82(3):238-47. Epub 2006 May 2.

Integral-based filtering of continuous glucose sensor measurements for glycaemic 
control in critical care.

Chase JG, Hann CE, Jackson M, Lin J, Lotz T, Wong XW, Shaw GM.

Department of Mechanical Engineering, Centre for Bio-Engineering, University of
Canterbury, Private Bag 4800, Christchurch, New Zealand.

PMID: 16647157  [PubMed - indexed for MEDLINE]

Click here for link to PubMed for article 16647157
```

----------------------------

----------------------------

```
Abstract 262 of 362

1. Arch Intern Med. 2006 Mar 27;166(6):610-6.

Competency in cardiac examination skills in medical students, trainees,
physicians, and faculty: a multicenter study.

Vukanovic-Criley JM, Criley S, Warde CM, Boker JR, Guevara-Matheus L, Churchill
WH, Nelson WP, Criley JM.

Stanford University School of Medicine, CA, USA.

Erratum in
    Arch Intern Med. 2006 Jun 26;166(12):1294.

PMID: 16567598  [PubMed - indexed for MEDLINE]

Click here for link to PubMed for article 16567598
```

----------------------------

----------------------------

```
Abstract 263 of 362

1. BMC Med Educ. 2006 Feb 21;6:10.

Development, implementation and pilot evaluation of a Web-based Virtual Patient
Case Simulation environment--Web-SP.

Zary N, Johnson G, Boberg J, Fors UG.

Dept of Learning, Informatics, Management and Ethics, Karolinska Institutet, 171 
77 Stockholm, Sweden.

PMCID: PMC1397827
PMID: 16504041  [PubMed - indexed for MEDLINE]

Click here for link to PubMed for article 16504041
```

----------------------------

----------------------------

```
Abstract 264 of 362

1. Diabetes Technol Ther. 2006 Feb;8(1):126-37.

Interactive educational diabetes/insulin tutorial at www.2aida.info.

Reed K, Lehmann ED.

Reed Biomedical, Rotorua, New Zealand.

PMID: 16472060  [PubMed - indexed for MEDLINE]

Click here for link to PubMed for article 16472060
```

----------------------------

----------------------------

```
Abstract 265 of 362

1. IEEE Trans Inf Technol Biomed. 2006 Jan;10(1):28-41.

Intelligent inferencing and haptic simulation for Chinese acupuncture learning
and training.

Heng PA, Wong TT, Yang R, Chui YP, Xie YM, Leung KS, Leung PC.

Department of Computer Science and Engineering, The Chinese University of Hong
Kong.

PMID: 16445247  [PubMed - indexed for MEDLINE]

Click here for link to PubMed for article 16445247
```

----------------------------

----------------------------

```
Abstract 266 of 362

1. Stud Health Technol Inform. 2006;119:114-9.

Virtual patients: assessment of synthesized versus recorded speech.

Dickerson R, Johnsen K, Raij A, Lok B, Stevens A, Bernard T, Lind DS.

Department of Computer Information Science Engineering, University of Florida,
USA.

PMID: 16404028  [PubMed - indexed for MEDLINE]

Click here for link to PubMed for article 16404028
```

----------------------------

----------------------------

```
Abstract 267 of 362

1. Eur J Vasc Endovasc Surg. 2006 Jun;31(6):588-93. Epub 2006 Jan 4.

Virtual reality simulation training can improve inexperienced surgeons'
endovascular skills.

Aggarwal R, Black SA, Hance JR, Darzi A, Cheshire NJ.

Department of Biosurgery and Surgical Technology, Imperial College London,
London, UK.

Comment in
    Eur J Vasc Endovasc Surg. 2007 Feb;33(2):259; author reply 260.

PMID: 16387517  [PubMed - indexed for MEDLINE]

Click here for link to PubMed for article 16387517
```

----------------------------

----------------------------

```
Abstract 268 of 362

1. Dement Geriatr Cogn Disord. 2006;21(2):97-103. Epub 2005 Dec 12.

Reliability study on the Japanese version of the Clinician's Interview-Based
Impression of Change.

Homma A, Nakamura Y, Kobune S, Haraguchi H, Kodani N, Takami I, Matsuoka J,
Matsuda H, Kusunoki T.

Tokyo Metropolitan Institute of Gerontology, Tokyo, Japan.

PMID: 16352896  [PubMed - indexed for MEDLINE]

Click here for link to PubMed for article 16352896
```

----------------------------

----------------------------

```
Abstract 269 of 362

1. Med Educ. 2005 Nov;39(11):1153-4.

Virtual patients get real.

Walsh K.

PMID: 16262825  [PubMed - indexed for MEDLINE]

Click here for link to PubMed for article 16262825
```

----------------------------

----------------------------

```
Abstract 270 of 362

1. Ann Acad Med Singapore. 2005 Sep;34(8):499-504.

An intelligent tutoring system for trauma management (Trauma-Teach): a
preliminary report.

Ong LS, Vijayan A, Koh CS, Lai CC, Lim CW, Loke WF, Low SH, Tang KY, Wong FL,
Yong KL.

Institute of Systems Science, National University of Singapore, 25 Heng Mui Keng 
Terrace, Singapore 119615.

PMID: 16205828  [PubMed - indexed for MEDLINE]

Click here for link to PubMed for article 16205828
```

----------------------------

----------------------------

```
Abstract 271 of 362

1. Adv Health Sci Educ Theory Pract. 2005 Aug;10(3):215-30.

Evaluation of an Interactive Case-based Online Network (ICON) in a problem based 
learning environment.

Nathoo AN, Goldhoff P, Quattrochi JJ.

Francis Weld Peabody Society, Harvard Medical School, Tosteson Medical Education 
Center, 260 Longwood Ave, Suite 255, Boston, MA 02115, USA.

PMID: 16193402  [PubMed - indexed for MEDLINE]

Click here for link to PubMed for article 16193402
```

----------------------------

----------------------------

```
Abstract 272 of 362

1. J Contin Educ Health Prof. 2005 Winter;25(1):43-51.

A new vision for distance learning and continuing medical education.

Harden RM.

IVIMEDS, Dundee, Scotland, United Kingdom.

PMID: 16078802  [PubMed - indexed for MEDLINE]

Click here for link to PubMed for article 16078802
```

----------------------------

----------------------------

```
Abstract 273 of 362

1. Int J Med Inform. 2005 Aug;74(7-8):605-13. Epub 2005 Apr 9.

Using OrgAhead, a computational modeling program, to improve patient care unit
safety and quality outcomes.

Effken JA, Brewer BB, Patil A, Lamb GS, Verran JA, Carley K.

University of Arizona College of Nursing, P.O. Box 210203, Tucson, AZ 85721-0203,
USA.

PMID: 16043085  [PubMed - indexed for MEDLINE]

Click here for link to PubMed for article 16043085
```

----------------------------

----------------------------

```
Abstract 274 of 362

1. J Med Eng Technol. 2005 Jul-Aug;29(4):170-5.

Unipolar cardiac pacemakers in electromagnetic fields of high voltage overhead
lines.

Scholten A, Joosten S, Silny J.

FEMU, University Hospital, RWTH Aachen, Germany.

PMID: 16012068  [PubMed - indexed for MEDLINE]

Click here for link to PubMed for article 16012068
```

----------------------------

----------------------------

```
Abstract 275 of 362

1. J Craniomaxillofac Surg. 2005 Aug;33(4):223-8.

A surgical simulator for planning and performing repair of cleft lips.

Schendel S, Montgomery K, Sorokin A, Lionetti G.

National Biocomputation Center, Stanford University, CA 94305, USA.

PMID: 15975810  [PubMed - indexed for MEDLINE]

Click here for link to PubMed for article 15975810
```

----------------------------

----------------------------

```
Abstract 276 of 362

1. Med Eng Phys. 2006 Mar;28(2):134-48. Epub 2005 Jun 14.

Genetic fuzzy modelling and control of bispectral index (BIS) for general
intravenous anaesthesia.

Shieh JS, Kao MH, Liu CC.

Department of Mechanical Engineering, Yuan Ze University, 135 Yuan-Tung Rd.,
Chung-Li, Taoyuan 320, Taiwan.

PMID: 15961340  [PubMed - indexed for MEDLINE]

Click here for link to PubMed for article 15961340
```

----------------------------

----------------------------

```
Abstract 277 of 362

1. Comput Med Imaging Graph. 2005 Jul;29(5):385-94.

An integrated environment for plastic surgery support: building virtual patients,
simulating interventions, and supporting intraoperative decisions.

Porro I, Schenone A, Fato M, Raposio E, Molinari E, Beltrame F.

DIST Department of Communication, Computer and System Sciences, University of
Genova, 16145 Genova, Italy.

PMID: 15893913  [PubMed - indexed for MEDLINE]

Click here for link to PubMed for article 15893913
```

----------------------------

----------------------------

```
Abstract 278 of 362

1. Stud Health Technol Inform. 2004;107(Pt 2):983-7.

A problem-based e-Learning prototype system for clinical medical education.

Shyu FM, Liang YF, Hsu WT, Luh JJ, Chen HS.

Institute of Electrical Engineering and Department of Information System of
National Taiwan University and Hospital.

PMID: 15871176  [PubMed - indexed for MEDLINE]

Click here for link to PubMed for article 15871176
```

----------------------------

----------------------------

```
Abstract 279 of 362

1. Yonsei Med J. 2005 Apr 30;46(2):275-83.

Computational analysis of tumor angiogenesis patterns using a two-dimensional
model.

Shim EB, Kwon YG, Ko HJ.

Department of Mechanical and Biomedical Engineering, Kangwon National University,
Hyoja-dong, Chuncheon, Kangwon-do 200-701, Korea.

PMCID: PMC2823025
PMID: 15861502  [PubMed - indexed for MEDLINE]

Click here for link to PubMed for article 15861502
```

----------------------------

----------------------------

```
Abstract 280 of 362

1. Hosp Health Netw. 2005 Jan;79(1):52-6, 2.

High-tech tools transform the operating room.

Haugh R.

PMID: 15720026  [PubMed - indexed for MEDLINE]

Click here for link to PubMed for article 15720026
```

----------------------------

----------------------------

```
Abstract 281 of 362

1. Stud Health Technol Inform. 2005;111:37-42.

Predictive biosimulation and virtual patients in pharmaceutical R and D.

Bangs A.

Entelos, Inc., 110 Marsh Dr., Foster City, CA 94404, USA.

PMID: 15718695  [PubMed - indexed for MEDLINE]

Click here for link to PubMed for article 15718695
```

----------------------------

----------------------------

```
Abstract 282 of 362

1. Z Orthop Ihre Grenzgeb. 2004 Nov-Dec;142(6):659-65.

[Assessment of implant position of CTX-custom-made stems with EBRA-FCA in 107
cases of total joint replacement].

[Article in German]

Rittmeister M, Eisenbeis K, Hanusek S, Yanik-Karaca Z, Starker M, Arabmotlagh M.

Orthopädische Universitätsklinik, Friedrichsheim, Johann Wolfgang
Goethe-Universität, Frankfurt am Main.

PMID: 15614644  [PubMed - indexed for MEDLINE]

Click here for link to PubMed for article 15614644
```

----------------------------

----------------------------

```
Abstract 283 of 362

1. Stud Health Technol Inform. 2004;98:353-9.

Virtual patients in clinical medicine.

Simo A, Cavazza M, Kijima R.

Virtual Systems Laboratory, University of Gifu 1-1 Yanagido, Gifu-shi, Gifu,
501-1193, Japan.

PMID: 15544304  [PubMed - indexed for MEDLINE]

Click here for link to PubMed for article 15544304
```

----------------------------

----------------------------

```
Abstract 284 of 362

1. Stud Health Technol Inform. 2004;98:154-6.

Virtual patient: a photo-real virtual human for VR-based therapy.

Kiss B, Benedek B, Szijártó G, Csukly G, Simon L, Takács B.

VerAnim, Budapest, Hungary.

PMID: 15544262  [PubMed - indexed for MEDLINE]

Click here for link to PubMed for article 15544262
```

----------------------------

----------------------------

```
Abstract 285 of 362

1. Biotechnol Annu Rev. 2004;10:297-302.

Biosimulation software is changing research.

Ho RL, Bartsell LT.

Johnson & Johnson Pharmaceutical Research & Development, L.L.C., La Jolla, CA,
USA.

PMID: 15504712  [PubMed - indexed for MEDLINE]

Click here for link to PubMed for article 15504712
```

----------------------------

----------------------------

```
Abstract 286 of 362

1. Stud Health Technol Inform. 2002;90:298-304.

Developing a virtual patient record as a web-based workflow system.

Malamateniou F, Vassilacopoulos G.

Department of Informatics, University of Piraeus, GR-Piraeus 18534, Greece.

PMID: 15460706  [PubMed - indexed for MEDLINE]

Click here for link to PubMed for article 15460706
```

----------------------------

----------------------------

```
Abstract 287 of 362

1. Stud Health Technol Inform. 2002;85:221-7.

A virtual environment for esophageal intubation training.

Kesavadas T, Joshi D, Mayrose J, Chugh K.

Virtual Reality Lab, Department of Mechanical and Aerospace Engineering, State
University of New York at Buffalo, Buffalo, NY 14260, USA.

PMID: 15458090  [PubMed - indexed for MEDLINE]

Click here for link to PubMed for article 15458090
```

----------------------------

----------------------------

```
Abstract 288 of 362

1. Stud Health Technol Inform. 2003;94:204-9.

A surgical simulator for cleft lip planning and repair.

Montgomery K, Sorokin A, Lionetti G, Schendel S.

National Biocomputation Center, Stanford University, USA.

PMID: 15455894  [PubMed - indexed for MEDLINE]

Click here for link to PubMed for article 15455894
```

----------------------------

----------------------------

```
Abstract 289 of 362

1. Stud Health Technol Inform. 2003;94:100-2.

Trauma team training in a distributed virtual emergency room.

Halvorsrud R, Hagen S, Fagernes S, Mjelstad S, Romundstad L.

Telenor Research and Development, N-1331 Fornebu, Norway.

PMID: 15455872  [PubMed - indexed for MEDLINE]

Click here for link to PubMed for article 15455872
```

----------------------------

----------------------------

```
Abstract 290 of 362

1. Cancer J. 2004 Jul-Aug;10(4):214-20.

Functional anatomic imaging in radiation therapy planning.

Brunetti J, Caggiano A, Vialotti C.

Department of Radiology, Holy Name Hospital, Teaneck, New Jersey 07960, USA.

PMID: 15383202  [PubMed - indexed for MEDLINE]

Click here for link to PubMed for article 15383202
```

----------------------------

----------------------------

```
Abstract 291 of 362

1. Stud Health Technol Inform. 2004;107(Pt 2):1048-52.

Design of a patient-centered, multi-institutional healthcare information network 
using peer-to-peer communication in a highly distributed architecture.

Geissbuhler A, Spahni S, Assimacopoulos A, Raetzo MA, Gobet G.

Service d'Informatique Médicale, Hôpitaux universitaires de Genève, Switzerland. 

PMID: 15360972  [PubMed - indexed for MEDLINE]

Click here for link to PubMed for article 15360972
```

----------------------------

----------------------------

```
Abstract 292 of 362

1. Stud Health Technol Inform. 2004;107(Pt 2):921-5.

CAMPUS--a flexible, interactive system for web-based, problem-based learning in
health care.

Ruderich F, Bauch M, Haag M, Heid J, Leven FJ, Singer R, Geiss HK, Jünger J,
Tönshoff B.

Laboratory for Computer-based Teaching and Learning Systems in Medicine,
University of Heidelberg, Germany.

PMID: 15360947  [PubMed - indexed for MEDLINE]

Click here for link to PubMed for article 15360947
```

----------------------------

----------------------------

```
Abstract 293 of 362

1. Stud Health Technol Inform. 2004;107(Pt 1):726-30.

Using computational modeling to improve patient care unit safety and quality
outcomes.

Effken JA, Brewer BB, Patil A, Lamb GS, Verran JA, Carley K.

University of Arizona College of Nursing, PO Box 210203, Tucson, AZ 85721, USA.

PMID: 15360908  [PubMed - indexed for MEDLINE]

Click here for link to PubMed for article 15360908
```

----------------------------

----------------------------

```
Abstract 294 of 362

1. Ultrasound Obstet Gynecol. 2004 Sep;24(4):440-4.

Ultrasound training: the virtual patient.

Heer IM, Middendorf K, Müller-Egloff S, Dugas M, Strauss A.

Department of Obstetrics and Gynecology-Grosshadern, University Hospital,
University of Munich, Munich, Germany.

Copyright 2004 ISUOG

PMID: 15343601  [PubMed - indexed for MEDLINE]

Click here for link to PubMed for article 15343601
```

----------------------------

----------------------------

```
Abstract 295 of 362

1. Manag Care Interface. 2004 Jul;17(7):50-3.

Interdisciplinary management of chronic disease in primary practice.

Lapidos S, Rothschild SK.

Virtual Integrated Practice Project, Department of Preventive Medicine, Rush
University Medical Center, Chicago, Illinois 60612, USA.

PMID: 15341248  [PubMed - indexed for MEDLINE]

Click here for link to PubMed for article 15341248
```

----------------------------

----------------------------

```
Abstract 296 of 362

1. Eur J Dent Educ. 2004 Aug;8(3):111-9.

Simulation of patient encounters using a virtual patient in periodontology
instruction of dental students: design, usability, and learning effect in
history-taking skills.

Schittek Janda M, Mattheos N, Nattestad A, Wagner A, Nebel D, Färbom C, Lê DH,
Attström R.

Department of Periodontology, Centre for Oral Health Sciences, Malmö University, 
Malmo, Sweden.

PMID: 15233775  [PubMed - indexed for MEDLINE]

Click here for link to PubMed for article 15233775
```

----------------------------

----------------------------

```
Abstract 297 of 362

1. Physician Exec. 2004 May-Jun;30(3):32-4.

The virtual patient encounter--units of service in the electronic age.

Marco AP.

Department of Anesthesiology, Medical College of Ohio, Toledo, Ohio, USA.

PMID: 15179888  [PubMed - indexed for MEDLINE]

Click here for link to PubMed for article 15179888
```

----------------------------

----------------------------

```
Abstract 298 of 362

1. Anesthesiology. 2004 Mar;100(3):640-7.

Performance evaluation of two published closed-loop control systems using
bispectral index monitoring: a simulation study.

Struys MM, De Smet T, Greenwald S, Absalom AR, Bingé S, Mortier EP.

Department of Anesthesia, Ghent University Hospital, Gent, Belgium.

PMID: 15108980  [PubMed - indexed for MEDLINE]

Click here for link to PubMed for article 15108980
```

----------------------------

----------------------------

```
Abstract 299 of 362

1. Stud Health Technol Inform. 2003;95:158-63.

An implementation of a virtual patient record using Web services.

Malamateniou F, Vassilacopoulos G.

Computer Technology Institute, Athens 118 51, Greece.

PMID: 14663980  [PubMed - indexed for MEDLINE]

Click here for link to PubMed for article 14663980
```

----------------------------

----------------------------

```
Abstract 300 of 362

1. Resuscitation. 2003 Oct;59(1):133-8.

Utilization of virtual reality for endotracheal intubation training.

Mayrose J, Kesavadas T, Chugh K, Joshi D, Ellis DG.

Department of Emergency Medicine, State University of New York at Buffalo, Erie
County Medical Center, 462 Grider Street, Buffalo, NY 14215, USA.

PMID: 14580744  [PubMed - indexed for MEDLINE]

Click here for link to PubMed for article 14580744
```

----------------------------

----------------------------

```
Abstract 301 of 362

1. JAMA. 2003 Oct 1;290(13):1700-1.

Virtual patients help medical students link basic science with clinical care.

Voelker R.

PMID: 14519694  [PubMed - indexed for MEDLINE]

Click here for link to PubMed for article 14519694
```

----------------------------

----------------------------

```
Abstract 302 of 362

1. Int J Med Inform. 2003 Jul;70(2-3):131-9.

Developing a virtual patient record using XML and web-based workflow
technologies.

Malamateniou F, Vassilacopoulos G.

Computer Technology Institute, Athens 118 51, Greece.

PMID: 12909164  [PubMed - indexed for MEDLINE]

Click here for link to PubMed for article 12909164
```

----------------------------

----------------------------

```
Abstract 303 of 362

1. Ugeskr Laeger. 2003 May 5;165(19):1961-2.

[New drugs are tested on virtual patients. It will be possible to test new drugs 
on virtual patients, existing only in a computer].

[Article in Danish]

Andreasen J.

PMID: 12795067  [PubMed - indexed for MEDLINE]

Click here for link to PubMed for article 12795067
```

----------------------------

----------------------------

```
Abstract 304 of 362

1. WMJ. 2003;102(2):14-7.

The Medical College of Wisconsin's program to strengthen geriatrics education.

Burns E, Bates T, Cohan M, Kowalski K, Olds GR, Simpson D, Duthie EH Jr.

Division of Geriatrics and Gerontology, Medical College of Wisconsin, USA.

PMID: 12754902  [PubMed - indexed for MEDLINE]

Click here for link to PubMed for article 12754902
```

----------------------------

----------------------------

```
Abstract 305 of 362

1. Acad Med. 2003 May;78(5):538-45.

Is virtual the same as real? Medical students' experiences of a virtual patient.

Bearman M.

Monash Institute of Health Services Research, Monash University, Clayton
Victoria, Australia.

PMID: 12742794  [PubMed - indexed for MEDLINE]

Click here for link to PubMed for article 12742794
```

----------------------------

----------------------------

```
Abstract 306 of 362

1. Int Arch Occup Environ Health. 2003 Feb;76(1):50-4. Epub 2002 Sep 4.

Web-based training in occupational medicine.

Hege I, Radon K, Dugas M, Scharrer E, Nowak D.

Institute of Occupational and Environmental Medicine,
Ludwig-Maximilians-University, Ziemssenstrasse 1, 80336 Munich, Germany.

PMID: 12592582  [PubMed - indexed for MEDLINE]

Click here for link to PubMed for article 12592582
```

----------------------------

----------------------------

```
Abstract 307 of 362

1. Sci Am. 2003 Feb;288(2):18-9.

Reverse-engineering clinical biology. A peacetime dividend yields drug trials on 
virtual patients.

Stix G.

PMID: 12561454  [PubMed - indexed for MEDLINE]

Click here for link to PubMed for article 12561454
```

----------------------------

----------------------------

```
Abstract 308 of 362

1. Anat Rec B New Anat. 2003 Jan;270(1):23-9.

Virtual patient simulator for distributed collaborative medical education.

Caudell TP, Summers KL, Holten J 4th, Hakamata T, Mowafi M, Jacobs J, Lozanoff
BK, Lozanoff S, Wilks D, Keep MF, Saiki S, Alverson D.

Department of Electrical and Computer Engineering, University of New Mexico,
Albuquerque 87131, USA.

Copyright 2003 Wiley-Liss, Inc.

PMID: 12526063  [PubMed - indexed for MEDLINE]

Click here for link to PubMed for article 12526063
```

----------------------------

----------------------------

```
Abstract 309 of 362

1. Biomed Tech (Berl). 2002;47 Suppl 1 Pt 1:121-3.

[Cathi-training on virtual patients for catheter interventions].

[Article in German]

Kornmesser U, Hesser J, Voelker W, Männer R.

Institut für computerunterstützte Medizin, Universität Mannheim, Deutschland.

PMID: 12451790  [PubMed - indexed for MEDLINE]

Click here for link to PubMed for article 12451790
```

----------------------------

----------------------------

```
Abstract 310 of 362

1. Acad Med. 2002 Jul;77(7):750-1.

Colonoscopy curriculum development and performance-based assessment criteria on a
computer-based endoscopy simulator.

Sedlack RE, Kolars JC.

Mayo Clinic, Rochester, MN 55905, USA.

PMID: 12114172  [PubMed - indexed for MEDLINE]

Click here for link to PubMed for article 12114172
```

----------------------------

----------------------------

```
Abstract 311 of 362

1. Telemed J E Health. 2002 Spring;8(1):131-7.

Telemedicine to integrate intermittent surgical services into primary care.

Doarn CR, Fitzgerald S, Rodas E, Harnett B, Prabe-Egge A, Merrell RC.

Medical Informatics and Technology Applications Consortium, Virginia Commonwealth
University, Richmond, Virginia 23298, USA.


PMID: 12020413  [PubMed - indexed for MEDLINE]

Click here for link to PubMed for article 12020413
```

----------------------------

----------------------------

```
Abstract 312 of 362

1. Swiss Surg. 2002;8(2):67-73.

The effect of virtual reality and training on liver operation planning.

Herfarth C, Lamadé W, Fischer L, Chiu P, Cardenas C, Thorn M, Vetter M, Grenacher
L, Meinzer HP.

Department of Surgery, University of Heidelberg, Germany.

PMID: 12013693  [PubMed - indexed for MEDLINE]

Click here for link to PubMed for article 12013693
```

----------------------------

----------------------------

```
Abstract 313 of 362

1. Comput Biol Med. 2002 Mar;32(2):73-83.

An approach to computer automation of the extracorporeal circulation.

Boschetti F, Mantero S, Miglietta F, Costantino ML, Montevecchi FM, Fumero R.

Dipartimento di Bioingegneria, Politecnico di Milano, Piazza. L da Vinci 32,
20133 Milano, Italy.

PMID: 11879821  [PubMed - indexed for MEDLINE]

Click here for link to PubMed for article 11879821
```

----------------------------

----------------------------

```
Abstract 314 of 362

1. BJU Int. 2002 Feb;89(3):174-7.

The URO Mentor: development and evaluation of a new computer-based interactive
training system for virtual life-like simulation of diagnostic and therapeutic
endourological procedures.

Michel MS, Knoll T, Köhrmann KU, Alken P.

Department of Urology, University Hospital Mannheim, Germany.

PMID: 11856093  [PubMed - indexed for MEDLINE]

Click here for link to PubMed for article 11856093
```

----------------------------

----------------------------

```
Abstract 315 of 362

1. Proc AMIA Symp. 2001:244-8.

Virtual healthcare delivery: defined, modeled, and predictive barriers to
implementation identified.

Harrop VM.

MIT, USA.

PMCID: PMC2243512
PMID: 11825189  [PubMed - indexed for MEDLINE]

Click here for link to PubMed for article 11825189
```

----------------------------

----------------------------

```
Abstract 316 of 362

1. Stud Health Technol Inform. 2001;84(Pt 2):1042-6.

GOLEM--multimedia simulator for medical education.

Kofránek J, Vu LD, Snáselová H, Kerekes R, Velan T.

Department of Pathological Physiology, 1st Medical Faculty, Charles University,
Prague, Czech Republic.

PMID: 11604890  [PubMed - indexed for MEDLINE]

Click here for link to PubMed for article 11604890
```

----------------------------

----------------------------

```
Abstract 317 of 362

1. Stud Health Technol Inform. 2001;84(Pt 2):1004-8.

Comparing student attitudes to different models of the same virtual patient.

Bearman M, Cesnik B.

Centre of Medical Informatics, Monash University, Melbourne, Australia.

PMID: 11604882  [PubMed - indexed for MEDLINE]

Click here for link to PubMed for article 11604882
```

----------------------------

----------------------------

```
Abstract 318 of 362

1. Ultrasound Med Biol. 2001 Sep;27(9):1239-43.

Telemedicine in ultrasound: new solutions.

Heer IM, Strauss A, Müller-Egloff S, Hasbargen U.

Ludwig Maximilians University Munich, Klinikum Grosshadern, Department of
Obstetrics and Gynecology, Munich, Germany.

PMID: 11597365  [PubMed - indexed for MEDLINE]

Click here for link to PubMed for article 11597365
```

----------------------------

----------------------------

```
Abstract 319 of 362

1. Comput Aided Surg. 2001;6(2):85-93.

A full 3D-navigation system in a suitcase.

Freysinger W, Truppe MJ, Gunkel AR, Thumfart WF.

4D Visualization Laboratory, ENT Clinic, University of Innsbruck, Austria.

Copyright 2001 Wiley-Liss, Inc.

PMID: 11568984  [PubMed - indexed for MEDLINE]

Click here for link to PubMed for article 11568984
```

----------------------------

----------------------------

```
Abstract 320 of 362

1. Med Educ. 2001 Sep;35(9):824-32.

Random comparison of 'virtual patient' models in the context of teaching clinical
communication skills.

Bearman M, Cesnik B, Liddell M.

Centre of Medical Informatics, Monash University, Institute of Public Health,
Clayton, Victoria, Australia.

PMID: 11555219  [PubMed - indexed for MEDLINE]

Click here for link to PubMed for article 11555219
```

----------------------------

----------------------------

```
Abstract 321 of 362

1. J Ultrasound Med. 2001 Sep;20(9):941-52.

Feasibility of performing a virtual patient examination using three-dimensional
ultrasonographic data acquired at remote locations.

Nelson TR, Pretorius DH, Lev-Toaff A, Bega G, Budorick NE, Hollenbach KA,
Needleman L.

Department of Radiology, University of California, San Diego, La Jolla 92014,
USA.

PMID: 11549153  [PubMed - indexed for MEDLINE]

Click here for link to PubMed for article 11549153
```

----------------------------

----------------------------

```
Abstract 322 of 362

1. Eur J Dent Educ. 2001 Aug;5(3):93-100.

Computer assisted learning. A review.

Schittek M, Mattheos N, Lyon HC, Attström R.

Department of Periodontology, Centre for Oral Health Sciences, Malmö University, 
Carl Gustavs Väg 34, 214 21 Malmö, Sweden.

PMID: 11520331  [PubMed - indexed for MEDLINE]

Click here for link to PubMed for article 11520331
```

----------------------------

----------------------------

```
Abstract 323 of 362

1. Stroke. 2001 Aug;32(8):1800-7.

Development of a novel, weighted, quantifiable stroke scale: Japan stroke scale.

Gotoh F, Terayama Y, Amano T; Stroke Scale Committee of the Japan Stroke Society.

Department of Neurology, School of Medicine, Keio University, Tokyo, Japan.

Erratum in
    Stroke 2002 Apr;33(4):1171.

PMID: 11486108  [PubMed - indexed for MEDLINE]

Click here for link to PubMed for article 11486108
```

----------------------------

----------------------------

```
Abstract 324 of 362

1. Vet J. 2001 Jul;162(1):1-2.

The virtual patient -- addressing the placebo challenge.

Higgins A.

PMID: 11409921  [PubMed - indexed for MEDLINE]

Click here for link to PubMed for article 11409921
```

----------------------------

----------------------------

```
Abstract 325 of 362

1. Prehosp Disaster Med. 2001 Jan-Mar;16(1):3-8.

A virtual reality patient simulation system for teaching emergency response
skills to U.S. Navy medical providers.

Freeman KM, Thompson SF, Allely EB, Sobel AL, Stansfield SA, Pugh WM.

Naval Health Research Center, P.O. Box 85122, San Diego, California, 92186-5122, 
USA.

PMID: 11367936  [PubMed - indexed for MEDLINE]

Click here for link to PubMed for article 11367936
```

----------------------------

----------------------------

```
Abstract 326 of 362

1. Med Educ. 2001 May;35(5):505-9.

Time to learn: the outlook for renewal of patient-centred education in the
digital age.

Glick TH, Moore GT.

Department of Medicine, The Cambridge Health Alliance and the Department of
Neurology, Harvard Medical School, Cambridge, MA, USA.

PMID: 11328522  [PubMed - indexed for MEDLINE]

Click here for link to PubMed for article 11328522
```

----------------------------

----------------------------

```
Abstract 327 of 362

1. Int J Adult Orthodon Orthognath Surg. 2000 Winter;15(4):265-82.

Three-dimensional virtual reality surgical planning and simulation workbench for 
orthognathic surgery.

Xia J, Samman N, Yeung RW, Shen SG, Wang D, Ip HH, Tideman H.

Oral and Maxillofacial Surgery, University of Hong Kong.

PMID: 11307184  [PubMed - indexed for MEDLINE]

Click here for link to PubMed for article 11307184
```

----------------------------

----------------------------

```
Abstract 328 of 362

1. Arch Ophthalmol. 2000 Dec;118(12):1679-81.

Vitreous surgery simulator.

Hikichi T, Yoshida A, Igarashi S, Mukai N, Harada M, Muroi K, Terada T.

Department of Ophthalmology, Asahikawa Medical College, 2-1 Midorigaoka-higashi, 
Asahikawa 078-8307, Japan.

PMID: 11115263  [PubMed - indexed for MEDLINE]

Click here for link to PubMed for article 11115263
```

----------------------------

----------------------------

```
Abstract 329 of 362

1. Curr Probl Cardiol. 2000 Nov;25(11):783-825.

Bedside cardiac examination: constancy in a sea of change.

Richardson TR, Moody JM Jr.

Division of Cardiology, University of Texas Health Science Center, San Antonio,
USA.

PMID: 11082789  [PubMed - indexed for MEDLINE]

Click here for link to PubMed for article 11082789
```

----------------------------

----------------------------

```
Abstract 330 of 362

1. Arch Surg. 2000 Nov;135(11):1256-61.

The impact of 3-dimensional reconstructions on operation planning in liver
surgery.

Lamadé W, Glombitza G, Fischer L, Chiu P, Cárdenas CE Sr, Thorn M, Meinzer HP,
Grenacher L, Bauer H, Lehnert T, Herfarth C.

Department of Surgery, University of Heidelberg, Im Neuenheimer Feld 110, 69120
Heidelberg, Germany.

PMID: 11074877  [PubMed - indexed for MEDLINE]

Click here for link to PubMed for article 11074877
```

----------------------------

----------------------------

```
Abstract 331 of 362

1. Stud Health Technol Inform. 2000;70:316-22.

An automatic virtual patient reconstruction from CT-scans for hepatic surgical
planning.

Soler L, Delingette H, Malandain G, Ayache N, Koehl C, Clément JM, Dourthe O,
Marescaux J.

IRCAD Strasbourg, France.

PMID: 10977563  [PubMed - indexed for MEDLINE]

Click here for link to PubMed for article 10977563
```

----------------------------

----------------------------

```
Abstract 332 of 362

1. Mund Kiefer Gesichtschir. 2000 May;4 Suppl 1:S369-74.

[Telenavigation and expert consultation using a stereotaxic surgical
videoserver].

[Article in German]

Wagner A, Kremser J, Watzinger F, Friede I, Truppe M, Ewers R.

Universitätsklinik für Mund- Kiefer- Gesichtschirurgie, AKH Wien.

PMID: 10938679  [PubMed - indexed for MEDLINE]

Click here for link to PubMed for article 10938679
```

----------------------------

----------------------------

```
Abstract 333 of 362

1. Med Arh. 1999;53(3 Suppl 3):25-7.

[Expert systems in medicine].

[Article in Croatian]

Pandza H, Masić I.

Centar za medicinsku informatiku, Medicinski fakultet Univerziteta u Sarajevu.

PMID: 10870619  [PubMed - indexed for MEDLINE]

Click here for link to PubMed for article 10870619
```

----------------------------

----------------------------

```
Abstract 334 of 362

1. J Telemed Telecare. 2000;6(2):119-20.

Theory of telemedicine: the expressivity of the virtual patient.

Hjelm NM, Hazlett CB.

Comment in
    J Telemed Telecare. 2000;6(5):304-5.

PMID: 10824381  [PubMed - indexed for MEDLINE]

Click here for link to PubMed for article 10824381
```

----------------------------

----------------------------

```
Abstract 335 of 362

1. Radiologe. 2000 Mar;40(3):211-7.

[Virtual reality in neurosurgery].

[Article in German]

Tronnier VM, Staubert A, Bonsanto MM, Wirtz CR, Kunze S.

Neurochirurgische Universitätsklinik Heidelberg.

PMID: 10789118  [PubMed - indexed for MEDLINE]

Click here for link to PubMed for article 10789118
```

----------------------------

----------------------------

```
Abstract 336 of 362

1. IEEE Trans Inf Technol Biomed. 1998 Sep;2(3):139-45.

A workflow-based approach to virtual patient record security.

Malamateniou F, Vassilacopoulos G, Tsanakas P.

Department of Informatics, University of Piraeus, Greece.

PMID: 10719523  [PubMed - indexed for MEDLINE]

Click here for link to PubMed for article 10719523
```

----------------------------

----------------------------

```
Abstract 337 of 362

1. Int J Med Inform. 1999 Aug;55(2):103-15.

A search engine for virtual patient records.

Malamateniou F, Vassilacopoulos G, Mantas J.

Department of Informatics, University of Piraeus, Greece.

PMID: 10530826  [PubMed - indexed for MEDLINE]

Click here for link to PubMed for article 10530826
```

----------------------------

----------------------------

```
Abstract 338 of 362

1. Stud Health Technol Inform. 1998;52 Pt 2:1278-81.

Virtual patients for a virtual hospital.

Loke E, Lun KC.

Medical Informatics Programme, National University of Singapore, Republic of
Singapore.

PMID: 10384665  [PubMed - indexed for MEDLINE]

Click here for link to PubMed for article 10384665
```

----------------------------

----------------------------

```
Abstract 339 of 362

1. Stud Health Technol Inform. 1998;52 Pt 2:732-5.

Using multimedia virtual patients to enhance the clinical curriculum for medical 
students.

McGee JB, Neill J, Goldman L, Casey E.

Harvard Medical School-Beth Israel Deaconess Mount Auburn Institute for Education
and Research, Boston, USA.

PMID: 10384556  [PubMed - indexed for MEDLINE]

Click here for link to PubMed for article 10384556
```

----------------------------

----------------------------

```
Abstract 340 of 362

1. Stud Health Technol Inform. 1998;52 Pt 1:21-5.

A quantitative perspective on the virtual patient record (VPR) and its
realization.

Möhr JR.

School of Health Information Science, University of Victoria, B.C., Canada.

PMID: 10384412  [PubMed - indexed for MEDLINE]

Click here for link to PubMed for article 10384412
```

----------------------------

----------------------------

```
Abstract 341 of 362

1. J Craniomaxillofac Surg. 1999 Apr;27(2):77-81.

Positioning of dental implants using computer-aided navigation and an optical
tracking system: case report and presentation of a new method.

Watzinger F, Birkfellner W, Wanschitz F, Millesi W, Schopper C, Sinko K, Huber K,
Bergmann H, Ewers R.

Clinic of Oral and Maxillofacial Surgery, Medical School, University of Vienna,
Vienna General Hospital, Vienna.

PMID: 10342142  [PubMed - indexed for MEDLINE]

Click here for link to PubMed for article 10342142
```

----------------------------

----------------------------

```
Abstract 342 of 362

1. Am J Med Qual. 1998 Fall;13(3):147-57.

Comparing the value of service between a state hospital and a private, for-profit
psychiatric hospital: a clarified role for tertiary care.

Davis GE, Lowell WE, Davis GL.

Augusta Mental Health Institute, ME 04332, USA.

PMID: 9735477  [PubMed - indexed for MEDLINE]

Click here for link to PubMed for article 9735477
```

----------------------------

----------------------------

```
Abstract 343 of 362

1. Eur J Ultrasound. 1998 Aug;7(3):225-300.

SONOSim3D: a multimedia system for sonography simulation and education with an
extensible case database.

Ehricke HH.

Polytechnical University of Stralsund, Medical Imaging and Computer Graphics Lab,
Fachhochschule Stralsund, Fachbereich Elektrotechnik und Informatik, Zur
Schwedenschanze 15, D-18435 Stralsund, Germany.

www.micg.et.fh-stralsund.de

Copyright 1998 Elsevier Science Ireland Ltd. All rights reserved.

PMID: 9700220  [PubMed - indexed for MEDLINE]

Click here for link to PubMed for article 9700220
```

----------------------------

----------------------------

```
Abstract 344 of 362

1. Int J Med Inform. 1997 Dec;47(3):175-82.

Approaches for certification of electronic prescription software.

Niinimäki J, Forsström J.

Health Care Informatics Centre of Excellence, Satakunta Hospital District, Pori, 
Finland.

PMID: 9513006  [PubMed - indexed for MEDLINE]

Click here for link to PubMed for article 9513006
```

----------------------------

----------------------------

```
Abstract 345 of 362

1. Med Inform (Lond). 1997 Oct-Dec;22(4):325-35.

A framework for the integration of distributed autonomous healthcare information 
systems.

Leisch E, Sartzetakis S, Tsiknakis M, Orphanoudakis SC.

Institute of Computer Science, Foundation for Research and Technology-Hellas,
Heraklion, Greece.

PMID: 9509403  [PubMed - indexed for MEDLINE]

Click here for link to PubMed for article 9509403
```

----------------------------

----------------------------

```
Abstract 346 of 362

1. Acad Med. 1997 Dec;72(12):1076-81.

Virtual reality: teaching tool of the twenty-first century?

Hoffman H, Vu D.

University of California, San Diego, School of Medicine, La Jolla, USA.

PMID: 9435714  [PubMed - indexed for MEDLINE]

Click here for link to PubMed for article 9435714
```

----------------------------

----------------------------

```
Abstract 347 of 362

1. Br J Oral Maxillofac Surg. 1997 Aug;35(4):271-4.

Computed intraoperative navigation guidance--a preliminary report on a new
technique.

Enislidis G, Wagner A, Ploder O, Ewers R.

University-Clinic for Maxillofacial Surgery, Vienna, Austria.

PMID: 9291266  [PubMed - indexed for MEDLINE]

Click here for link to PubMed for article 9291266
```

----------------------------

----------------------------

```
Abstract 348 of 362

1. Cancer Radiother. 1997;1(5):581-6.

[Practice of virtual simulation at the Saint-André hospital].

[Article in French]

Trouette R, Causse N, Maire JP, Dahan O, Récaldini L, Demeaux H, Baumont G,
Houlard JP, Caudry M.

Service de cancérologie, hôpital Saint-André, Bordeaux, France.

PMID: 9587392  [PubMed - indexed for MEDLINE]

Click here for link to PubMed for article 9587392
```

----------------------------

----------------------------

```
Abstract 349 of 362

1. Cancer Radiother. 1997;1(5):573-80.

[Virtual simulation: means and methodology].

[Article in French]

Beaudré A, Pica A.

Institut Gustave-Roussy, Villejuif, France.

PMID: 9587391  [PubMed - indexed for MEDLINE]

Click here for link to PubMed for article 9587391
```

----------------------------

----------------------------

```
Abstract 350 of 362

1. Comput Aided Surg. 1997;2(5):286-91.

VBH head holder to improve frameless stereotactic brachytherapy of cranial
tumors.

Bale RJ, Vogele M, Martin A, Auer T, Hensler E, Eichberger P, Freysinger W,
Sweeney R, Gunkel AR, Lukas PH.

Department of Radiology, University of Innsbruck, Austria.

PMID: 9484589  [PubMed - indexed for MEDLINE]

Click here for link to PubMed for article 9484589
```

----------------------------

----------------------------

```
Abstract 351 of 362

1. Eur Arch Otorhinolaryngol. 1997;254(7):343-6.

Image-guided endoscopic ENT surgery.

Freysinger W, Gunkel AR, Thumfart WF.

ENT Department, University of Innsbruck, Austria.

PMID: 9298670  [PubMed - indexed for MEDLINE]

Click here for link to PubMed for article 9298670
```

----------------------------

----------------------------

```
Abstract 352 of 362

1. Z Kardiol. 1997 Jan;86(1):35-41.

[Calibration of clinical databanks with "virtual patients"].

[Article in German]

Vahl CF, Carl I, de Simone R, Meinzer HP, Thomas G, Hagl S.

Klinik für Herzchirurgie, Heidelberg.

PMID: 9133122  [PubMed - indexed for MEDLINE]

Click here for link to PubMed for article 9133122
```

----------------------------

----------------------------

```
Abstract 353 of 362

1. Stud Health Technol Inform. 1997;39:518-28.

ENT endoscopic surgical training simulator.

Edmond CV Jr, Heskamp D, Sluis D, Stredney D, Sessanna D, Wiet G, Yagel R,
Weghorst S, Oppenheimer P, Miller J, Levin M, Rosenberg L.

Madigan Army Medical Center, Tacoma WA, USA.

PMID: 10173068  [PubMed - indexed for MEDLINE]

Click here for link to PubMed for article 10173068
```

----------------------------

----------------------------

```
Abstract 354 of 362

1. Stud Health Technol Inform. 1997;39:224-31.

Virtual reality in the operating room of the future.

Müller W, Grosskopf S, Hildebrand A, Malkewitz R, Ziegler R.

Fraunhofer Institute for Computer Graphics (Fraunhofer-IGD), Department
Visualization & Virtual Reality, Darmstadt, Germany.

PMID: 10173059  [PubMed - indexed for MEDLINE]

Click here for link to PubMed for article 10173059
```

----------------------------

----------------------------

```
Abstract 355 of 362

1. Kidney Int. 1996 Mar;49(3):823-32.

Quasi-steadiness approximation for the single-compartment urea kinetic model
(SCUKM).

Koike J, Sakuma Y, Shinohara S, Matsui N.

Department of Internal Medicine, Tsuchiura Kyodo General Hospital, Japan.

PMID: 8648926  [PubMed - indexed for MEDLINE]

Click here for link to PubMed for article 8648926
```

----------------------------

----------------------------

```
Abstract 356 of 362

1. Ala Med. 1996 Feb-Apr;65(8-10):9-13.

Medical information on the Internet.

Burnham J.

University of South Alabama, Biomedical Library, Alabama, USA.

PMID: 8871509  [PubMed - indexed for MEDLINE]

Click here for link to PubMed for article 8871509
```

----------------------------

----------------------------

```
Abstract 357 of 362

1. Proc AMIA Annu Fall Symp. 1996:483-7.

Experiences with a distributed virtual patient record system.

Forslund DW, Phillips RL, Kilman DG, Cook JL.

Los Alamos National Laboratory, New Mexico, USA.

PMCID: PMC2233197
PMID: 8947713  [PubMed - indexed for MEDLINE]

Click here for link to PubMed for article 8947713
```

----------------------------

----------------------------

```
Abstract 358 of 362

1. Technol Health Care. 1995 Oct;3(2):75-89.

Medicine in virtual environments.

Dumay AC.

TNO Physics and Electronics Laboratory, The Hague, The Netherlands.

PMID: 8574765  [PubMed - indexed for MEDLINE]

Click here for link to PubMed for article 8574765
```

----------------------------

----------------------------

```
Abstract 359 of 362

1. CMAJ. 1995 Apr 15;152(8):1303-7.

Navigating physician resources on the Internet.

Ellenberger B.

PMCID: PMC1337830
PMID: 7736378  [PubMed - indexed for MEDLINE]

Click here for link to PubMed for article 7736378
```

----------------------------

----------------------------

```
Abstract 360 of 362

1. Medinfo. 1995;8 Pt 2:1716.

A demonstration of the virtual nursing college.

Yensen JA, Woolery LK.

Nursing Faculty, Langara College, Vancouver, BC, Canada.

PMID: 8591569  [PubMed - indexed for MEDLINE]

Click here for link to PubMed for article 8591569
```

----------------------------

----------------------------

```
Abstract 361 of 362

1. Acta Otorhinolaryngol Belg. 1995;49(3):257-61.

Complete sphenoethmoidectomy and computer-assisted surgery.

Gunkel AR, Freysinger W, Thumfart WF, Pototschnig C.

ENT-Department, University of Innsbruck, Austria.

PMID: 7484144  [PubMed - indexed for MEDLINE]

Click here for link to PubMed for article 7484144
```

----------------------------

----------------------------

```
Abstract 362 of 362

1. Int J Radiat Oncol Biol Phys. 1991 Jul;21(2):475-82.

The portable virtual simulator.

Sherouse GW, Chaney EL.

Department of Radiation Oncology, University of North Carolina, Chapel Hill
27599-7512.

PMID: 2061124  [PubMed - indexed for MEDLINE]

Click here for link to PubMed for article 2061124
```

----------------------------
