# Supplementary material for: On the usage of health records for the design of virtual patients: a systematic review
Source: BMC Med Inform Decis Mak. 2013 Sep 8;13:103. doi: 10.1186/1472-6947-13-103 (PMC3846661; doi:10.1186/1472-6947-13-103)
Supplement: Additional file 2 — Table of reviewed articles. Each of the full-text articles reviewed in the final phase of the review are shown here (Table 2.docx). Here it can be seen if any standards were followed, the type of patient data used to create the Virtual Patient(s), and whether the platform described allowed for cases to be interchanged. Where no standards such as SCORM have been followed, the case format is provided, such as XML or PowerPoint. [file 1472-6947-13-103-S2.docx]

| **Author(s)** | **Interchangeable Cases?** | **Type of Patient Data** | **Standards Followed** | **Used for Undergraduate or Resident Teaching?** | **Notes** |
| --- | --- | --- | --- | --- | --- |
| Jacobson S, et al. [5] | Yes | CT/MRI | DICOM, OsiriX, QuickTime | Yes | Cases prepared using OsiriX, exported as QuickTime movie. |
| Parikh SS, et al. [6] | Yes | CT/MRI | DICOM | No | Cases were read by the Stanford Virtual Surgery Environment from 2D DICOM images. |
| King BW, et al. [7] | Yes | CT/MRI | - | No | Real CT/MRI data is used to construct 3D models. |
| Porro I, et al. [8] | Yes | CT/MRI | DICOM | Yes | - |
| Heer IM, et al. [9] | Yes | CT/MRI | - | Yes | Based on GE EchoTech system. |
| Michel MS, et al. [10] | Yes | CT/MRI | - | Yes | Used URO Mentor software. |
| Freysinger W, et al. [11] | Yes | CT/MRI | - | No | Used the ARTMA software. |
| Lamadé W. et al. [12] | No | CT/MRI | - | No | Used UltraPad A5 software. |
| Ehricke HH [13] | Yes | CT/MRI | - | Yes | Case database exists on CD-ROM and includes case descriptions. |
| Shyu FM, et al. [14] | Yes | Multiple | SCORM, DICOM, HL7 | Yes | Clinical cases are extracted from a hospital information system. |
| Trace C, et al. [15] | Yes | Multiple | PowerPoint | Yes | Student-authored electronic cases, made available in PowerPoint format. |
| Ullrich S, et al. [16] | Yes | MRI | XML | Yes | Uses an XML-based Virtual Patient database. |
| Oliven A, et al. [17] | Yes | Multiple | - | Yes | Uses a web-based Virtual Patient front-end. |
| Abendroth M, et al. [18] | Yes | Multiple | CASUS, Moodle | Yes | Based on the CASUS Virtual Patient system. |
| Pinnock R, et al. [19] | Yes | Multiple | XML | Yes | System developed reads XML-based data format. |
| Hörnlein A, et al. [20] | Yes | Multiple | Moodle, Word | Yes | Cases are authored in Word and then converted for online use. |
| Edelbring S, et al. [21] | Yes | Multiple | NUDOV | Yes | The ReumaCase system mentioned is based on NUDOV of the Karolinska Institute. |
| Adams EC, et al [22] | Yes | Video | Quandary | Yes | Used the Quandary system. |
| Poulton T, et al. [23] | Yes | Multiple | VUE, OpenLabyrinth | Yes | The OpenLabyrinth system was used. |
| Hooper LM, et al. [24] | No | Video | - | No | Cases were distributed on CD-ROM. |
| Vukanovic-Criley JM, et al. [25] | Yes | Multiple | - | Yes | Used actual heart recordings taken at the bedside. |
| Dewhurst D, et al. [26] | Yes | Multiple | Labyrinth | Yes | The Labyrinth authoring tool was used to create cases. |
| Schittek Janda M, et al. [27] | Yes | Multiple | - | Yes | Uses a custom made web-based front-end. |
| Wood E, et al. [28] | Yes | Multiple | Virtual Consulting Room | Yes | Allows junior doctors to follow a Virtual Patient of an actual patient previously under their care. |
| Smith SR, et al. [29] | Yes | Multiple | IVIMEDS Reusable Learning Objects | Yes | The IVIMEDS inter-school repository was used. |
| Gunning WT, et al. [30] | Yes | Multiple | Web-SP | Yes | The Web-SP platform was used for the Virtual Patient in question. |
| Courteille O, et al. [31] | Yes | Multiple | Interactive Simulation of Patients | Yes | The Interactive Simulation of Patients (ISP) tool was used. |
| Subramanian A, et al. [32] | Yes | Multiple | StepStone | Yes | The StepStone web-based medical learning software was used. |
